# Synthesis and Anti-Inflammatory Evaluation of Novel Hybrids of 7-Oxodehydroabietic Acid Bearing a 1,2,3-Triazole Moiety

**DOI:** 10.3390/molecules30030750

**Published:** 2025-02-06

**Authors:** Wen-Tao Fang, Yong-Feng Lv, Fu-Cai Ren, Hong Zhang, Dong-Mei Xie, Xiao-Bo Zhang, Cheng-Wu Fang, Shou-Jin Liu, Han Luo

**Affiliations:** 1 College of Pharmacy, Anhui University of Chinese Medicine, Hefei 230011, China; fangwentao@ahtcm.edu.cn (W.-T.F.); zhanghong@ahtcm.edu.cn (H.Z.); xiedongmei96@126.com (D.-M.X.); 2 College of Science, Westlake University, Hangzhou 310024, China; lyuyongfeng@westlake.edu.cn; 3 State Key Laboratory of Phytochemistry and Plant Resources in West China, Kunming Institute of Botany, Chinese Academy of Sciences, Kunming 650201, China; 4 College of Pharmacy, Anhui Medical University, Hefei 230032, China; renfucai@ahmu.edu.cn; 5 State Key Laboratory for Quality Ensurance and Sustainable Use of Dao-di Herbs, Beijng 100700, China; jack110007@163.com

**Keywords:** 7-oxodehydroabietic acid, triazoles, anti-inflammatory activity, synthesis

## Abstract

To discover novel, potent anti-inflammatory diterpenoids, a series of hybrids of 7-oxodehydroabietic acid bearing a 1,2,3-triazole moiety was designed and synthesized. The target compounds were characterized by means of ^1^H NMR, ^13^C NMR, and ESI-HRMS. All the compounds were evaluated for their anti-inflammatory activity towards BV2 cell lines using L-NMMA (IC_50_ = 42.36 ± 2.47 µM) as a positive control. Most showed good anti-inflammatory activities, especially compounds **10** (IC_50_ = 8.40 ± 0.98 µM), **15** (IC_50_ = 10.74 ± 2.67 µM), **16** (IC_50_ = 10.96 ± 1.85 µM), and **17** (IC_50_ = 9.76 ± 1.27 µM), which exhibited potent anti-inflammatory effects on BV2 cell lines.

## 1. Introduction

**Figure 1:**
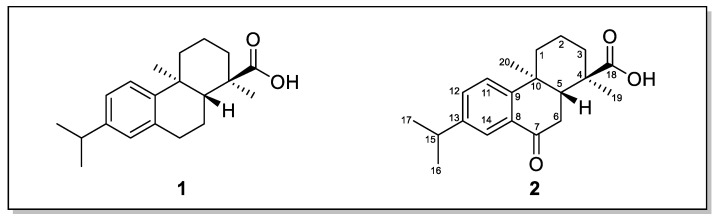
The structures of dehydroabietic acid (**1**) and 7-oxodehydroabietic acid (**2**).

Natural products have played an important role in drug discovery, given their chemical structural diversity, good biological activities, and biocompatibility [1]. Abietanes are a family of natural tricyclic diterpenoids with interesting pharmacological activity, including antitumor, antimicrobial, antiviral, antiulcer, and anti-inflammatory activities [2,3,4,5,6,7]. In particular, dehydroabietic acid (DHA) and its derivatives have been reported in recent years to show a broad spectrum of biological activities [8,9,10]. However, 7-oxodehydroabietic acid (Figure 1), a highly oxidized derivative of benzyl oxidation of dehydroabietic acid (DHA), has rarely been considered. Given its potent biological activities and limited exploration, we set out to introduce new groups in order to develop new compounds bearing anti-inflammatory activity based on 7-oxodehydroabietic acid.

The 1,4-disubstituted triazole moiety is well-known in medicinal chemistry for its stability, with resistance to oxidation, reduction, and metabolic degradation [11]. Therefore, 1,2,3-triazole is often used as a pharmacophore, which warrants consideration in the process of drug design [12,13]. Click chemistry is considered the most efficient means of synthesis for preparing 1,2,3-triazoles derivatives under mild conditions. In order to search for potent agents bearing anti-inflammatory activity, we envisaged synthesizing new compounds containing the 1,4-disubstituted triazole moiety based on 7-oxodehydroabietic acid.

In this study, in order to enrich the structures of existing compounds and find highly active target molecules, a series of compounds containing the 1,4-disubstituted triazole unit derived from 7-oxodehydroabietic acid were prepared. The anti-inflammatory activities of all synthesized compounds were assessed in vitro towards BV2 cell lines (mouse small glioma cells), using NG-methyl-L-arginine acetate salt (L-NMMA) [14,15], a well-known nitric oxide synthase (NOS) inhibitor, as a positive control. BV2 cells can produce a series of inflammatory factors and cytokines after infection and inflammatory stimulation, so they are widely used in inflammation-related research [16]. To our delight, a better anti-inflammatory effect (nitric oxide (NO) inhibitory activities) was found in promising compounds **10**, **15**, **16**, and **17**.

## 2. Results

### Chemistry

**Scheme 1:**
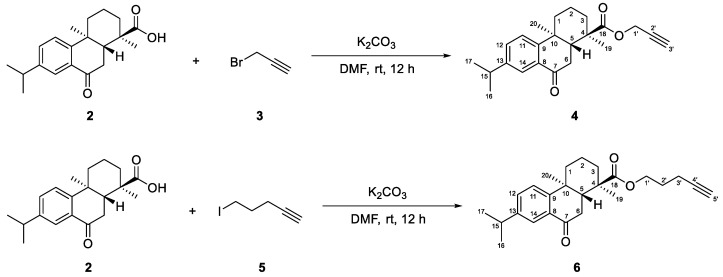
Preparation of O-propargylated 7-oxodehydroabietic acid (**4**) and O-pentynylated 7-oxodehydroabietic acid (**6**).

In this work, 7-oxodehydroabietic acid (**2**) was synthesized via benzyl oxidation of dehydroabietic acid (DHA), according to the reported literature [17]. In the presence of potassium carbonate, 7-oxodehydroabietic acid (**2**) was treated with 3-bromoprop-1-yne (**3**) or 5-iodopent-1-yne (**5**) in anhydrous DMF, yielding the corresponding desired products O-propargylated 7-oxodehydroabietic acid (**4**) and O-pentynylated 7-oxodehydroabietic acid (**6**) (Scheme 1).

**Scheme 2:**
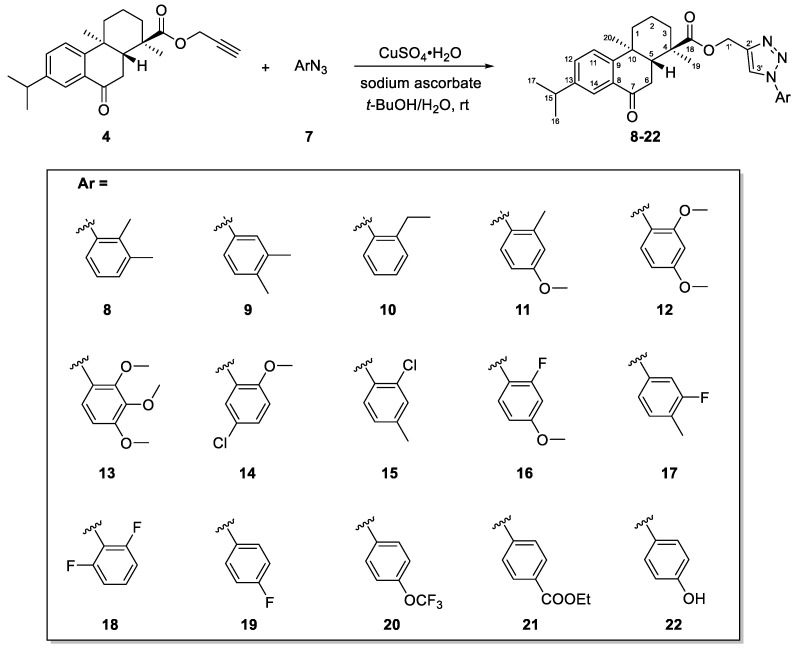
Synthesis of 7-oxodehydroabietic acid–1,2,3-triazole hybrids from O-propargylated 7-oxodehydroabietic acid (**4**).

**Scheme 3:**
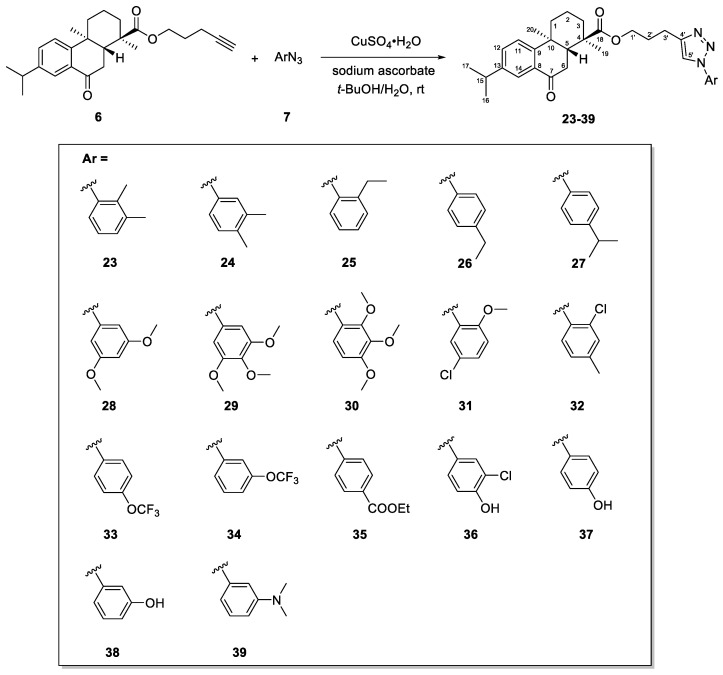
Synthesis of 7-oxodehydroabietic acid–1,2,3-triazole hybrids from O-pentynylated 7-oxodehydroabietic acid (**6**).

In order to introduce a 1,2,3-triazole unit based on the key intermediates O-propargylated 7-oxodehydroabietic acid (**4**) and O-pentynylated 7-oxodehydroabietic acid (**6**), we adopted a copper(I)-catalyzed Huisgen 1,3-dipolar cycloaddition reaction of O-propargylated 7-oxodehydroabietic acid (**4**) and O-pentynylated 7-oxodehydroabietic acid (**6**) with different substituted aromatic azides (Scheme 2 and Scheme 3) [18,19]. To our delight, the click reaction exhibited excellent functional group compatibility, and a series of derivatives bearing the 1,2,3-triazole unit were synthesized. All aromatic azides were prepared from corresponding boronic acid with sodium azide in the presence of CuSO_4_ in methanol (MeOH) without further purification [20,21,22].

7-oxodehydroabietic acid–1,2,3-triazole hybrids (**8**–**22**) from O-propargylated 7-oxodehydroabietic acid (**4**) and 7-oxodehydroabietic acid–1,2,3-triazole hybrids (**23**–**39**) from O-pentynylated 7-oxodehydroabietic acid (**6**) were produced efficiently. Compounds **8**–**40** are described for the first time, all presenting different substituents at the triazole moiety. The structures of all the compounds were confirmed by spectroscopic and spectrometric means (see Supplementary Materials). Next, we applied these derivatives bearing the 1,2,3-triazole unit, to evaluate their influence on the anti-inflammatory effect (NO inhibitory activities).

## 3. Discussion

Using L-NMMA (IC_50_ = 42.36 ± 2.47 µM) as a positive control, all derivatives of 7-oxodehydroabietic acid were tested for an anti-inflammatory effect (NO inhibitory activities) on BV2 cell lines. It was found, as shown in Table 1, that some of the newly synthesized compounds from O-propargylated 7-oxodehydroabietic acid (**4**) had a significant anti-inflammatory effect (NO inhibitory activities) on BV2 cell lines. This suggests that the length of the CH_2_ linker has an importance influence on the anti-inflammatory activity. In particular, compounds **10**–**12**, **14**–**17**, and **22** exhibited a good anti-inflammatory effect (NO inhibitory activities) on BV2 cell lines with IC_50_ values from 8.40 ± 0.98 µM to 15.82 ± 1.40 µM. The results were much better than those for the positive control L-NMMA (IC_50_ = 42.36 ± 2.47 µM). These data allowed us to carry out a simple structure and activity relationship (SAR) analysis on the influence of the modifications of different groups. Derivatives containing electron donors, such as a methoxy group or methyl group, and electron acceptors, such as a fluorine atom or chlorine atom in the aromatic ring, especially when most of the group was in the ortho and para positions, tended to have better anti-inflammatory activity. A phenolic hydroxyl group in the para position was beneficial for anti-inflammatory activity, while trifluoromethoxy and ester groups showed relatively poor results.

## 4. Materials and Methods

### 4.1. General Experimental Procedures

All reagents and solvents were purchased from Energy. All synthesized derivatives were purified by column chromatography (silica gel, petroleum ether/ethyl acetate, 10:1 to 1:1, and petroleum ether/acetone, 10:1 to 1:1) and their structures were elucidated by ^1^H NMR, ^13^C NMR, and high-resolution mass spectrometry (HR-ESIMS). Mass spectra were investigated on a UPLC-IT-TOF (Shimadzu, Kyoto, Japan) spectrometer. NMR spectra were recorded on Avance III 600 MHz (Bruker, Bremerhaven, Germany) instruments using CDCl_3_, CD_3_OD, or acetone-d6 as the solvent, with TMS as the internal standard. Chemical shifts (*δ*) were reported in parts per million (ppm), and the coupling constants (*J*) were given in Hertz. The abbreviations for the splitting of ^1^H NMR signals were as follows: s (singlet), d (doublet), t (triplet), q (quartet), and m (multiplet). Column chromatography was performed on silica gel (200–300 mesh, Qingdao Makall Group Co., Qingdao, China). All chemical reactions were monitored by TLC on silica gel 60 F254 plates, and spots were visualized by UV light and sprayed with 10% H_3_PO_4_·12MoO_3_ in EtOH, followed by heating. All compounds were named using the ACD40 Name-Pro program, which is based on IUPAC rules. Azides (7) were synthesized according to procedures previously described in the literature [20,21].

O-propargylated 7-oxodehydroabietic acid (**4**). To a solution of 7-oxodehydroabietic acid (**2**) (2.00 g, 6.37 mmol, 1.0 eq) in DMF (30 mL), were added K_2_CO_3_ (1.32 g, 9.55 mmol, 1.5 eq) slowly. The reaction mixture was stirred at rt for 30 min, and 3-bromoprop-1-yne (**3**) (0.67 mL, 7.64 mmol, 1.2 eq) was added dropwise at rt. The reaction mixture was stirred at rt for 24 h, before it was quenched by saturated NaCl aqueous solution (30 mL), and the mixture was extracted with ethyl acetate (3 × 30 mL). The combined organic layer was washed with brine (2 × 40 mL, and dried over Na_2_SO_4_, then filtered. After removing the solvent under a vacuum, the residue was purified by flash column chromatography on silica gel (10:1 to 5:1 petroleum ether/EtOAc), which provided O-propargylated 7-oxodehydroabietic acid (**4**) (1.68 g, 75% yield) as a white solid [23].

O-pentynylated 7-oxodehydroabietic acid (**6**). To a solution of 7-oxodehydroabietic acid (**2**) (2.00 g, 6.37 mmol, 1.0 eq) in DMF (30 mL), we added K_2_CO_3_ (1.32 g, 9.55 mmol, 1.5 eq) slowly. The reaction mixture was stirred at rt for 30 min, and 5-iodopent-1-yne (**5**) (0.87 mL, 7.64 mmol, 1.2 eq) was added dropwise at rt. The reaction mixture was stirred at rt for 24 h before it was quenched by saturated NaCl aqueous solution (30 mL), and the mixture was extracted with ethyl acetate (3 × 30 mL). The combined organic layer was washed with brine (2 × 40 mL) and dried over Na_2_SO_4_, then filtered. After removing the solvent under a vacuum, the residue was purified by flash column chromatography on silica gel (10:1 to 5:1 petroleum ether/EtOAc), which provided O-pentynylated 7-oxodehydroabietic acid (**6**) (1.70 g, 70% yield) as a white solid.

### 4.2. General Procedures for the Preparation of 7-Oxodehydroabietic Acid–1,2,3-Triazole Hybrids

To a solution of the corresponding azide (0.2 mmol) in 4 mL mixed solution (*t*-BuOH/H_2_O = 1:1, *v*/*v*), we added O-propargylated 7-oxodehydroabietic acid (**4**) or O-pentynylated 7-oxodehydroabietic acid (**6**) (0.2 mmol), sodium ascorbate (0.02 mmol), and CuSO_4_·5H_2_O (0.02 mmol). The reaction mixture was stirred for 48 h at room temperature before it was quenched by saturated NH_4_Cl aqueous solution (5 mL), and the mixture was extracted with ethyl acetate (3 × 6 mL). The combined organic layer was washed with brine (2 × 15 mL) and dried over Na_2_SO_4_, then filtered [24]. After removing the solvent under a vacuum, the residue was purified by flash column chromatography on silica gel (8/1 to 1/1 petroleum ether/EtOAc), which provided compounds **8**–**39**.

*18-O-(1-(2,3-Dimethylphenyl)-1H-1,2,3-triazol-4-yl)methyl-7-oxo-dehydroabietic acid* (**8**). Yield: 39%, yellow oil, ^1^H NMR (600 MHz, CDCl_3_) *δ* 7.84 (d, *J* = 1.9 Hz, 1H), 7.73 (s, 1H), 7.40 (dd, *J* = 8.1, 1.9 Hz, 1H), 7.31 (t, *J* = 7.7 Hz, 1H), 7.28 (d, *J* = 8.1 Hz, 1H), 7.23 (t, *J* = 7.7 Hz, 1H), 7.18 (d, *J* = 7.7 Hz, 1H), 5.31 (d, *J* = 2.3 Hz, 2H), 2.92 (hept, *J* = 6.9 Hz, 1H), 2.65–2.76 (m, 2H), 2.37 (s, 3H), 2.24–2.36 (m, 2H), 2.00 (s, 3H), 1.79 (t, *J* = 10.3 Hz, 3H), 1.73 (d, *J* = 6.1 Hz, 1H), 1.59–1.64 (m, 1H), 1.35 (s, 3H), 1.25 (s, 3H), 1.24 (d, *J* = 2.1 Hz, 3H), 1.23 (d, *J* = 2.1 Hz, 3H); ^13^C NMR (150 MHz, CDCl_3_) *δ* 198.2, 177.2, 152.8, 146.9, 142.5, 138.7, 136.3, 132.6, 132.5, 131.4, 130.6, 126.1, 125.6, 125.0, 124.0, 123.4, 58.0, 46.6, 43.6, 37.8, 37.2, 37.0, 36.3, 33.5, 23.8, 23.7, 23.6, 20.3,18.0, 16.3, 14.2; HRESIMS: calcd for C_31_H_37_N_3_O_3_ [M + H]^+^ 500.2913, found 500.2914 (mass error ∆m = −0.0001 ppm).

*18-O-(1-(3,4-Dimethylphenyl)-1H-1,2,3-triazol-4-yl)methyl-7-oxo-dehydroabietic acid* (**9**). Yield: 66%, white oil, ^1^H NMR (600 MHz, CDCl_3_) *δ* 7.96 (s, 1H), 7.85 (d, *J* = 2.0 Hz, 1H), 7.53 (d, *J* = 1.7 Hz, 1H), 7.43 (dd, *J* = 8.1, 2.1 Hz, 1H), 7.39 (dd, *J* = 8.1, 2.0 Hz, 1H), 7.28 (d, *J* = 8.2 Hz, 1H), 7.25 (s, 1H), 5.28 (s, 2H), 2.91 (hept, *J* = 6.9 Hz, 1H), 2.66–2.76 (m, 2H), 2.34 (s, 2H), 2.31– 2.33 (m, 2H), 2.31 (s, 2H), 1.78 (t, *J* = 11.6 Hz, 3H), 1.68–1.74 (m, 1H), 1.58–1.65 (m, 1H), 1.35 (s, 3H), 1.25 (s, 3H), 1.24 (d, *J* = 2.2 Hz, 3H), 1.23 (d, *J* = 2.2 Hz, 3H); ^13^C NMR (150 MHz, CDCl_3_) *δ* 198.3, 177.1, 152.8, 146.8, 143.2, 138.3, 137.6, 134.7, 132.5, 130.5, 130.5, 124.9, 123.5, 121.8, 121.7, 117.9, 58.0, 46.6, 43.6, 37.8, 37.3, 36.9, 36.3, 33.5, 23.7, 23.7, 23.6, 19.8, 19.4, 18.0, 16.3; HRESIMS: calcd for C_31_H_37_N_3_O_3_ [M + H]^+^ 500.2913, found 500.2914 (mass error ∆m = −0.0001 ppm).

*18-O-(1-(2-Ethylphenyl)-1H-1,2,3-triazol-4-yl)methyl-7-oxo-dehydroabietic acid* (**10**). Yield: 43%, yellow oil, ^1^H NMR (600 MHz, CDCl_3_) *δ* 7.84 (d, *J* = 2.0 Hz, 1H), 7.75 (s, 1H), 7.40 (dd, *J* = 8.1, 2.0 Hz, 1H), 7.29 (d, *J* = 8.1 Hz, 1H), 7.24 (d, *J* = 7.9 Hz, 1H), 7.22 (d, *J* = 8.1 Hz, 1H), 7.19 (s, 1H), 5.31 (d, *J* = 2.2 Hz, 2H), 2.92 (hept, *J* = 6.9 Hz, 1H), 2.71 (d, *J* = 14.4 Hz, 2H), 2.39 (s, 3H), 2.32 (m, 2H), 2.15 (s, 3H), 1.80 (t, *J* = 10.2 Hz, 3H), 1.70–1.75 (m, 1H), 1.61–1.64 (m, 1H), 1.36 (s, 3H), 1.26 (s, 3H), 1.25 (d, *J* = 1.4 Hz, 3H), 1.24 (d, *J* = 1.4 Hz, 3H); ^13^C NMR (150 MHz, CDCl_3_) *δ* 198.2, 177.2, 152.8, 146.9, 142.5, 136.8, 136.0, 132.5, 131.2, 130.6, 130.2, 126.5, 125.2, 125.0, 123.4, 58.0, 46.6, 43.6, 37.8, 37.2, 37.0, 36.3, 33.5, 29.6, 23.7, 23.7, 23.6, 20.6, 18.0, 17.3, 16.3; HRESIMS: calcd for C_31_H_37_N_3_O_3_ [M + H]^+^ 500.2913, found 500.2918 (mass error ∆m = −0.0005 ppm).

*18-O-(1-(4-Methoxy-2-methylphenyl)-1H-1,2,3-triazol-4-yl)methyl-7-oxo-dehydroabietic acid* (**11**). Yield: 40%, brown oil, ^1^H NMR (600 MHz, CDCl_3_) *δ* 7.84 (d, *J* = 2.2 Hz, 1H), 7.71 (s, 1H), 7.40 (dd, *J* = 8.1, 2.2 Hz, 1H), 7.28 (d, *J* = 8.1 Hz, 2H), 6.83–6.87 (m, 2H), 5.30 (s, 2H), 3.86 (s, 3H), 2.92 (p, *J* = 6.9 Hz, 1H), 2.71 (d, *J* = 14.9 Hz, 2H), 2.32–2.37 (m, 1H), 2.27 (d, *J* = 14.5 Hz, 1H), 2.14 (s, 3H), 1.76–1.84 (m, 3H), 1.74–1.70 (m, 1H), 1.64 (dd, *J* = 12.6, 4.5 Hz, 1H), 1.36 (s, 3H), 1.24–1.26 (m, 6H), 1.24 (d, *J* = 1.4 Hz, 3H); ^13^C NMR (150 MHz, CDCl_3_) *δ* 198.3, 177.3, 160.4, 152.9, 147.0, 135.4, 132.6, 130.7, 129.5, 127.4, 125.6, 125.0, 123.5, 116.4, 111.9, 58.1, 55.6, 46.7, 43.7, 37.8, 37.3, 37.0, 36.4, 33.6, 29.7, 23.8, 23.8, 23.7, 18.1, 18.0, 16.4; HRESIMS: calcd for C_31_H_37_N_3_O_4_ [M + H]^+^ 516.2862, found 516.2867 (mass error ∆m = −0.0005 ppm).

*19-O-(1-(2,4-Dimethoxyphenyl)-1H-1,2,3-triazol-4-yl)methyl-7-oxo-dehydroabietic acid* (**12**). Yield: 48%, yellow oil, ^1^H NMR (600 MHz, CDCl_3_) *δ* 8.00 (s, 1H), 7.84 (d, *J* = 2.2 Hz, 1H), 7.65 (m, 1H), 7.39 (dd, *J* = 8.2, 2.2 Hz, 1H), 7.28 (d, *J* = 8.2 Hz, 1H), 6.61 (d, *J* = 7.4 Hz, 2H), 5.29 (m, 2H), 3.86 (s, 3H), 3.84 (s, 3H), 2.91 (hept, *J* = 6.8 Hz, 1H), 2.63–2.79 (m, 2H), 2.33 (m, 2H), 1.80 (d, *J* = 9.4 Hz, 4H), 1.65 (d, *J* = 4.4 Hz, 1H), 1.35 (s, 3H), 1.24–1.26 (m, 6H), 1.23 (d, *J* = 1.4 Hz, 3H); ^13^C NMR (150 MHz, CDCl_3_) *δ* 198.3, 177.2, 161.2, 152.8, 152.5, 146.9, 141.9, 132.5, 130.6, 126.5, 125.9, 125.0, 123.4, 119.7, 104.7, 99.5, 58.1, 55.9, 55.6, 46.5, 43.5, 37.7, 37.2, 36.9, 36.4, 33.5, 23.8, 23.7, 23.7, 18.1, 16.3; HRESIMS: calcd for C_31_H_37_N_3_O_5_ [M + H]^+^ 532.2811, found 532.2818 (mass error ∆m = −0.0007 ppm).

*18-O-(1-(2,3,4-Trimethoxyphenyl)-1H-1,2,3-triazol-4-yl)methyl-7-oxo-dehydroabietic acid* (**13**). Yield: 49%, brown oil, ^1^H NMR (600 MHz, CDCl_3_) *δ* 8.01 (s, 1H), 7.83 (d, *J* = 2.0 Hz, 1H), 7.43 (d, *J* = 8.9 Hz, 1H), 7.39 (dd, *J* = 8.2, 1.9 Hz, 1H), 7.27 (d, *J* = 9.0 Hz, 1H), 6.78 (d, *J* = 8.9 Hz, 1H), 5.31 (m, 2H), 3.93(s, 9H), 2.91 (hept, *J* = 6.9 Hz, 1H), 2.64–2.76 (m, 2H), 2.32 (m, 2H), 1.77 (s, 3H), 1.72 (d, *J* = 5.5 Hz, 1H), 1.62 (m, 1H), 1.35 (s, 3H), 1.25 (s, 3H), 1.24 (d, *J* = 1.7 Hz, 3H), 1.23 (d, *J* = 1.7 Hz, 3H); ^13^C NMR (150 MHz, CDCl_3_) *δ* 198.2, 177.1, 154.4, 152.8, 146.9, 146.6, 142.6, 142.4, 132.5, 130.6, 125.5, 125.0, 124.2, 123.4, 120.0, 107.2, 61.4, 61.1, 58.0, 56.2, 46.6, 43.6, 37.7, 37.2, 37.0, 36.4, 33.5, 23.7, 23.7, 23.6, 18.1, 16.3; HRESIMS: calcd for C_32_H_39_N_3_O_6_ [M + H]^+^ 562.2917, found 562.2918 (mass error ∆m = −0.0001 ppm).

*19-O-(1-(5-Chloro-2-methoxyphenyl)-1H-1,2,3-triazol-4-yl)methyl-7-oxo-dehydroabietic acid* (**14**). Yield: 36%, yellow oil, ^1^H NMR (600 MHz, CDCl_3_) *δ* 8.16 (s, 1H), 7.87 (d, *J* = 2.6 Hz, 1H), 7.84 (d, *J* = 2.1 Hz, 1H), 7.38 (ddd, *J* = 11.4, 8.5, 2.6 Hz, 2H), 7.28 (d, *J* = 8.2 Hz, 1H), 7.01 (d, *J* = 8.5 Hz, 1H), 5.20–5.34 (m, 2H), 3.89 (s, 3H), 2.91 (p, *J* = 6.9 Hz, 1H), 2.63–2.78 (m, 2H), 2.33 (m, 2H), 1.74–1.84 (m, 3H), 1.72 (q, *J* = 3.0 Hz, 1H), 1.62 (td, *J* = 12.5, 4.1 Hz, 1H), 1.35 (s, 3H), 1.24–1.25 (m, 6H), 1.23 (d, *J* = 1.8 Hz, 3H); ^13^C NMR (150 MHz, CDCl_3_) *δ* 198.4, 177.2, 152.9, 147.0, 142.4, 132.6, 130.7, 129.8, 126.7, 126.2, 125.8, 125.3, 125.0, 123.5, 113.3, 58.0, 56.4, 46.6, 43.6, 37.8, 37.3, 37.0, 36.5, 33.6, 23.8, 23.8, 23.7, 18.1, 16.4; HRESIMS: calcd for C_30_H_34_ClN_3_O_4_ [M + H]^+^ 536.2316, found 536.2319 (mass error ∆m = −0.0003 ppm).

*18-O-(1-(2-Chloro-4-methylphenyl)-1H-1,2,3-triazol-4-yl)methyl-7-oxo-dehydroabietic acid* (**15**). Yield: 40%, yellow oil, ^1^H NMR (600 MHz, CDCl_3_) *δ* 7.96 (s, 1H), 7.84 (d, *J* = 2.2 Hz, 1H), 7.52 (d, *J* = 8.0 Hz, 1H), 7.39 (dd, *J* = 8.0, 2.1 Hz, 1H), 7.37 (d, *J* = 1.7 Hz, 1H), 7.27 (d, *J* = 8.2 Hz, 1H), 7.24 (dd, *J* = 8.2, 1.7 Hz, 1H), 5.30 (s, 2H), 2.91 (p, *J* = 7.0 Hz, 1H), 2.61–2.75 (m, 2H), 2.42 (s, 3H), 2.32–2.36 (m, 1H), 2.27 (d, *J* = 15.7, 1.2 Hz, 1H), 1.71–1.81 (m, 4H), 1.63 (m, 1H), 1.35 (s, 3H), 1.24 (m, 6H), 1.23 (d, *J* = 1.3 Hz, 3H); ^13^C NMR (150 MHz, CDCl_3_) *δ* 198.3, 177.2, 152.9, 146.9, 141.6, 132.6, 132.2, 131.0, 130.7, 128.6, 128.3, 127.5, 125.9, 125.1, 123.5, 57.9, 46.6, 43.7, 37.8, 37.3, 37.0, 36.4, 33.6, 23.8, 23.8, 23.7, 21.1, 18.1, 16.4; HRESIMS: calcd for C_30_H_34_ClN_3_O_3_ [M + H]^+^ 520.2367, found 520.2369 (mass error ∆m = −0.0002 ppm).

*18-O-(1-(2-Fluoro-4-methoxyphenyl)-1H-1,2,3-triazol-4-yl)methyl-7-oxo-dehydroabietic acid* (**16**). Yield: 53%, yellow oil, ^1^H NMR (600 MHz, CDCl_3_) *δ* 7.99 (d, *J* = 2.3 Hz, 1H), 7.84 (d, *J* = 2.0 Hz, 1H), 7.79 (t, *J* = 8.7 Hz, 1H), 7.39 (dd, *J* = 8.1, 2.0 Hz, 1H), 7.28 (d, *J* = 8.1 Hz, 1H), 6.85 (m, 1H), 6.81 (dd, *J* = 12.4, 2.6 Hz, 1H), 5.31 (d, *J* = 12.8 Hz, 1H), 5.27 (d, *J* = 12.8 Hz, 1H), 3.87 (s, 3H), 2.92 (hept, *J* = 6.9 Hz, 1H), 2.66–2.76 (m, 2H), 2.31 (t, *J* = 13.9 Hz, 2H), 1.79 (d, *J* = 9.7 Hz, 3H), 1.70–1.75 (m, 1H), 1.61–1.65 (m, 1H), 1.35 (s, 3H), 1.25 (s, 3H), 1.25 (s, 3H), 1.23–1.24 (m, 3H); ^13^C NMR (150 MHz, CDCl_3_) *δ* 198.3, 177.1, 161.0 (d, *J*_CF_ = 10.2 Hz), 154.4 (d, *J*_CF_ = 250.8 Hz), 152.8, 146.9, 132.5, 130.6, 125.9, 125.0, 123.4, 118.3 (d, *J*_CF_ = 10.9 Hz), 110.7 (d, *J*_CF_ = 3.2 Hz), 102.5 (d, *J*_CF_ = 23.2 Hz), 57.9, 55.9, 46.6, 43.6, 37.7, 37.2, 36.9, 36.3, 33.5, 23.8, 23.7, 23.6, 18.0, 16.3; HRESIMS: calcd for C_30_H_34_FN_3_O_4_ [M + H]^+^ 520.2612, found 520.2616 (mass error ∆m = −0.0004 ppm).

*18-O-(1-(3-Fluoro-4-methylphenyl)-1H-1,2,3-triazol-4-yl)methyl-7-oxo-dehydroabietic acid* (**17**). Yield: 53%, brown oil, ^1^H NMR (600 MHz, CDCl_3_) *δ* 7.98 (s, 1H), 7.85 (d, *J* = 1.9 Hz, 1H), 7.49 (dd, *J* = 9.9, 1.9 Hz, 1H), 7.43 (dd, *J* = 8.2, 2.2 Hz, 1H), 7.40 (dd, *J* = 8.2, 2.2 Hz, 1H), 7.34 (t, *J* = 8.2 Hz, 1H), 7.28 (d, *J* = 8.2 Hz, 1H), 5.30 (d, *J* = 12.8 Hz, 1H), 5.26 (d, *J* = 12.8 Hz, 1H), 2.92 (hept, *J* = 6.9 Hz, 1H), 2.66–2.79 (m, 2H), 2.33–2.35 (m, 3H), 2.10–2.32 (m, 2H), 1.75–1.81 (m, 3H), 1.72 (d, *J* = 6.2 Hz, 1H), 1.61 (m, 1H), 1.35 (s, 3H), 1.26 (s, 3H), 1.24 (d, *J* = 2.4 Hz, 3H), 1.23 (d, *J* = 2.4 Hz, 3H); ^13^C NMR (150 MHz, CDCl_3_) *δ* 198.4, 177.2, 161.2 (d, *J*_CF_ = 247.3 Hz), 152.8, 146.9, 135.6 (d, *J*_CF_ = 9.9 Hz), 132.6, 132.3 (d, *J*_CF_ = 6.3 Hz), 130.5, 125.8 (d, *J*_CF_ = 17.4 Hz), 125.0, 123.5, 121.9, 115.7 (d, *J*_CF_ = 3.6 Hz), 108.1(d, *J*_CF_ = 27.2 Hz), 57.9, 46.6, 43.7, 37.9, 37.3, 37.0, 36.3, 33.5, 23.7, 23.7, 23.5, 18.0, 16.3, 14.3; HRESIMS: calcd for C_30_H_34_FN_3_O_3_ [M + H]^+^ 504.2662, found 504.2664 (mass error ∆m = −0.0002 ppm).

*18-O-(1-(2,6-Difluorophenyl)-1H-1,2,3-triazol-4-yl)methyl-7-oxo-dehydroabietic acid* (**18**). Yield: 64%, yellow oil, ^1^H NMR (600 MHz, CDCl_3_) *δ* 7.86 (s, 1H), 7.84 (d, *J* = 2.1 Hz, 1H), 7.49 (tt, *J* = 8.6, 6.0 Hz, 1H), 7.39 (dd, *J* = 8.1, 2.1 Hz, 1H), 7.28 (d, *J* = 8.1 Hz, 1H), 7.14 (t, *J* = 8.1, 2H), 5.34 (d, *J* = 12.8 Hz, 1H), 5.29 (d, *J* = 12.8 Hz, 1H), 2.92 (hept, *J* = 7.0 Hz, 1H), 2.71 (d, *J* = 13.6 Hz, 2H), 2.31–2.38 (m, 2H), 1.74–1.81 (m, 3H), 1.70–1.74 (m, 1H), 1.63 (dd, *J* = 14.3, 10.3 Hz, 1H), 1.35 (s, 3H), 1.25 (s, 3H), 1.24 (d, *J* = 1.2 Hz, 3H), 1.23 (d, *J* = 1.2 Hz, 3H); ^13^C NMR (150 MHz, CDCl_3_) *δ* 198.3, 177.2, 156.7 (d, *J*_CF_ = 257.1 Hz), 156.7 (d, *J*_CF_ = 257.1 Hz), 152.8, 142.7, 132.5, 131.4 (t, *J*_CF_ = 9.5 Hz), 130.6, 126.4, 125.0, 123.4, 115.0(t, *J*_CF_ = 15.3 Hz), 112.5 (dd, *J*_CF_ = 19.7 Hz), 112.5 (dd, *J*_CF_ = 19.7 Hz), 57.7, 46.5, 43.5, 37.2, 37.2, 36.9, 36.4, 33.5, 23.8, 23.7, 23.6, 18.0, 16.3; HRESIMS: calcd for C_29_H_31_F_2_N_3_O_3_ [M + H]^+^ 508.2412, found 508.2413 (mass error ∆m = −0.0001 ppm).

*18-O-(1-(4-Fluorophenyl)-1H-1,2,3-triazol-4-yl)methyl-7-oxo-dehydroabietic acid* (**19**). Yield: 53%, yellow oil, ^1^H NMR (600 MHz, CDCl_3_) *δ* 7.97 (s, 1H), 7.84 (d, *J* = 1.8 Hz, 1H), 7.75 (m, 2H), 7.40 (dd, *J* = 8.1, 1.8 Hz, 1H), 7.28 (d, *J* = 8.1 Hz, 1H), 7.24 (d, *J* = 8.6 Hz, 1H), 5.32 (d, *J* = 12.8 Hz, 1H), 5.27 (d, *J* = 12.8 Hz, 1H), 2.91 (hept, *J* = 6.9 Hz, 1H), 2.67–2.76 (m, 2H), 2.24–2.38 (m, 2H), 1.75–1.82 (m, 3H), 1.72 (d, *J* = 6.1 Hz, 1H), 1.58–1.65 (m, 1H), 1.35 (s, 3H), 1.26 (s, 3H), 1.24 (d, *J* = 2.1 Hz, 3H), 1.23 (d, *J* = 2.1 Hz, 3H); ^13^C NMR (150 MHz, CDCl_3_) *δ* 198.4, 177.2, 162.5 (d, *J*_CF_ = 249.5 Hz), 152.8, 146.9, 143.6, 133.1, 132.6, 130.5, 124.9, 123.5, 122.7 (d, *J*_CF_ = 8.7 Hz), 122.7 (d, *J*_CF_ = 8.7 Hz), 122.0, 116.6 (d, *J*_CF_ = 23.0 Hz), 116.6 (d, *J*_CF_ = 23.0 Hz), 57.9, 46.6, 43.7, 37.9, 37.3, 37.0, 36.3, 33.5, 23.7, 23.7, 23.5, 18.0, 16.3; HRESIMS: calcd for C_29_H_32_FN_3_O_3_ [M + H]^+^ 490.2506, found 490.2512 (mass error ∆m = −0.0006 ppm).

*18-O-(1-(4-(Trifluoromethoxy)phenyl)-1H-1,2,3-triazol-4-yl)methyl-7-oxo-dehydroabietic acid* (**20**). Yield: 62%, yellow oil, ^1^H NMR (600 MHz, CDCl_3_) *δ* 8.01 (s, 1H), 7.84 (m, 3H), 7.41 (m, 3H), 7.28 (d, *J* = 8.2 Hz, 1H), 5.34 (d, *J* = 12.8 Hz, 1H), 5.27 (d, *J* = 12.8 Hz, 1H), 2.91 (h, *J* = 6.9 Hz, 1H), 2.68–2.76 (m, 2H), 2.24–2.39 (m, 2H), 1.79 (t, *J* = 11.3 Hz, 3H), 1.72 (d, *J* = 6.1 Hz, 1H), 1.57–1.65 (m, 1H), 1.35 (s, 3H), 1.26 (s, 3H), 1.24 (d, *J* = 2.3 Hz, 3H), 1.23 (d, *J* = 2.3 Hz, 3H); ^13^C NMR (150 MHz, CDCl_3_) *δ* 198.4, 177.1, 152.8, 149.1, 146.9, 143.8, 135.2, 132.7, 130.5, 124.9, 123.6, 122.2, 122.1, 121.9, 120.3 (q, *J*_CF_ = 258.4 Hz), 57.9, 46.6, 43.8, 38.0, 37.4, 37.0, 36.3, 33.5, 23.7, 23.7, 23.5, 18.0, 16.3; HRESIMS: calcd for C_30_H_32_F_3_N_3_O_4_ [M + H]^+^ 556.2423, found 556.2417 (mass error ∆m = 0.0006 ppm).

*18-O-(1-(4-(Ethoxycarbonyl)phenyl)-1H-1,2,3-triazol-4-yl)methyl-7-oxo-dehydroabietic acid* (**21**). Yield: 61%, yellow oil, ^1^H NMR (600 MHz, CDCl_3_) *δ* 8.23 (d, *J* = 8.7 Hz, 2H), 8.08 (s, 1H), 7.88 (d, *J* = 8.7 Hz, 2H), 7.85 (d, *J* = 2.0 Hz, 1H), 7.40 (dd, *J* = 8.1, 2.0 Hz, 1H), 7.28 (d, *J* = 8.1 Hz, 1H), 5.33 (d, *J* = 12.8 Hz, 1H), 5.28 (d, *J* = 12.8 Hz, 1H), 4.42 (q, *J* = 7.1 Hz, 2H), 2.91 (hept, *J* = 6.9 Hz, 1H), 2.68–2.77 (m, 2H), 2.26–2.39 (m, 2H), 1.75–1.82 (m, 3H), 1.72 (m, 1H), 1.61 (m, 1H), 1.42 (t, *J* = 7.1 Hz, 3H), 1.35 (s, 3H), 1.26 (s, 3H), 1.24 (d, *J* = 2.5 Hz, 3H), 1.23 (d, *J* = 2.5 Hz, 3H); ^13^C NMR (150 MHz, CDCl_3_) *δ* 198.4, 177.1, 165.3, 152.8, 146.9, 143.9, 139.8, 132.6, 131.2, 130.6, 130.5, 124.9, 123.5, 121.7, 120.0, 61.3, 57.9, 46.6, 43.7, 38.0, 37.3, 37.0, 36.3, 33.5, 23.7, 23.7, 23.5, 18.0, 16.3, 14.3; HRESIMS: calcd for C_32_H_37_N_3_O_5_ [M + H]^+^ 544.2811, found 544.2814 (mass error ∆m = −0.0003 ppm).

*18-O-(1-(4-Hydroxyphenyl)-1H-1,2,3-triazol-4-yl)methyl-7-oxo-dehydroabietic acid* (**22**). Yield: 54%, yellow oil, ^1^H NMR (600 MHz, CDCl_3_) *δ* 7.94 (s, 1H), 7.84 (d, *J* = 2.0 Hz, 1H), 7.51 (d, *J* = 8.7 Hz, 2H), 7.39 (dd, *J* = 8.2, 2.0 Hz, 1H), 7.27 (d, *J* = 8.2 Hz, 1H), 7.00 (d, *J* = 8.7 Hz, 2H), 5.29 (d, *J* = 12.8 Hz, 1H), 5.26 (d, *J* = 12.8 Hz, 1H), 4.12 (q, *J* = 7.1 Hz, 1H), 2.89 (p, *J* = 6.9 Hz, 1H), 2.72 (dd, *J* = 7.2, 3.6 Hz, 2H), 2.20–2.41 (m, 2H), 1.71–1.82 (m, 4H), 1.59 (m, 1H), 1.34 (s, 3H), 1.24 (s, 3H), 1.22 (d, *J* = 1.5 Hz, 3H), 1.21 (d, *J* = 1.5 Hz, 3H); ^13^C NMR (150 MHz, CDCl_3_) *δ* 199.3, 177.3, 157.6, 153.1, 147.0, 133.0, 130.4, 129.5, 125.1, 123.7, 122.6, 122.3, 116.5, 57.9, 46.7, 43.8, 37.9, 37.4, 37.0, 36.4, 33.6, 23.8, 23.7, 23.6, 18.1, 16.4; HRESIMS: calcd for C_29_H_33_N_3_O_4_ [M + H]^+^ 488.2549, found 488.2555 (mass error ∆m = −0.0006 ppm).

*18-O-(1-(2,3-Dimethylphenyl)-1H-1,2,3-triazol-4-yl)propyl-7-oxo-dehydroabietic acid* (**23**). Yield: 61.5%, yellow oil, ^1^H NMR (600 MHz, CDCl_3_) *δ* 7.85 (d, *J* = 2.0 Hz, 1H), 7.52 (s, 1H), 7.41 (dt, *J* = 8.1, 2.0 Hz, 1H), 7.30 (dd, *J* = 7.7, 4.3 Hz, 2H), 7.21 (t, *J* = 7.7 Hz, 1H), 7.15 (d, *J* = 7.7 Hz, 1H), 4.17 (t, *J* = 6.4 Hz, 2H), 2.92 (p, *J* = 7.0, Hz, 1H), 2.84 (t, *J* = 7.7 Hz, 2H), 2.74 (m, 2H), 2.38 (m, 1H), 2.37 (s, 3H), 2.10 (m, 2H), 2.02 (s, 3H), 1.72–1.83 (m, 4H), 1.65 (m, 1H), 1.35 (s, 3H), 1.27 (s, 3H), 1.24 (d, *J* = 2.1 Hz, 3H), 1.23 (d, *J* = 2.1 Hz, 3H); ^13^C NMR (150 MHz, CDCl_3_) *δ* 198.6, 177.4, 153.2, 147.1, 146.6, 138.8, 136.9, 132.9, 132.8, 131.3, 130.8, 126.2, 125.2, 124.1, 123.7, 123.2, 64.2, 46.9, 44.0, 38.1, 37.5, 37.2, 36.7, 33.7, 28.4, 23.9, 23.9, 23.8, 22.3, 20.5, 18.3, 16.5, 14.4; HRESIMS: calcd for C_33_H_42_N_3_O_3_ [M + H]^+^ 528.3226, found 528.3228 (mass error ∆m = −0.0002 ppm).

*18-O-(1-(3,4-Dimethylphenyl)-1H-1,2,3-triazol-4-yl)propyl-7-oxo-dehydroabietic acid* (**24**). Yield: 51.5%, yellow oil, ^1^H NMR (600 MHz, CDCl_3_) *δ* 7.87 (d, *J* = 1.7 Hz, 1H), 7.79 (s, 1H), 7.54 (s, 1H), 7.43 (s, 1H), 7.41 (dd, *J* = 8.2, 1.7 Hz, 1H), 7.30 (d, *J* = 8.2 Hz, 1H), 7.24 (d, *J* = 8.2 Hz, 1H), 4.15 (t, *J* = 6.2 Hz, 2H), 2.92 (p, *J* = 6.9 Hz, 1H), 2.82 (t, *J* = 7.3 Hz, 2H), 2.74 (d, *J* = 10.9 Hz, 2H), 2.37 (m, 2H), 2.34 (s, 3H), 2.31 (s, 3H), 2.08 (m, 2H), 1.72–1.85 (m, 4H), 1.63 (m, 1H), 1.35 (s, 3H), 1.27 (s, 3H), 1.25 (d, *J* = 2.2 Hz, 3H), 1.24 (d, *J* = 2.2 Hz, 3H); ^13^C NMR (150 MHz, CDCl_3_) *δ* 198.7, 177.5, 153.2, 147.1, 138.4, 137.3, 135.3, 132.8, 130.8, 130.7, 125.2, 123.7, 121.8, 119.5, 117.9, 64.2, 46.9, 44.1, 38.1, 37.6, 37.3, 36.7, 33.7, 28.4, 23.9, 23.9, 23.8, 22.4, 20.0, 19.6, 18.4, 16.5; HRESIMS: calcd for C_33_H_42_N_3_O_3_ [M + H]^+^ 528.3226, found 528.3228 (mass error ∆m = −0.0002 ppm).

*18-O-(1-(2-Ethylphenyl)-1H-1,2,3-triazol-4-yl)propyl-7-oxo-dehydroabietic acid* (**25**). Yield: 54.3%, yellow oil, ^1^H NMR (600 MHz, CDCl_3_) *δ* 7.86 (d, *J* = 2.2 Hz, 1H), 7.56 (s, 1H), 7.44–7.46 (m, 1H), 7.40–7.42 (m, 2H), 7.29–7.34 (m, 3H), 4.17 (t, *J* = 6.4 Hz, 2H), 2.92 (p, *J* = 6.9 Hz, 1H), 2.85 (t, *J* = 7.6 Hz, 2H), 2.72–2.77 (m, 2H), 2.51 (q, *J* = 7.6 Hz, 2H), 2.37 (m, 2H), 2.11 (m, 2H), 1.73–1.87 (m, 4H), 1.66 (m, 1H), 1.36 (s, 3H), 1.27 (s, 3H), 1.25 (d, *J* = 2.5 Hz, 3H), 1.24 (d, *J* = 2.5 Hz, 3H), 1.12 (t, *J* = 7.6 Hz, 3H); ^13^C NMR (150 MHz, CDCl_3_) *δ* 198.7, 177.5, 153.2, 146.7, 147.1, 140.1, 136.3, 132.8, 130.8, 130.1, 129.9, 126.8, 126.5, 125.2, 123.7, 123.1, 64.2, 46.9, 44.0, 38.1, 37.5, 37.3, 36.8, 33.7, 29.8, 28.4, 24.4, 23.9, 23.9, 23.8, 22.3, 18.3, 16.6, 15.1; HRESIMS: calcd for C_33_H_42_N_3_O_3_ [M + H]^+^ 528.3226, found 528.3228 (mass error ∆m = −0.0002 ppm).

*19-O-(1-(4-Ethylphenyl)-1H-1,2,3-triazol-4-yl)propyl-7-oxo-dehydroabietic acid* (**26**). Yield: 63.3%, yellow oil, ^1^H NMR (600 MHz, CDCl_3_) *δ* 7.87 (d, *J* = 2.1 Hz, 1H), 7.80 (s, 1H), 7.64 (d, *J* = 8.1 Hz, 2H), 7.41 (dd, *J* = 8.1, 2.1 Hz, 1H), 7.32 (d, *J* = 8.2 Hz, 2H), 7.30 (d, *J* = 8.2 Hz, 1H), 4.15 (t, *J* = 6.3 Hz, 2H), 2.92 (p, *J* = 6.9 Hz, 1H), 2.83 (t, *J* = 8.2 Hz, 2H), 2.75 (d, *J* = 13.4 Hz, 2H), 2.68–2.73 (m, 2H), 2.37 (m, 2H), 2.08 (m, 2H), 1.72–1.82 (m, 4H), 1.62–1.68 (m, 1H), 1.35 (s, 3H), 1.27 (s, 3H), 1.27 (t, *J* = 6.2 Hz, 3H), 1.25 (d, *J* = 2.3 Hz, 3H), 1.23 (d, *J* = 2.3 Hz, 3H); ^13^C NMR (150 MHz, CDCl_3_) *δ* 198.7, 177.4, 153.2, 147.4, 147.1, 145.0, 135.2, 132.8, 130.8, 129.1, 125.2, 123.7, 120.6, 120.6, 119.5, 64.2, 46.9, 44.1, 38.1, 37.5, 37.3, 36.7, 33.7, 28.6, 28.4, 23.9, 23.9, 23.8, 22.3, 18.3, 16.5, 15.6; HRESIMS: calcd for C_33_H_42_N_3_O_3_ [M + H]^+^ 528.3226, found 528.3228 (mass error ∆m = −0.0002 ppm).

*19-O-(1-(4-Isopropylphenyl)-1H-1,2,3-triazol-4-yl)propyl-7-oxo-dehydroabietic acid* (**27**). Yield: 43.3%, yellow oil, ^1^H NMR (600 MHz, CDCl_3_) *δ* 7.87 (d, *J* = 2.1 Hz, 1H), 7.80 (s, 1H), 7.65 (d, *J* = 8.4 Hz, 2H), 7.41 (dd, *J* = 8.2, 2.2 Hz, 1H), 7.36 (d, *J* = 8.4 Hz, 2H), 7.30 (d, *J* = 8.2 Hz, 1H), 4.15 (t, *J* = 6.3 Hz, 2H), 2.98 (p, *J* = 6.9 Hz, 1H), 2.92 (p, *J* = 6.9 Hz, 1H), 2.83 (t, *J* = 7.5 Hz, 2H), 2.75 (d, *J* = 13.3 Hz, 2H), 2.37 (m, 2H), 2.09 (m, 2H), 1.72–1.83 (m, 4H), 1.63 (m, 1H), 1.35 (s, 3H), 1.29 (s, 3H), 1.28 (s, 3H), 1.27 (s, 3H), 1.25 (d, *J* = 2.7 Hz, 3H), 1.24 (d, *J* = 2.7 Hz, 3H); ^13^C NMR (150 MHz, CDCl_3_) *δ* 198.7, 177.5, 153.2, 149.7, 147.4, 147.1, 135.2, 132.8, 130.8, 127.8, 125.2, 123.8, 120.6, 120.6, 119.5, 64.2, 46.9, 44.1, 38.2, 37.5, 37.3, 36.7, 34.0, 33.7, 28.4, 24.1, 24.1, 23.9, 23.9, 23.8, 22.3, 18.3, 16.5; HRESIMS: calcd for C_34_H_44_N_3_O_3_ [M + H]^+^ 542.3383, found 542.3387 (mass error ∆m = −0.0004 ppm).

*18-O-(1-(3,5-Dimethoxyphenyl)-1H-1,2,3-triazol-4-yl)propyl-7-oxo-dehydroabietic acid* (**28**). Yield: 60.9%, yellow oil, ^1^H NMR (600 MHz, CDCl_3_) *δ* 7.86 (d, *J* = 2.2 Hz, 1H), 7.84 (s, 1H), 7.41 (dd, *J* = 8.2, 2.2 Hz, 1H), 7.30 (d, *J* = 8.2 Hz, 1H), 6.93 (d, *J* = 2.2 Hz, 2H), 6.49 (t, *J* = 2.2 Hz, 1H), 4.14 (t, *J* = 6.3 Hz, 2H), 3.86 (s, 6H), 2.92 (p, *J* = 6.9 Hz, 1H), 2.82 (td, *J* = 7.3, 2.2 Hz, 2H), 2.74 (d, *J* = 13.1 Hz, 2H), 2.36 (m, 2H), 2.09 (m, 2H), 1.74–1.82 (m, 4H), 1.64 (m, 1H), 1.35 (s, 3H), 1.27 (s, 3H), 1.24 (d, *J* = 2.7 Hz, 3H), 1.23 (d, *J* = 2.7 Hz, 3H); ^13^C NMR (150 MHz, CDCl_3_) *δ* 198.5, 177.2, 161.4, 153.0, 147.2, 146.9, 138.6, 132.6, 130.5, 124.9, 123.5, 119.4, 100.4, 98.7, 63.9, 55.6, 46.7, 43.9, 37.9, 37.3, 37.0, 36.4, 33.5, 28.2, 23.7, 23.7, 23.6, 22.1, 18.1, 16.3; HRESIMS: calcd for C_33_H_41_N_3_O_5_ [M + H]^+^ 560.3124, found 560.3127 (mass error ∆m = −0.0003 ppm).

*19-O-(1-(3,4,5-Trimethoxyphenyl)-1H-1,2,3-triazol-4-yl)propyl-7-oxo-dehydroabietic acid* (**29**). Yield: 67.3%, yellow oil, ^1^H NMR (600 MHz, CDCl_3_) *δ* 7.88 (s, 1H), 7.84 (d, *J* = 2.0 Hz, 1H), 7.41 (dd, *J* = 8.2, 2.0 Hz, 1H), 7.30 (d, *J* = 8.2 Hz, 1H), 7.02 (s, 2H), 4.13 (t, *J* = 6.2 Hz, 2H), 3.94 (s, 6H), 3.88 (s, 3H), 2.91 (p, *J* = 6.9 Hz, 1H), 2.82 (td, *J* = 7.3, 4.7 Hz, 2H), 2.70–2.77 (m, 2H), 2.36 (m, 2H), 2.08 (m, 2H), 1.78–1.87 (m, 4H), 1.59–1.68 (m, 1H), 1.34 (s, 3H), 1.27 (s, 3H), 1.23 (d, *J* = 3.0 Hz, 3H), 1.22 (d, *J* = 3.0 Hz, 3H); ^13^C NMR (150 MHz, CDCl_3_) *δ* 198.7, 177.4, 154.0, 153.2, 147.4, 147.1, 138.1, 133.2, 132.9, 130.6, 125.0, 123.8, 119.9, 98.4, 98.4, 64.1, 61.2, 56.5, 46.9, 44.2, 38.1, 37.6, 37.2, 36.6, 33.7, 28.4, 23.9, 23.8, 22.2, 18.3, 16.5; HRESIMS: calcd for C_34_H_43_N_3_O_6_ [M + H]^+^ 590.3230, found 590.3233 (mass error ∆m = −0.0003 ppm).

*18-O-(1-(2,3,4-Trimethoxyphenyl)-1H-1,2,3-triazol-4-yl)propyl-7-oxo-dehydroabietic acid* (**30**). Yield: 67.0%, yellow oil, ^1^H NMR (600 MHz, CDCl_3_) *δ* 7.85 (d, *J* = 2.1 Hz, 1H), 7.79 (s, 1H), 7.41 (dd, *J* = 8.1, 2.1 Hz, 1H), 7.38 (d, *J* = 8.9 Hz, 1H), 7.30 (d, *J* = 8.1 Hz, 1H), 6.77 (d, *J* = 8.9 Hz, 1H), 4.15 (h, *J* = 4.8 Hz, 2H), 3.92 (s, 3H), 3.92 (s, 3H), 3.76 (s, 3H), 2.92 (hept, *J* = 7.0 Hz, 1H), 2.83 (t, *J* = 7.6 Hz, 2H), 2.68–2.77 (m, 2H), 2.37 (m, 2H), 2.09 (m, 2H), 1.72–1.85 (m, 4H), 1.65 (m, 1H), 1.35 (s, 3H), 1.26 (s, 3H), 1.24 (d, *J* = 2.0 Hz, 3H), 1.23 (d, *J* = 2.0 Hz, 3H); ^13^C NMR (150 MHz, CDCl_3_) *δ* 198.6, 177.4, 154.4, 153.2, 147.1, 146.8, 146.5, 142.8, 132.8, 130.8, 125.2, 124.8, 123.7, 123.1, 120.1, 107.3, 64.2, 61.7, 61.3, 56.4, 46.9, 44.0, 38.1, 37.5, 37.2, 36.8, 33.7, 28.4, 23.9, 23.9, 23.9, 22.3, 18.3, 16.5; HRESIMS: calcd for C_34_H_43_N_3_O_6_ [M + H]^+^ 590.3230, found 590.3232 (mass error ∆m = −0.0002 ppm).

*19-O-(1-(5-Chloro-2-methoxyphenyl)-1H-1,2,3-triazol-4-yl)propyl-7-oxo-dehydroabietic acid* (**31**). Yield: 68.8%, yellow oil, ^1^H NMR (600 MHz, CDCl_3_) *δ* 7.95 (s, 1H), 7.85 (d, *J* = 2.2 Hz, 1H), 7.83 (d, *J* = 2.6 Hz, 1H), 7.41 (dd, *J* = 8.1, 2.2 Hz, 1H), 7.35 (dd, *J* = 8.8, 2.6 Hz, 1H), 7.30 (d, *J* = 8.1 Hz, 1H), 7.01 (d, *J* = 8.8 Hz, 1H), 4.15 (q, *J* = 6.0 Hz, 2H), 3.90 (s, 3H), 2.92 (hept, *J* = 7.0 Hz, 1H), 2.83 (t, *J* = 7.6 Hz, 2H), 2.68–2.77 (m, 2H), 2.36 (m, 2H), 2.09 (m, 2H), 1.69–1.85 (m, 4H), 1.60–1.68 (m, 1H), 1.35 (s, 3H), 1.26 (s, 3H), 1.24 (d, *J* = 2.0 Hz, 3H), 1.23 (d, *J* = 2.0 Hz, 3H); ^13^C NMR (150 MHz, CDCl_3_) *δ* 198.7, 177.5, 153.2, 149.7, 147.1, 146.6, 132.8, 130.8, 129.6, 127.2, 126.3, 125.3, 125.2, 123.7, 123.3, 113.5, 64.2, 56.5, 46.9, 44.0, 38.0, 37.5, 37.2, 36.7, 33.7, 28.3, 23.9, 23.9, 23.9, 22.3, 18.3, 16.5; HRESIMS: calcd for C_32_H_38_ClN_3_O_4_ [M + H]^+^ 564.2629, found 564.2631 (mass error ∆m = −0.0002 ppm).

*18-O-(1-(2-Chloro-4-methylphenyl)-1H-1,2,3-triazol-4-yl)propyl-7-oxo-dehydroabietic acid* (**32**). Yield: 64.8%, yellow oil, ^1^H NMR (600 MHz, CDCl_3_) *δ* 7.85 (d, *J* = 2.1 Hz, 1H), 7.75 (s, 1H), 7.47 (d, *J* = 8.1 Hz, 1H), 7.41 (dd, *J* = 8.1, 2.2 Hz, 1H), 7.37 (s, 1H), 7.30 (d, *J* = 8.1 Hz, 1H), 7.21 (d, *J* = 7.4 Hz, 1H), 4.16 (t, *J* = 6.2 Hz, 2H), 2.92 (hept, *J* = 6.8 Hz, 1H), 2.84 (t, *J* = 7.6 Hz, 2H), 2.71–2.75 (m, 2H), 2.42 (s, 3H), 2.37 (m, 2H), 2.09 (m, 2H), 1.71–1.83 (m, 4H), 1.65 (m, 1H), 1.35 (s, 3H), 1.26 (s, 3H), 1.24 (d, *J* = 2.2 Hz, 3H), 1.23 (d, *J* = 2.3 Hz, 3H); ^13^C NMR (150 MHz, CDCl_3_) *δ* 198.5,177.2, 152.9, 146.9, 146.3, 141.2, 132.6, 132.5, 130.9, 130.6, 128.4, 128.1, 127.3, 125.0, 123.5, 123.1, 64.0, 46.7, 43.8, 37.9, 37.3, 37.0, 36.5, 33.5, 28.1, 23.7, 23.7, 23.6, 22.1, 20.9, 18.1, 16.3; HRESIMS: calcd for C_32_H_38_ClN_3_O_3_ [M + H]^+^ 548.2680, found 548.2686 (mass error ∆m = −0.0006 ppm).

*18-O-(1-(4-(Trifluoromethoxy)phenyl)-1H-1,2,3-triazol-4-yl)propyl-7-oxo-dehydroabi etic acid* (**33**). Yield: 53.7%, yellow oil, ^1^H NMR (600 MHz, CDCl_3_) *δ* 7.90 (s, 1H), 7.87 (d, *J* = 2.0 Hz, 1H), 7.83 (s, 1H), 7.82 (s, 1H), 7.42 (dd, *J* = 8.1, 2.0 Hz, 1H), 7.39 (s, 1H), 7.38 (s, 1H), 7.31 (d, *J* = 8.1 Hz, 1H), 4.14 (q, *J* = 5.9 Hz, 2H), 2.92 (p, *J* = 6.9 Hz, 1H), 2.83 (q, *J* = 6.8 Hz, 2H), 2.76 (d, *J* = 13.4 Hz, 2H), 2.36 (m, 2H), 2.10 (m, 2H), 1.72–1.86 (m, 4H), 1.62–1.67 (m, 1H), 1.35 (s, 3H), 1.28 (s, 3H), 1.25 (d, *J* = 2.7 Hz, 3H), 1.24 (d, *J* = 2.7 Hz, 3H); ^13^C NMR (150 MHz, CDCl_3_) *δ* 198.8, 177.5, 153.2, 149.0, 147.9, 147.2, 135.7, 132.9, 130.7, 125.2, 123.8, 122.4, 121.9, 120.5 (q, *J*_CF_ = 258.4 Hz), 119.7, 64.0, 47.0, 44.1, 38.2, 37.6, 37.2, 36.7, 33.7, 29.8, 28.3, 23.9, 23.9, 23.8, 22.2, 18.3, 16.5; HRESIMS: calcd for C_32_H_36_F_3_N_3_O_4_ [M + H]^+^ 584.2736, found 584.2738 (mass error ∆m = −0.0002 ppm).

*18-O-(1-(3-(Trifluoromethoxy)phenyl)-1H-1,2,3-triazol-4-yl)propyl-7-oxo-dehydroabi etic acid* (**34**). Yield: 54.7%, yellow oil, ^1^H NMR (600 MHz, CDCl_3_) *δ* 7.93 (s, 1H), 7.87 (d, *J* = 1.9 Hz, 1H), 7.73 (d, *J* = 8.1 Hz, 2H), 7.56 (t, *J* = 8.3 Hz, 1H), 7.42 (dd, *J* = 8.1, 1.9 Hz, 1H), 7.31 (d, *J* = 8.1 Hz, 2H), 4.15 (t, *J* = 6.3 Hz, 2H), 2.92 (p, *J* = 6.9 Hz, 1H), 2.83 (t, *J* = 7.5 Hz, 2H), 2.76 (d, *J* = 15.1 Hz, 2H), 2.37 (m, 2H), 2.10 (m, 2H), 1.72–1.85 (m, 4H), 1.63–1.65 (m, 1H), 1.35 (s, 3H), 1.28 (s, 3H), 1.25 (d, *J* = 2.4 Hz, 3H), 1.24 (d, *J* = 2.4 Hz, 3H); ^13^C NMR (150 MHz, CDCl_3_) *δ* 198.7, 177.5, 153.2, 150.1, 148.0, 147.2, 138.4, 132.9, 131.2, 130.7, 125.2, 123.8, 120.7, 120.5 (q, *J*_CF_ = 258.6 Hz), 119.5, 118.4, 113.5, 64.1, 47.0, 44.2, 38.2, 37.6, 37.3, 36.7, 33.7, 28.3, 23.9, 23.9, 23.8, 22.3, 18.3, 16.5; HRESIMS: calcd for C_32_H_36_F_3_N_3_O_4_ [M + H]^+^ 584.2736, found 584.2735 (mass error ∆m = 0.0001 ppm).

*18-O-(1-(4-(Ethoxycarbonyl)phenyl)-1H-1,2,3-triazol-4-yl)propyl-7-oxo-dehydroabietic acid* (**35**). Yield: 65.5%, yellow oil, ^1^H NMR (600 MHz, CDCl_3_) *δ* 8.20 (d, *J* = 8.7 Hz, 2H), 7.96 (s, 1H), 7.87 (d, *J* = 8.2 Hz, 3H), 7.41 (dd, *J* = 8.1, 2.1 Hz, 1H), 7.30 (d, *J* = 8.1 Hz, 1H), 4.41 (q, *J* = 7.1 Hz, 2H), 4.14 (h, *J* = 4.8 Hz, 2H), 2.92 (hept, *J* = 6.9 Hz, 1H), 2.83 (td, *J* = 7.4, 3.8 Hz, 2H), 2.75 (d, *J* = 13.3 Hz, 2H), 2.36 (m, 2H), 2.09 (m, 2H), 1.70–1.86 (m, 4H), 1.60–1.66 (m, 1H), 1.42 (t, *J* = 7.2 Hz, 3H), 1.34 (s, 3H), 1.27 (s, 3H), 1.24 (d, *J* = 2.4 Hz, 3H), 1.23 (d, *J* = 2.4 Hz, 3H); ^13^C NMR (150 MHz, CDCl_3_) *δ* 198.7, 177.4, 165.6, 153.2, 147.9, 147.1, 140.3, 132.9, 131.4, 130.7, 130.4, 125.1, 123.8, 119.8, 119.4, 64.0, 61.5, 46.9, 44.1, 38.2, 37.6, 37.3, 36.6, 33.7, 28.3, 23.9, 23.9, 23.8, 22.2, 18.3, 16.5, 14.4; HRESIMS: calcd for C_34_H_41_N_3_O_5_ [M + H]^+^ 572.3124, found 572.3128 (mass error ∆m = −0.0004 ppm).

*18-O-(1-(3-Chloro-4-hydroxyphenyl)-1H-1,2,3-triazol-4-yl)propyl-7-oxo-dehydroabietic acid* (**36**). Yield: 45.0%, red oil, ^1^H NMR (600 MHz, CDCl_3_) *δ* 7.87 (s, 1H), 7.80 (s, 1H), 7.67 (d, *J* = 7.7 Hz, 1H), 7.56 (d, *J* = 8.7 Hz, 1H), 7.42 (d, *J* = 8.2 Hz, 1H), 7.31 (d, *J* = 8.2 Hz, 1H), 7.16 (d, *J* = 8.7 Hz, 1H), 4.15 (t, *J* = 6.0 Hz, 2H), 2.93 (p, *J* = 6.9 Hz, 1H), 2.82 (m, 2H), 2.75 (m, 2H), 2.37 (dd, *J* = 13.5, 6.5 Hz, 2H), 2.09 (m, 2H), 1.80–1.83 (m, 3H), 1.74 (m, 1H), 1.67 (m, 1H), 1.35 (s, 3H), 1.28 (s, 3H), 1.25 (d, *J* = 1.9 Hz, 3H), 1.24 (d, *J* = 1.9 Hz, 3H); ^13^C NMR (150 MHz, CDCl_3_) *δ* 198.8, 177.5, 153.2, 151.9, 147.2, 133.8, 132.9, 130.7, 127.8, 125.2, 123.8, 121.8, 121.0, 120.9, 119.7, 117.1, 64.1, 56.2, 47.0, 44.1, 38.2, 37.6, 37.3, 36.7, 33.7, 28.3, 23.9, 23.8, 22.2, 18.3, 16.5; HRESIMS: calcd for C_32_H_38_ClN_3_O_3_ [M + H]^+^ 548.2680, found 548.2686 (mass error ∆m = −0.0006 ppm).

*18-O-(1-(4-Hydroxyphenyl)-1H-1,2,3-triazol-4-yl)propyl-7-oxo-dehydroabietic acid* (**37**). Yield: 60.6%, brown oil, ^1^H NMR (600 MHz, CDCl_3_) *δ* 7.86 (d, *J* = 2.0 Hz, 1H), 7.76 (s, 1H), 7.53 (d, *J* = 8.6 Hz, 2H), 7.41 (dd, *J* = 8.2, 2.0 Hz, 1H), 7.30 (d, *J* = 8.2 Hz, 1H), 7.04 (d, *J* = 8.6 Hz, 2H), 4.14 (t, *J* = 6.3 Hz, 2H), 2.91 (p, *J* = 6.9 Hz, 1H), 2.82 (t, *J* = 7.6 Hz, 2H), 2.75 (d, *J* = 13.7 Hz, 2H), 2.36 (m, 2H), 2.08 (m, 2H), 1.71–1.83 (m, 4H), 1.57–1.63 (m, 1H), 1.34 (s, 3H), 1.26 (s, 3H), 1.23 (d, *J* = 2.1 Hz, 3H), 1.22 (d, *J* = 2.1 Hz, 3H); ^13^C NMR (150 MHz, CDCl_3_) *δ* 199.3, 177.6, 157.6, 153.3, 147.1, 133.0, 130.6, 129.9, 125.2, 123.8, 122.4, 120.0, 116.6, 64.2, 60.6, 46.9, 44.0, 38.1, 37.5, 37.2, 36.7, 33.7, 28.3, 23.9, 23.9, 23.8, 22.2, 21.2, 18.3, 16.5; HRESIMS: calcd for C_31_H_37_N_3_O_4_ [M + H]^+^ 516.2862, found 516.2863 (mass error ∆m = −0.0001 ppm).

*18-O-(1-(3-Hydroxyphenyl)-1H-1,2,3-triazol-4-yl)propyl-7-oxo-dehydroabietic acid* (**38**). Yield: 64.5%, white oil, ^1^H NMR (600 MHz, CDCl_3_) *δ* 8.47 (s, 1H), 7.94 (s, 1H), 7.87 (d, *J* = 2.1 Hz, 1H), 7.41 (dd, *J* = 8.1, 2.1 Hz, 1H), 7.34 (t, *J* = 7.8 Hz, 1H), 7.30 (d, *J* = 8.1 Hz, 1H), 7.08 (d, *J* = 7.8 Hz, 1H), 6.96 (d, *J* = 8.1 Hz, 1H), 4.15 (t, *J* = 6.0 Hz, 2H), 2.91 (p, *J* = 6.9 Hz, 1H), 2.85 (t, *J* = 7.5 Hz, 2H), 2.75 (d, *J* = 15.0 Hz, 2H), 2.37 (m, 2H), 2.11 (m, 2H), 1.72–1.84 (m, 4H), 1.62 (m, 1H), 1.34 (s, 3H), 1.26 (s, 3H), 1.24 (d, *J* = 2.4 Hz, 3H), 1.23 (d, *J* = 2.4 Hz, 3H); ^13^C NMR (150 MHz, CDCl_3_) *δ* 199.0, 177.4, 171.4, 158.8, 153.2, 147.1, 137.8, 132.9, 130.7, 130.6, 125.2, 123.8, 119.6, 116.6, 109.8, 108.8, 64.0, 60.5, 46.9, 44.1, 38.2, 37.5, 37.2, 36.6, 33.7, 28.1, 23.9, 22.1, 18.3, 16.5; HRESIMS: calcd for C_31_H_37_N_3_O_4_ [M + H]^+^ 516.2862, found 516.2863 (mass error ∆m = −0.0001 ppm).

*18-O-(1-(3-(Dimethylamino)phenyl)-1H-1,2,3-triazol-4-yl)propyl-7-oxo-dehydroabietic acid* (**39**). Yield: 52.0%, white oil, ^1^H NMR (600 MHz, CDCl_3_) *δ* 7.86 (d, *J* = 1.9 Hz, 1H), 7.81 (s, 1H), 7.41 (dd, *J* = 8.1, 1.9 Hz, 1H), 7.31 (d, *J* = 3.7 Hz, 1H), 7.30 (d, *J* = 3.8 Hz, 1H), 7.11 (d, *J* = 11.0 Hz, 1H), 6.94 (d, *J* = 7.8 Hz, 1H), 6.73 (d, *J* = 8.1 Hz, 1H), 4.15 (t, *J* = 6.3 Hz, 2H), 3.02 (s, 6H), 2.92 (p, *J* = 7.0 Hz, 1H), 2.82 (t, *J* = 7.6 Hz, 2H), 2.75 (d, *J* = 13.4 Hz, 2H), 2.37 (m, 2H), 2.08 (m, 2H), 1.73–1.84 (m, 4H), 1.64 (m, 1H), 1.34 (s, 3H), 1.27 (s, 3H), 1.24 (d, *J* = 2.3 Hz, 3H), 1.23 (d, *J* = 2.3 Hz, 3H); ^13^C NMR (150 MHz, CDCl_3_) *δ* 198.5, 177.3, 153.1, 151.4, 147.2, 147.0, 138.2, 132.7, 130.6, 130.1, 125.1, 123.6, 119.6, 112.5, 108.3, 104.6, 64.1, 46.8, 43.9, 40.6, 38.0, 37.4, 37.1, 36.6, 33.6, 28.3, 23.8, 23.8, 23.7, 22.2, 18.2, 16.4; HRESIMS: calcd for C_33_H_42_N_4_O_3_ [M + H]^+^ 542.3335, found 543.3333 (mass error ∆m = 0.0002 ppm).

### 4.3. NO Inhibitory Assay

Mouse small glioma cells and the BV2 cell line (Cat #20220326) were sourced from Saibai Kang (Shanghai) Biotechnology Co., Ltd. BV2 cells were seeded in 96-well cell culture plates (1.5 × 10^5^ cells/well) and treated with serial dilutions of the compounds, with a maximum concentration of 50 μM in triplicate, followed by stimulation with 1 μg·mL^−1^ LPS (Sigma, St. Louis, MO, USA) for 18 h. NO production in the supernatant was assessed by Griess reagents (Reagent A and Reagent B, respectively, Sigma). The absorbance at 570 nm was measured with a microplate reader (Thermo, Waltham, MA, USA). NG-Methyl-L-arginine acetate salt (L-NMMA, Sigma), a well-known nitric oxide synthase (NOS) inhibitor, was used as a positive control [14]. The viability of BV2 cells was evaluated by the MTS assay simultaneously, to exclude the interference of the cytotoxicity of the test compounds. All tests were performed in triplicate, and the results are expressed as IC_50_ values.

## 5. Conclusions

In summary, 33 7-oxodehydroabietic acid–1,2,3-triazole derivatives (**8**–**39**) were synthesized via a click reaction between O-propargylated 7-oxodehydroabietic acid (**4**), O-pentynylated 7-oxodehydroabietic acid (**6**), and different substituted aromatic azides. All compounds were further evaluated for their anti-inflammatory effect (NO inhibitory activities) on BV2 cell lines. Compounds **10**–**12**, **14**–**17**, and **22** exhibited a good anti-inflammatory effect (NO inhibitory activities) on BV2 cell lines with IC_50_ values from 8.40 ± 0.98 µM to 15.82 ± 1.40 µM. In particular, compounds **10** (IC_50_ = 8.40 ± 0.98 µM), **15** (IC_50_ = 10.74 ± 2.67 µM), **16** (IC_50_ = 10.96 ± 1.85 µM), and **17** (IC_50_ = 9.76 ± 1.27 µM) were the most promising derivatives. Based on the SAR studies, we propose that derivatives containing electron donors, such as a methoxy group or methyl group, and electron acceptors, such as a fluorine atom or chlorine atom in the aromatic ring, especially when most of the group are in the ortho and para positions, tend to have a better anti-inflammatory activity.

## Figures and Tables

**Table 1:** IC_50_ values in µM of 7-oxodehydroabietic acid–1,2,3-triazole hybrids on the panel of BV2 cell lines.

Compound	IC_50_ (µM)	Compound	IC_50_ (µM)
**8**	44.64 ± 1.28	**25**	>50
**9**	>50	**26**	>50
**10**	8.40 ± 0.98	**27**	>50
**11**	15.11 ± 1.89	**28**	>50
**12**	11.43 ± 1.24	**29**	>50
**13**	>50	**30**	>50
**14**	11.45 ± 2.05	**31**	>50
**15**	10.74 ± 2.67	**32**	>50
**16**	10.96 ± 1.85	**33**	>50
**17**	9.76 ± 1.27	**34**	>50
**18**	34.33 ± 1.41	**35**	>50
**19**	>50	**36**	>50
**20**	>50	**37**	>50
**21**	>50	**38**	>50
**22**	15.82 ± 1.40	**39**	>50
**23**	>50		
**24**	>50	**L-NMMA ^a^**	42.36 ± 2.47

^a^ L-NMMA: NG-methyl-L-arginine acetate salt.

## Data Availability

Data are contained within the article and Supplementary Materials.

## Supplementary Materials

The following supporting information can be downloaded at: https://www.mdpi.com/article/10.3390/molecules30030750/s1, Figures S1–S34: ^1^H NMR spectrum and ^13^C NMR spectrum of compound **4, 6, 8-39**; Tables S1–S4: ^13^C chemical shifts for Compounds **4**, **6**, **8**–**39**.

## References

[1] Gao J., Chen M., Ren X.C., Zhou X.B., Shang Q., Lu W.Q., Luo P., Jiang Z.H. (2017). Synthesis and cardiomyocyte protection activity of crocetin diamide derivatives. Fitoterapia.

[2] González M.A. (2015). Aromatic abietane diterpenoids: Their biological activity and synthesis. Nat. Prod. Rep..

[3] González M.A. (2015). Aromatic abietane diterpenoids: Total syntheses and synthetic studies. Tetrahedron.

[4] González M.A. (2014). Synthetic derivatives of aromatic abietane diterpenoids and their biological activities. Eur. J. Med. Chem..

[5] Fonseca T., Gigante B., Marques M.M., Gilchrist T.L., De Clercq E. (2004). Synthesis and antiviral evaluation of benzimidazoles, quinoxalines and indoles from dehydroabietic acid. Bioorg. Med. Chem..

[6] Kang M.S., Hirai S., Goto T., Kuroyanagi K., Lee J.Y., Uemura T., Ezaki Y., Takahashi N., Kawada T. (2008). Dehydroabietic acid, a phytochemical, acts as ligand for PPARs inmacrophages and adipocytes to regulate inflammation. Biochem. Biophys. Res. Commun..

[7] Li F.Y., Huang L., Li Q., Wang X., Ma X.L., Jiang C.N., Zhou X.Q., Duan W.G., Lei F.H. (2019). Synthesis and antiproliferative evaluation of novel hybrids of dehydroabietic acid bearing 1,2,3-Triazole Moiety. Molecules.

[8] Wada H., Kodato S., Kawamori M., Morikawa T., Nakai H., Takeda M., Saito S., Onoda Y., Tamaki H. (1985). Antiulcer activity of dehydroabietic acid derivatives. Chem. Pharm. Bull..

[9] Feio S.S., Roseiro J.C., Gigante B., Marcelo-Curto M.J. (1997). Method on multiwell plates for the evaluation of the antimicrobial activity of resin acid derivatives. J. Microbiol. Methods.

[10] Gigante B., Silva A.M., Marcelo-Curto M.J., Feio S.S., Roseiro J., Reis L.V. (2002). Structural effects on the bioactivity of dehydroabietic acid derivatives. Planta Med..

[11] De las Heras F.G., Alonso R., Alonso G. (1979). Synthesis and cytostatic activity of N-glycosyl(halomethyl)-1,2,3-triazoles. A new type of alkylating agent. J. Med. Chem..

[12] Agalave S.G., Maujan S.R., Pore V.S. (2011). Click Chemistry: 1,2,3-Triazoles as Pharmacophores. Chem. Asian J..

[13] Pingaew R., Mandi P., Nantasenamat C., Prachayasittikul S., Ruchirawat S., Prachayasittikul V. (2014). Design, synthesis and molecular docking studies of novel N-benzenesulfonyl-1,2,3,4-tetrahydroisoquinoline-based triazoles with potential anticancer activity. Eur. J. Med. Chem..

[14] Reif D.W., McCreedy S.A. (1995). N-Nitro-L-arginine and N-monomethyl-L-arginine exhibit a different pattern of inactivation toward the three nitric oxide synthases. Arch. Biochem. Biophys..

[15] Müller E., Kerschbaum H.H. (2006). Progesterone and its metabolites 5-dihydroprogesterone and 5-3-tetrahydroprogesterone decrease LPS-induced NO release in the murine microglial cell line, BV-2. Neuroendocrinol. Lett..

[16] Wang X., Su L., Liu S., He Z., Li J., Zong Y., Chen W., Du R. (2024). Paeoniflorin Inhibits the Activation of Microglia and Alleviates Depressive Behavior by Regulating SIRT1-NF-kB-NLRP3/Pyroptosis Pathway. Int. J. Mol. Sci..

[17] Lv X.S., Cui Y.M., Wang H.Y., Lin H.X., Ni W.Y., Ohwada T., Ido K., Sawada K. (2013). Synthesis and BK channel-opening activity of novel N-acylhydrazone derivatives from dehydroabietic acid. Chin. Chem. Lett..

[18] Rostovtsev V.V., Green L.G., Fokin V.V., Sharpless K.B. (2002). A stepwise Huisgen cycloaddition process: Copper(I)-catalyzed regioselective “ligation” of azides and terminal alkynes. Angew. Chem. Int. Ed..

[19] Farooq S., Shakeel U.R., Hussain A., Hamid A. (2014). Click chemistry inspired synthesis and bioevaluation of novel triazolyl derivatives of osthol as potent cytotoxic agents. Eur. J. Med. Chem..

[20] Clerc A., Bénéteau V., Pale P., Chassaing S. (2020). Chan-Lam-type azidation and one-pot CuAAC under CuI-Zeolite catalysis. ChemCatChem.

[21] Tao C.Z., Xin C., Li J., Liu A.X., Liu L., Guo Q.X. (2007). Copper-catalyzed synthesis of aryl azides and 1-aryl-1,2,3-triazoles from boronic acids. Tetrahedron Lett..

[22] Luo H., Lv Y.F., Zhang H., Hu J.M., Li H.M., Liu S.J. (2021). Synthesis and antitumor activity of 1-substituted 1,2,3-triazole-mollugin derivatives. Molecules.

[23] Lopez-Rojas P., Janeczko M., Kubinski K., Amesty A., Maslyk M., Estevez-Braun A. (2018). Synthesis and antimicrobial activity of 4-substituted 1,2,3-triazole-coumarin derivatives. Molecules.

[24] Silva V., Faria B.D., Colombo E., Ascari L., Freitas G.P.A., Flores L.S., Cordeiro Y., Ramao L., Buarque C.D. (2018). Design, synthesis, structural characterization and in vitro evaluation of new 1,4-disubstituted-1,2,3-triazole derivatives against glioblastoma cells. Bioorg. Chem..

