# Supplementary material for: Synthesis and Anti-Inflammatory Evaluation of Novel Hybrids of 7-Oxodehydroabietic Acid Bearing a 1,2,3-Triazole Moiety
Source: Molecules. 2025 Feb 6;30(3):750. doi: 10.3390/molecules30030750 (PMC11820262; doi:10.3390/molecules30030750)
Supplement: Supplementary file 1 [file molecules-30-00750-s001.zip › molecules-3430286-supplementary.pdf]

Supporting Information

Wen-Tao Fang [1,†], Yong-Feng Lv [2,3,†], Fu-Cai Ren [4], Hong Zhang [1], Dong-Mei Xie [1], Xiao-Bo Zhang [5], Cheng-Wu Fang [1,*], Shou-Jin Liu [1,*] and Han Luo [1,*]

[1] College of Pharmacy, Anhui University of Chinese Medicine, Hefei 230011, China; fangwentao@ahtcm.edu.cn (W.-T.F.); zhanghong@ahtcm.edu.cn (H.Z.); xiedongmei96@126.com (D.-M.X.)
[2] College of Science, Westlake University, Hangzhou 310024, China; lyuyongfeng@westlake.edu.cn
[3] State Key Laboratory of Phytochemistry and Plant Resources in West China, Kunming Institute of Botany, Chinese Academy of Sciences, Kunming 650201, China
[4] College of Pharmacy, Anhui Medical University, Hefei 230032, China; renfucai@ahmu.edu.cn
[5] State Key Laboratory for Quality Ensurance and Sustainable Use of Dao-di Herbs, Beijng 100700, China; jack110007@163.com
* Correspondence: cwfang1961@sina.com (C.-W.F.); shjinliu@sina.com (S.-J.L.); luohan@ahtcm.edu.cn (H.L.); Tel.: +86-133-9951-0966 (C.-W.F.); +86-139-5699-8502 (S.-J.L.); +86-152-5511-0598 (H.L.)
† These authors contributed equally to this work.

List of supplementary material Pages

**Table S1**	^{13}C chemical shifts for Compounds **4**, **6**, **8 - 15**	III
**Table S2**	^{13}C chemical shifts for Compounds **16 - 25**	IV
**Table S3**	^{13}C chemical shifts for Compounds **26 - 32**	V
**Table S4**	^{13}C chemical shifts for Compounds **33 - 39**	VI
**Figure S1**	^{1}H NMR spectrum and ^{13}C NMR spectrum of compound **4**	1
**Figure S2**	^{1}H NMR spectrum and ^{13}C NMR spectrum of compound **6**	2
**Figure S3**	^{1}H NMR spectrum and ^{13}C NMR spectrum of compound **8**	3
**Figure S4**	^{1}H NMR spectrum and ^{13}C NMR spectrum of compound **9**	4
**Figure S5**	^{1}H NMR spectrum and ^{13}C NMR spectrum of compound **10**	5
**Figure S6**	^{1}H NMR spectrum and ^{13}C NMR spectrum of compound **11**	6
**Figure S7**	^{1}H NMR spectrum and ^{13}C NMR spectrum of compound **12**	7
**Figure S8**	^{1}H NMR spectrum and ^{13}C NMR spectrum of compound **13**	8
**Figure S9**	^{1}H NMR spectrum and ^{13}C NMR spectrum of compound **14**	9
**Figure S10**	^{1}H NMR spectrum and ^{13}C NMR spectrum of compound **15**	10
**Figure S11**	^{1}H NMR spectrum and ^{13}C NMR spectrum of compound **16**	11

**Figure S12**	^{1}H NMR spectrum and ^{13}C NMR spectrum of compound **17**	12
**Figure S13**	^{1}H NMR spectrum and ^{13}C NMR spectrum of compound **18**	13
**Figure S14**	^{1}H NMR spectrum and ^{13}C NMR spectrum of compound **19**	14
**Figure S15**	^{1}H NMR spectrum and ^{13}C NMR spectrum of compound **20**	15
**Figure S16**	^{1}H NMR spectrum and ^{13}C NMR spectrum of compound **21**	16
**Figure S17**	^{1}H NMR spectrum and ^{13}C NMR spectrum of compound **22**	17
**Figure S18**	^{1}H NMR spectrum and ^{13}C NMR spectrum of compound **23**	18
**Figure S19**	^{1}H NMR spectrum and ^{13}C NMR spectrum of compound **24**	19
**Figure S20**	^{1}H NMR spectrum and ^{13}C NMR spectrum of compound **25**	20
**Figure S21**	^{1}H NMR spectrum and ^{13}C NMR spectrum of compound **26**	21
**Figure S22**	^{1}H NMR spectrum and ^{13}C NMR spectrum of compound **27**	22
**Figure S23**	^{1}H NMR spectrum and ^{13}C NMR spectrum of compound **28**	23
**Figure S24**	^{1}H NMR spectrum and ^{13}C NMR spectrum of compound **29**	24
**Figure S25**	^{1}H NMR spectrum and ^{13}C NMR spectrum of compound **30**	25
**Figure S26**	^{1}H NMR spectrum and ^{13}C NMR spectrum of compound **31**	26
**Figure S27**	^{1}H NMR spectrum and ^{13}C NMR spectrum of compound **32**	27
**Figure S28**	^{1}H NMR spectrum and ^{13}C NMR spectrum of compound **33**	28
**Figure S29**	^{1}H NMR spectrum and ^{13}C NMR spectrum of compound **34**	29
**Figure S30**	^{1}H NMR spectrum and ^{13}C NMR spectrum of compound **35**	30
**Figure S31**	^{1}H NMR spectrum and ^{13}C NMR spectrum of compound **36**	31
**Figure S32**	^{1}H NMR spectrum and ^{13}C NMR spectrum of compound **37**	32
**Figure S33**	^{1}H NMR spectrum and ^{13}C NMR spectrum of compound **38**	33
**Figure S34**	^{1}H NMR spectrum and ^{13}C NMR spectrum of compound **39**	34

Table S1. ^{13}C chemical shifts for Compounds **4**, **6**, **8 - 15**; solvent: $CDCl_3$, recorded at 150 MHz

NO	**4**	**6**	**8**	**9**	**10**	**11**	**12**	**13**	**14**	**15**
**C**	δ_C	δ_C	δ_C	δ_C	δ_C	δ_C	δ_C	δ_C	δ_C	δ_C
1	37.6	37.9	37.8	37.8	37.8	37.8	37.7	37.7	37.8	37.8
2	18.0	18.2	18.0	18.0	18.0	18.1	18.1	18.1	18.1	18.1
3	37.2	37.4	37.2	37.3	37.2	37.3	37.2	37.2	37.3	37.3
4	43.7	43.9	43.6	43.6	43.6	43.7	43.5	43.6	43.6	43.7
5	46.6	46.8	46.6	46.6	46.6	46.7	46.5	46.6	46.6	46.6
6	36.2	36.7	36.3	36.3	36.4	36.4	36.4	36.4	36.5	36.4
7	198.0	198.6	198.2	198.3	198.2	198.3	198.3	198.2	198.4	198.3
8	132.5	132.7	132.5	132.5	132.5	132.6	132.5	132.5	132.6	132.6
9	152.8	153.1	152.8	152.8	152.8	152.9	152.8	152.8	152.9	152.9
10	37.0	37.2	37.0	36.9	37.0	37.0	36.9	37.0	37.0	37.0
11	123.5	123.6	123.4	123.4	123.4	123.5	123.4	123.4	123.5	123.5
12	130.6	130.8	130.6	130.5	130.6	130.7	130.6	130.6	130.7	130.7
13	146.8	147.0	146.9	146.8	146.9	147.0	146.9	146.9	147.0	147.0
14	124.9	125.2	125.0	124.9	125.0	125.0	125.0	125.0	125.0	125.1
15	33.5	33.7	33.5	33.5	33.5	33.6	33.5	33.5	33.6	33.6
16	23.8	23.9	23.8	23.7	23.7	23.8	23.8	23.7	23.8	23.8
17	23.7	23.9	23.7	23.7	23.7	23.8	23.7	23.7	23.8	23.8
18	176.3	177.3	177.2	177.1	177.2	177.3	177.2	177.1	177.2	177.2
19	16.3	16.5	16.3	16.3	16.3	16.4	16.3	16.3	16.4	16.4
20	23.7	23.8	23.6	23.6	23.6	23.7	23.7	23.6	23.7	23.7
1'	52.3	63.6	58.0	58.0	58.0	58.1	58.1	58.0	58.0	57.9
2'	77.6	27.5	142.5	143.2	142.5	142.5	141.9	142.6	142.4	142.5
3'	75.1	15.3	125.6	121.8	125.2	125.6	125.9	124.2	126.2	126.0
4'		82.9								
5'		69.3								
1''			132.6	134.7	136.0	127.4	104.7	107.2	113.3	132.2
2''			138.7	117.9	136.8	135.4	152.5	146.6	149.6	141.6
3''			136.3	138.3	131.2	116.4	99.5	142.4	125.3	128.6
4''			131.4	137.6	130.6	160.4	161.2	154.4	129.8	131.0
5''			126.1	121.7	130.2	111.9	119.7	120.0	126.7	127.5
6''			124.0	130.5	126.5	129.5	126.5	125.5	125.8	128.3
2''-$\underline{C}H_3$			14.2			18.0				
3''-$\underline{C}H_3$			20.3	19.8						
4''-$\underline{C}H_3$				19.4						21.1
2''-$\underline{C}H_2CH_3$					20.6					
2''-$CH_2\underline{C}H_3$					17.3					
2''-$O\underline{C}H_3$							55.9	61.4	56.4	
3''-$O\underline{C}H_3$								61.1		
4''-$O\underline{C}H_3$						55.6	55.6	56.2		

Table S2. ^{13}C chemical shifts for Compounds **16 - 25**; solvent: $CDCl_3$, recorded at 150 MHz

**NO**	**16**	**17**	**18**	**19**	**20**	**21**	**22**	**23**	**24**	**25**
**C**	δ_C	δ_C	δ_C	δ_C	δ_C	δ_C	δ_C	δ_C	δ_C	δ_C
1	37.7	37.9	37.8	37.9	38.0	38.0	37.9	38.1	38.1	38.1
2	18.0	18.0	18.0	18.0	18.0	18.0	18.1	18.3	18.4	18.3
3	37.2	37.3	37.2	37.3	37.4	37.3	37.4	37.5	37.6	37.5
4	43.6	43.7	43.5	43.7	43.8	43.7	43.8	44.0	44.1	44.0
5	46.6	46.6	46.5	46.6	46.6	46.6	46.7	46.9	46.9	46.9
6	36.3	36.3	36.4	36.3	36.3	36.3	36.4	36.7	36.7	36.8
7	198.3	198.4	198.3	198.4	198.4	198.4	199.3	198.6	198.7	198.7
8	132.5	132.6	132.5	132.6	132.7	132.6	133.0	132.9	132.8	132.8
9	152.8	152.8	152.8	152.8	152.8	152.8	153.1	153.2	153.2	153.2
10	36.9	37.0	36.9	37.0	37.0	37.0	37.0	37.2	37.3	37.3
11	123.4	123.5	123.4	123.5	123.6	123.5	123.7	123.7	123.7	123.7
12	130.6	130.5	130.6	130.5	130.5	130.5	130.4	130.8	130.8	130.8
13	146.9	146.9	146.9	146.9	146.9	146.9	147.0	147.1	147.1	147.1
14	125.0	125.0	125.0	124.9	124.9	124.9	125.1	125.2	125.2	125.2
15	33.5	33.5	33.5	33.5	33.5	33.5	33.6	33.7	33.7	33.7
16	23.8	23.7	23.7	23.7	23.7	23.7	23.8	23.9	23.9	23.9
17	23.7	23.7	23.7	23.7	23.7	23.7	23.7	23.9	23.9	23.9
18	177.1	177.2	177.2	177.2	177.1	177.1	177.3	177.4	177.5	177.5
19	16.3	16.3	16.3	16.3	16.3	16.3	16.4	16.5	16.5	16.6
20	23.6	23.5	23.6	23.5	23.5	23.5	23.6	23.8	23.8	23.8
1'	57.9	57.9	57.7	57.9	57.9	57.9	57.9	64.2	64.2	64.2
2'	–	–	142.7	143.6	143.8	143.9	143.0	28.4	28.4	28.4
3'	125.9	121.9	126.4	122.0	121.9	121.7	122.3	22.2	22.4	22.3
4'								146.6	–	146.7
5'								124.1	119.5	123.1
1''	110.7	132.3	115.0	133.1	135.2	139.8	129.5	132.8	135.3	140.1
2''	154.4	108.1	156.7	122.7	122.2	120.0	122.6	138.8	117.9	136.3
3''	102.5	161.2	112.5	116.6	122.1	131.2	116.5	136.9	138.4	130.1
4''	161.0	125.8	131.4	162.5	149.1	130.6	157.6	131.3	137.3	129.9
5''	110.7	135.6	112.5	116.6	122.1	131.2	116.5	126.2	130.7	126.8
6''	118.3	115.7	156.7	122.7	122.2	120.0	122.6	123.2	121.8	126.5
2''-$\underline{C}H_3$								14.3		
3''-$\underline{C}H_3$								20.5	20.0	
4''-$\underline{C}H_3$		14.3							19.6	
2''-$\underline{C}H_2CH_3$										24.4
2''-$CH_2\underline{C}H_3$										15.1
4''-$O\underline{C}H_3$	55.8									
4''-$O\underline{C}F_3$					120.3					
4''-$\underline{C}OOCH_2CH_3$						165.3				
4''-$COO\underline{C}H_2CH_3$						61.3				
4''-$COOCH_2\underline{C}H_3$						14.3				

Table S3. ^{13}C chemical shifts for Compounds **26 - 32**; solvent: $CDCl_3$, recorded at 150 MHz

**NO**	**26**	**27**	**28**	**29**	**30**	**31**	**32**
**C**	δ_C	δ_C	δ_C	δ_C	δ_C	δ_C	δ_C
1	38.1	38.2	37.9	38.1	38.1	38.0	37.9
2	18.3	18.3	18.1	18.3	18.3	18.3	18.1
3	37.5	37.5	37.3	37.6	37.5	37.5	37.3
4	44.1	44.1	43.9	44.2	44.0	44.0	43.8
5	46.9	46.9	46.7	46.9	46.9	46.9	46.7
6	36.7	36.7	36.4	36.6	36.8	36.7	36.5
7	198.7	198.7	198.5	198.7	198.6	198.7	198.5
8	132.8	132.8	132.6	132.9	132.8	132.8	132.6
9	153.2	153.2	153.0	153.2	153.2	153.2	152.9
10	37.3	37.3	37.0	37.2	37.2	37.2	37.0
11	123.7	123.8	123.5	123.8	123.7	123.7	123.5
12	130.8	130.8	130.5	130.6	130.8	130.8	130.6
13	147.1	147.1	146.9	147.1	147.1	147.1	146.9
14	125.2	125.2	124.9	125.0	125.2	125.2	125.0
15	33.7	33.7	33.5	33.7	33.7	33.7	33.5
16	23.9	23.9	23.7	23.9	23.9	23.9	23.7
17	23.9	23.9	23.7	23.9	23.9	23.9	23.7
18	177.4	177.5	177.2	177.4	177.4	177.5	177.2
19	16.5	16.5	16.3	16.5	16.5	16.5	16.3
20	23.8	23.8	23.6	23.8	23.9	23.8	23.6
1'	64.2	64.2	63.9	64.1	64.2	64.2	64.0
2'	28.6	28.4	28.2	28.4	28.4	28.3	28.1
3'	22.3	22.3	22.1	22.2	22.3	22.3	22.1
4'	147.4	147.4	147.2	147.4	146.5	146.6	146.3
5'	119.5	119.5	119.4	119.9	123.1	123.3	123.1
1''	135.2	135.2	138.6	138.1	124.8	127.2	132.5
2''	129.1	120.6	98.7	98.4	146.8	149.7	141.2
3''	120.6	127.8	161.4	154.0	142.8	113.5	128.4
4''	145.0	149.7	100.4	133.2	154.4	129.6	130.9
5''	120.6	127.8	161.4	154.0	107.3	126.3	127.3
6''	129.1	120.6	98.7	98.4	120.1	125.3	128.1
4''-<u>C</u>H₃							20.9
4''-<u>C</u>H₂CH₃	28.4						
4''-CH₂<u>C</u>H₃	15.6						
2''-O<u>C</u>H₃					61.3	56.5	
3''-O<u>C</u>H₃			55.6	56.5	61.7		
4''-O<u>C</u>H₃				61.2	56.4		
5''-O<u>C</u>H₃			55.6	56.5			
4''-<u>C</u>H(CH₃)₂		34.0					
4''-CH(<u>C</u>H₃)₂		24.1					

Table S4. ^{13}C chemical shifts for Compounds **33- 39**; solvent: $CDCl_3$, recorded at 150 MHz

**NO**	**33**	**34**	**35**	**36**	**37**	**38**	**39**
**C**	δ_C	δ_C	δ_C	δ_C	δ_C	δ_C	δ_C
1	38.2	38.2	38.2	38.2	38.1	38.2	38.0
2	18.3	18.3	18.3	18.4	18.3	18.3	18.2
3	37.6	37.6	37.6	37.6	37.5	37.6	37.4
4	44.1	44.2	44.1	44.2	44.0	44.1	43.9
5	47.0	47.0	46.9	47.0	46.9	46.9	46.8
6	36.7	36.7	36.6	36.7	36.7	36.7	36.6
7	198.8	198.7	198.7	198.8	199.3	199.0	198.5
8	132.9	132.9	132.9	132.9	133.0	132.9	132.7
9	153.2	153.2	153.2	153.2	153.3	153.2	153.1
10	37.3	37.3	37.3	37.3	37.2	37.2	37.1
11	123.8	123.8	123.8	123.8	123.8	123.8	123.6
12	130.7	130.7	130.7	130.7	130.6	130.6	130.6
13	147.2	147.2	147.1	147.2	147.1	147.1	147.0
14	125.2	125.2	125.1	125.2	125.2	125.2	125.1
15	33.8	33.7	33.7	33.8	33.7	33.7	33.6
16	23.9	23.9	23.9	23.9	23.9	23.9	23.8
17	23.9	23.9	23.9	23.9	23.9	23.9	23.8
18	177.5	177.5	177.4	177.5	177.6	177.4	177.3
19	16.5	16.5	16.5	16.5	16.5	16.5	16.4
20	23.8	23.8	23.8	23.8	23.8	23.8	23.7
1'	64.0	64.1	64.0	64.1	64.2	64.0	64.1
2'	28.3	28.3	28.3	28.4	28.3	28.1	28.3
3'	22.2	22.3	22.2	22.3	22.2	22.1	22.2
4'	147.9	148.0	147.9	–	–	147.2	147.2
5'	119.7	119.5	119.4	120.9	120.0	119.6	119.6
1''	135.7	138.4	140.3	133.8	129.9	137.7	138.2
2''	122.4	113.5	119.8	127.8	122.4	108.8	104.5
3''	121.9	150.1	131.4	121.8	116.6	158.8	151.4
4''	149.0	120.7	130.4	151.9	157.6	109.7	108.3
5''	121.9	131.2	131.4	121.0	116.6	130.7	130.1
6''	122.4	118.4	119.8	117.1	122.4	116.6	112.5
3''-O<u>C</u>F_3		120.5					
4''-O<u>C</u>F_3	120.5						
4''-<u>C</u>OOCH$_2$CH$_3$			165.6				
4''-COO<u>C</u>H$_2$CH$_3$			61.5				
4''-COOCH$_2$<u>C</u>H$_3$			14.4				
3''-N(<u>C</u>H$_3$)$_2$							40.6

O-propargylated 7-oxodehydroabietic acid (**4**) yellow oil, ^{1}H NMR (600 MHz, $CDCl_3$) δ 7.80 (d, J = 2.1 Hz, 1H), 7.34 (dd, J = 8.2, 2.1 Hz, 1H), 7.23 (d, J = 8.2 Hz, 1H), 4.57 (d, J = 2.7 Hz, 2H), 2.85 (hept, J = 6.9 Hz, 1H), 2.61 - 2.68 (m, 2H), 2.41 (t, J = 2.5 Hz, 1H), 2.26 - 2.37 (m, 2H), 1.72 - 1.77 (m, 3H), 1.67 (m, 1H), 1.53 - 1.60 (m, 1H), 1.29 (s, 3H), 1.18 (s, 3H), 1.17 (d, J = 1.8 Hz, 3H), 1.16 (d, J = 1.8 Hz, 3H); ^{13}C NMR (150 MHz, $CDCl_3$) δ 198.0, 176.3, 152.8, 146.8, 132.5, 130.6, 124.9, 123.5, 77.6,75.1, 52.3, 46.6, 43.7, 37.6, 37.2, 37.0, 36.2, 33.5, 23.8, 23.7, 23.7, 18.0, 16.3.

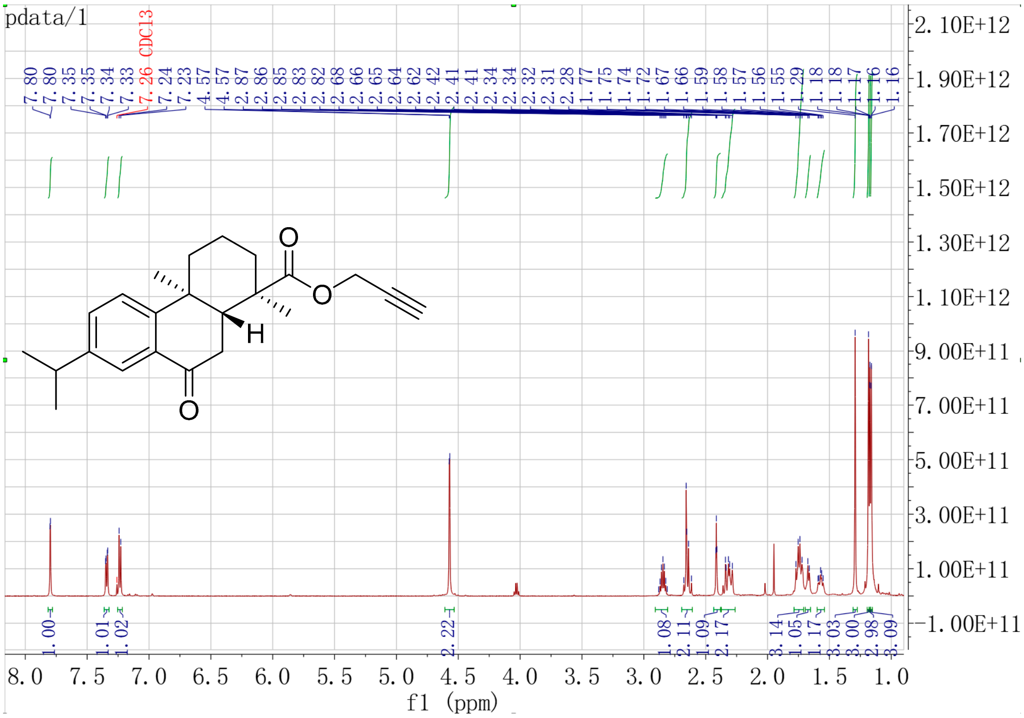

Figure S1-1. ^{1}H NMR spectrum of compound **4**

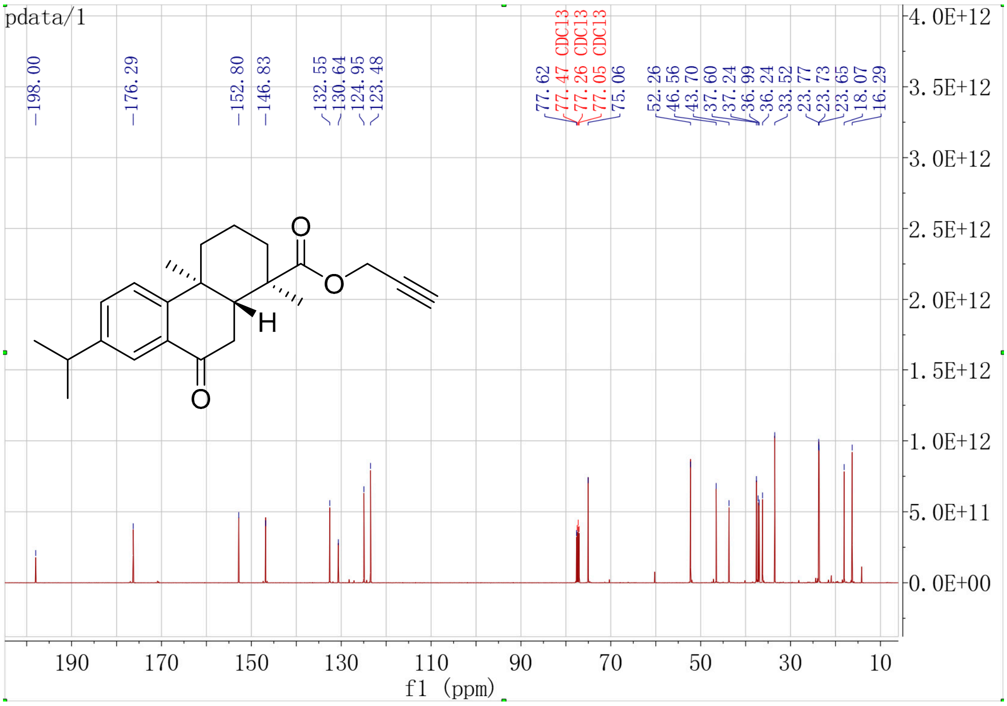

Figure S1-2. ^{13}C NMR spectrum of compound **4**

O-pentynylated 7-oxodehydroabietic acid (**6**) yellow oil, ^{1}H NMR (600 MHz, $CDCl_3$) δ 7.86 (d, J = 2.1 Hz, 1H), 7.40 (dd, J = 8.2, 2.2 Hz, 1H), 7.20 (d, J = 8.2 Hz, 1H), 4.15 (qt, J = 11.1, 6.3 Hz, 2H), 2.92 (hept, J = 6.9 Hz, 1H), 2.68 - 2.73 (m, 2H), 2.32 - 2.39 (m, 2H), 2.23 (td, J = 7.0, 2.6 Hz, 2H), 1.93 (t, J = 2.6 Hz, 1H), 1.76 - 1.88 (m, 4H), 1.71 - 1.73 (m, 2H), 1.61 - 1.66 (m, 1H), 1.33 (s, 3H), 1.25 (s, 3H), 1.24 (d, J = 1.6 Hz, 3H), 1.23 (d, J = 1.6 Hz, 3H); ^{13}C NMR (150 MHz, $CDCl_3$) δ 198.6, 177.3, 153.1, 147.0, 132.7, 130.8, 125.2, 123.6, 82.9, 68.3, 63.6, 46.8, 43.9, 37.9, 37.4, 37.2, 36.7, 33.7, 27.5, 23.9, 23.9, 23.8, 18.3, 16.5, 15.3.

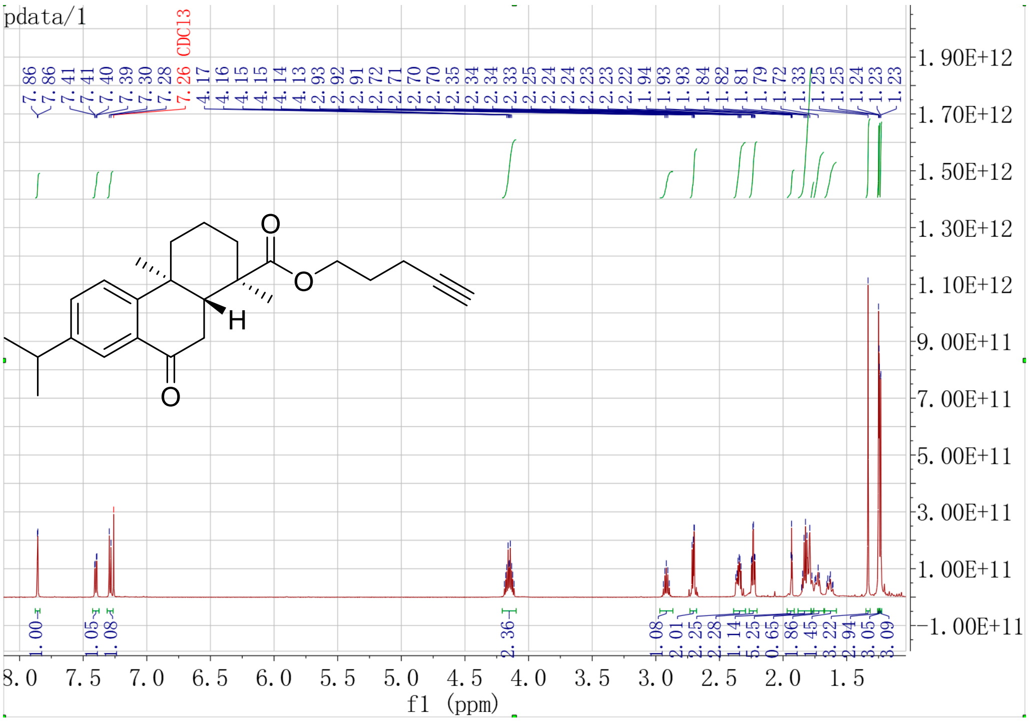

Figure S2-1. ^{1}H NMR spectrum of compound **6**

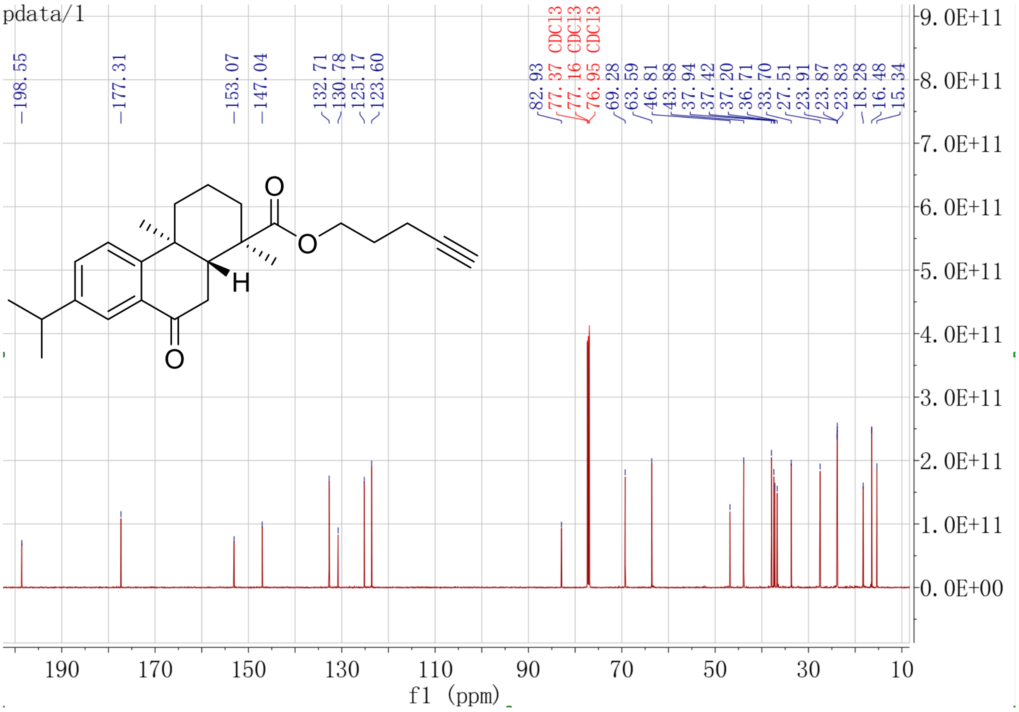

Figure S2-2. ^{13}C NMR spectrum of compound **6**

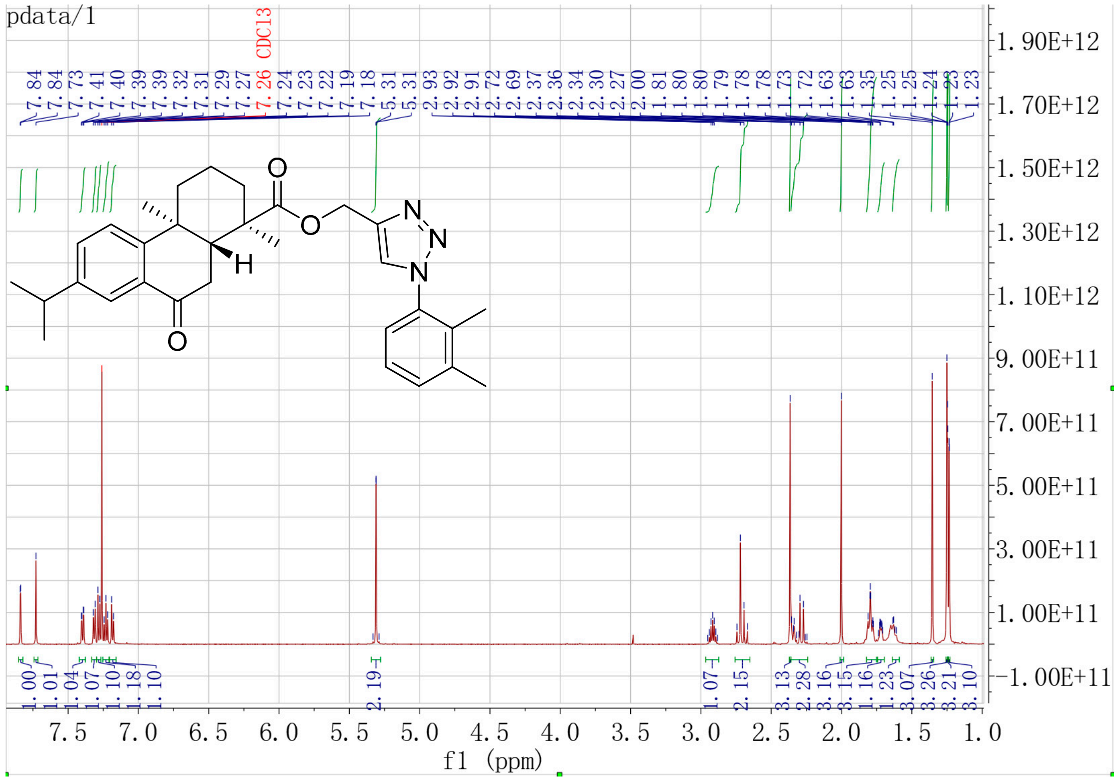

Figure S3-1. ^{1}H NMR spectrum of compound **8**

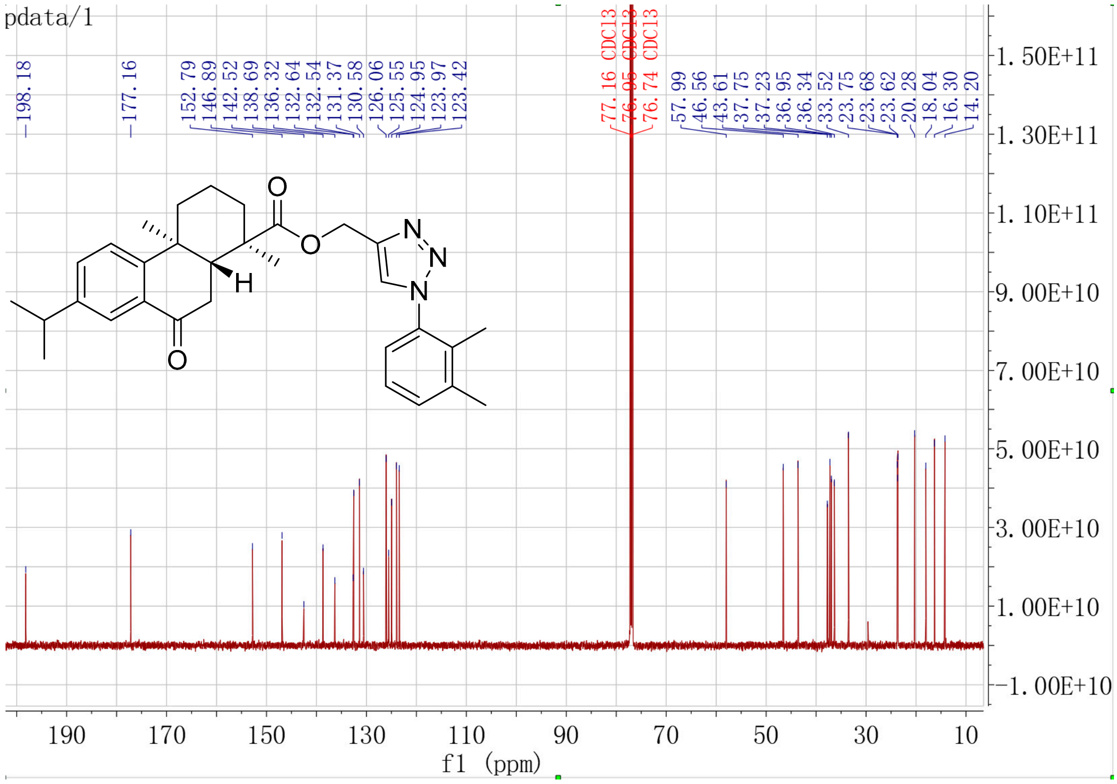

Figure S3-2. ^{13}C NMR spectrum of compound **8**

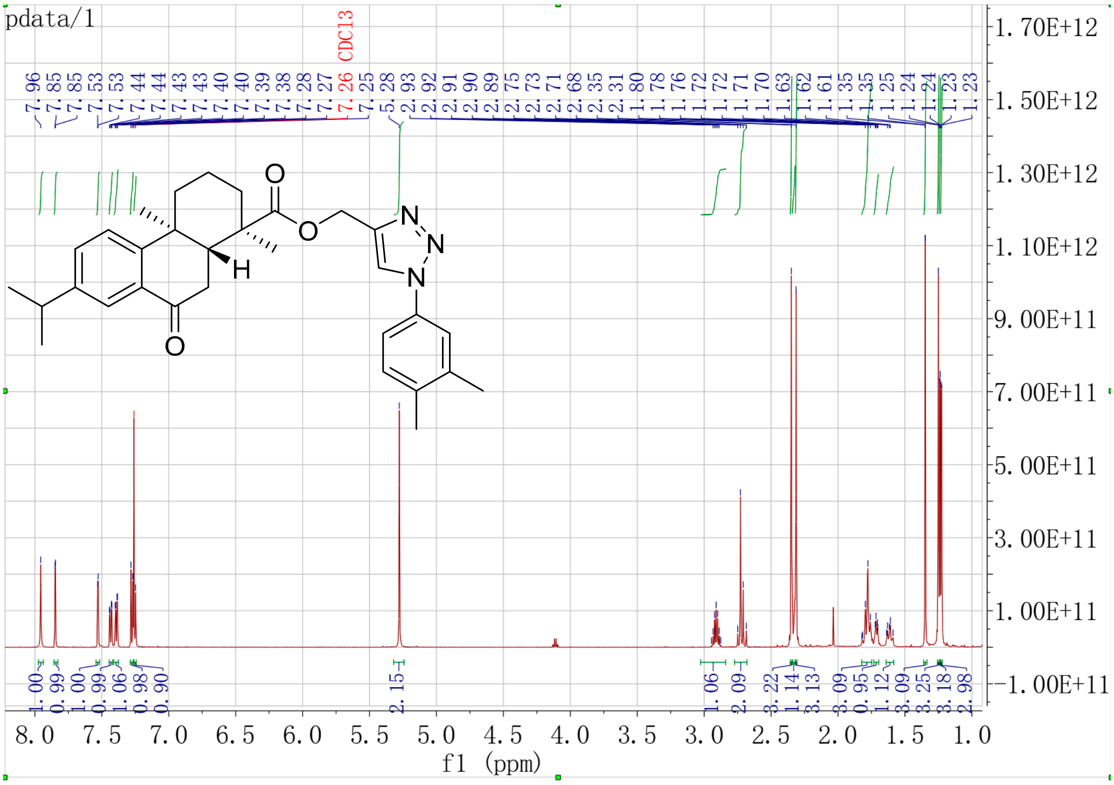

Figure S4-1. ^{1}H NMR spectrum of compound **9**

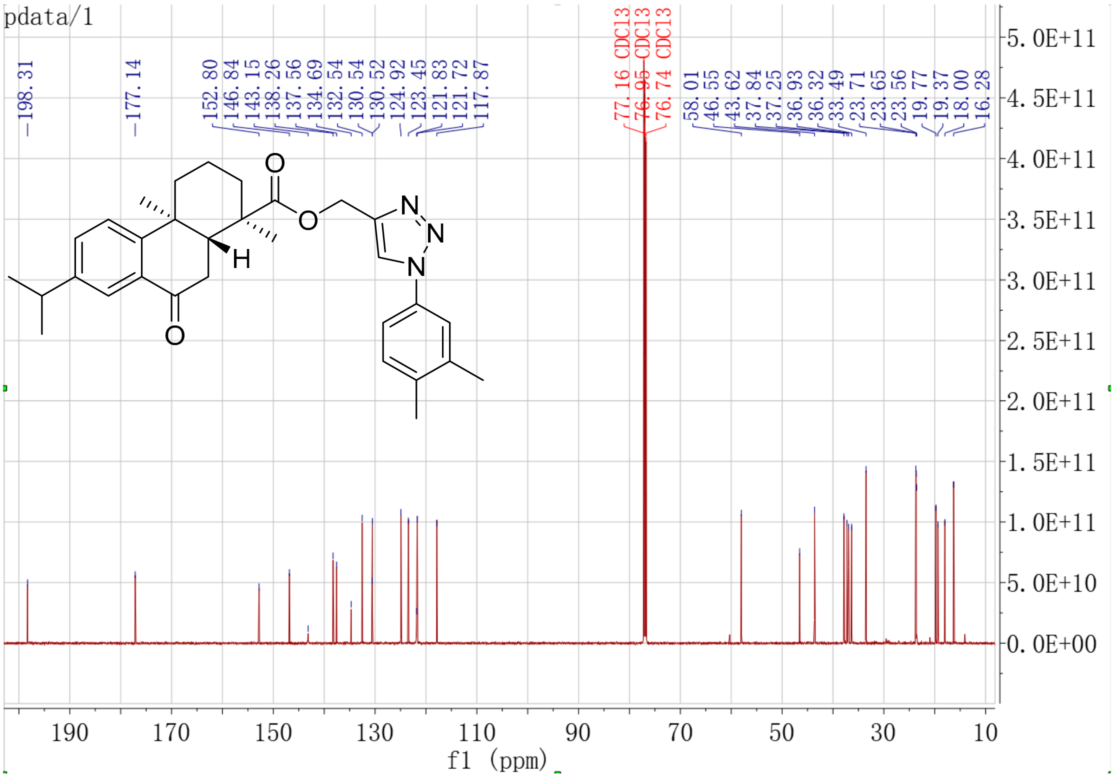

Figure S4-2. ^{13}C NMR spectrum of compound **9**

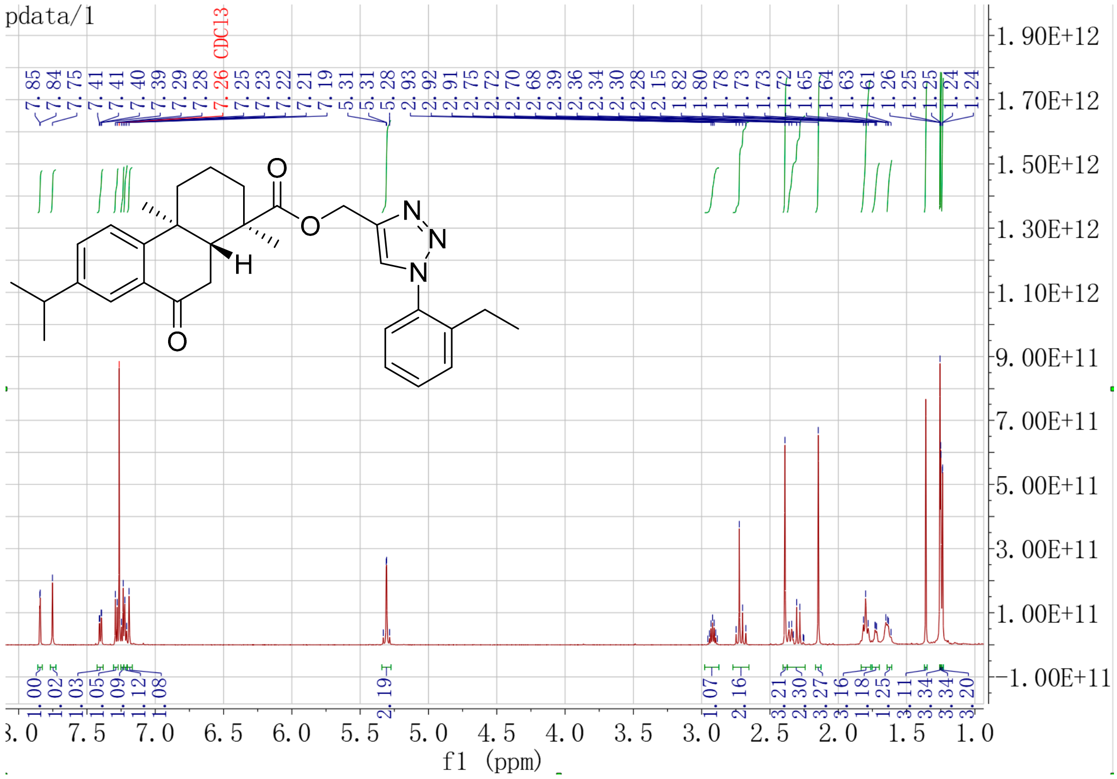

Figure S5-1. ^{1}H NMR spectrum of compound **10**

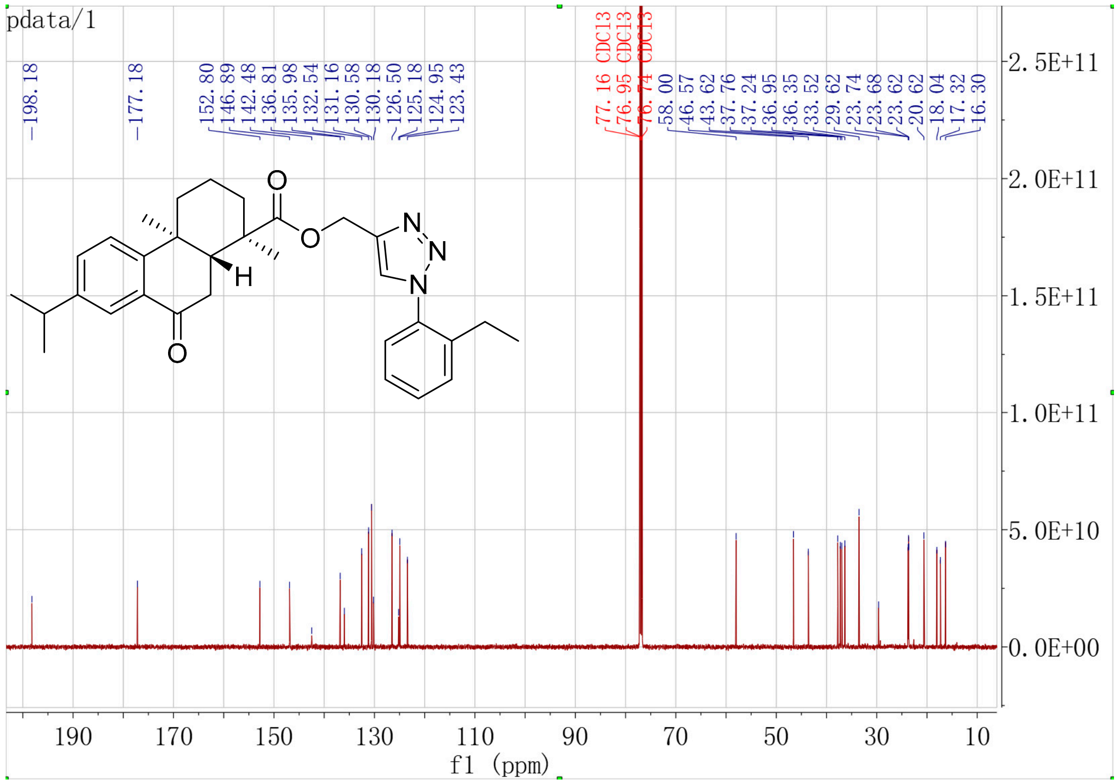

Figure S5-2. ^{13}C NMR spectrum of compound **10**

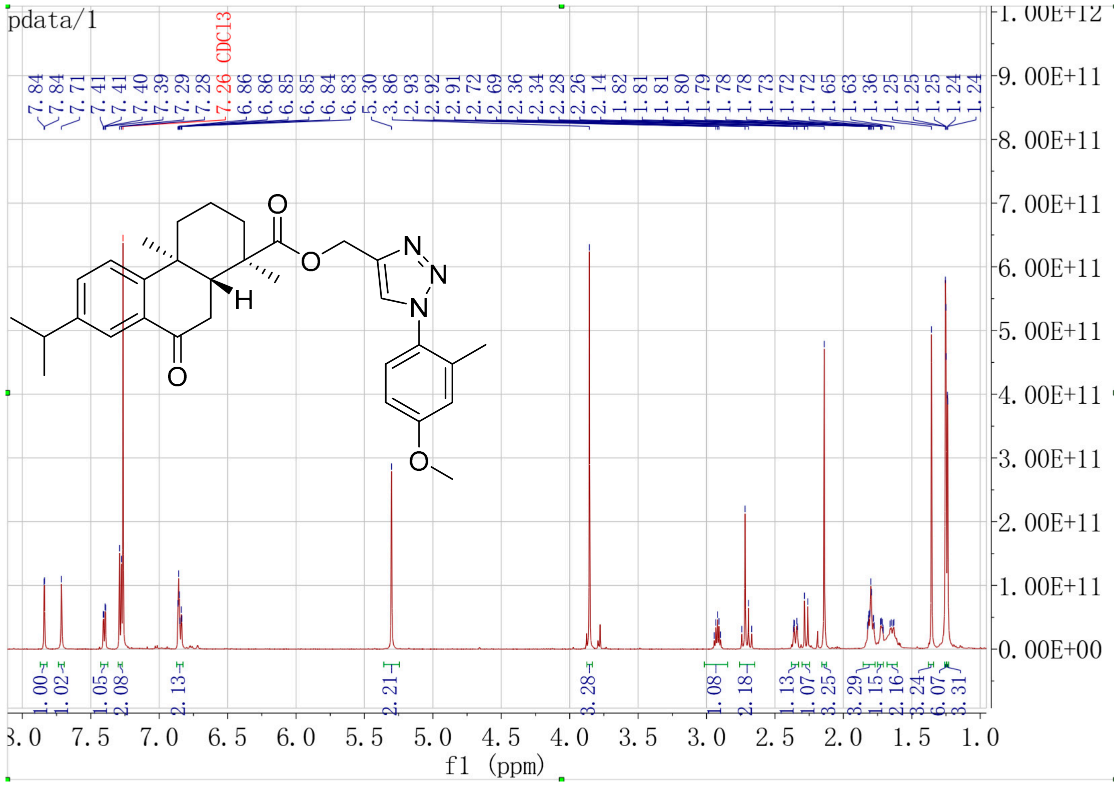

Figure S6-1. ^{1}H NMR spectrum of compound **11**

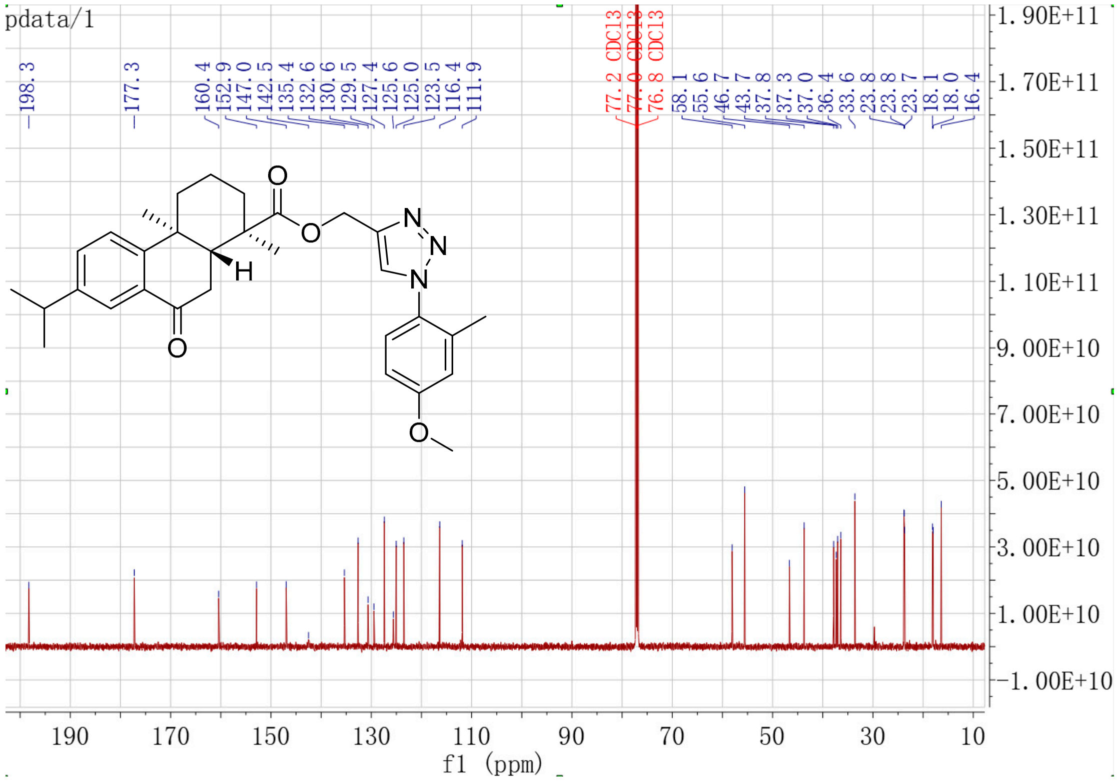

Figure S6-2. ^{13}C NMR spectrum of compound **11**

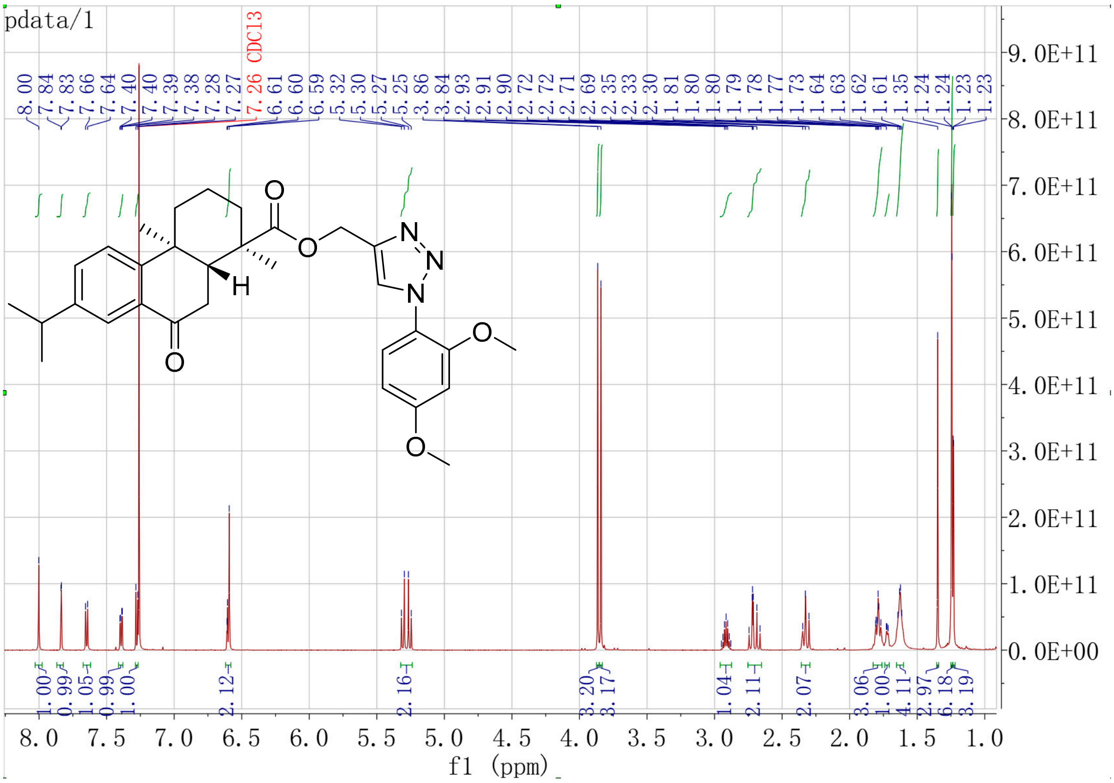

Figure S7-1. ^{1}H NMR spectrum of compound **12**

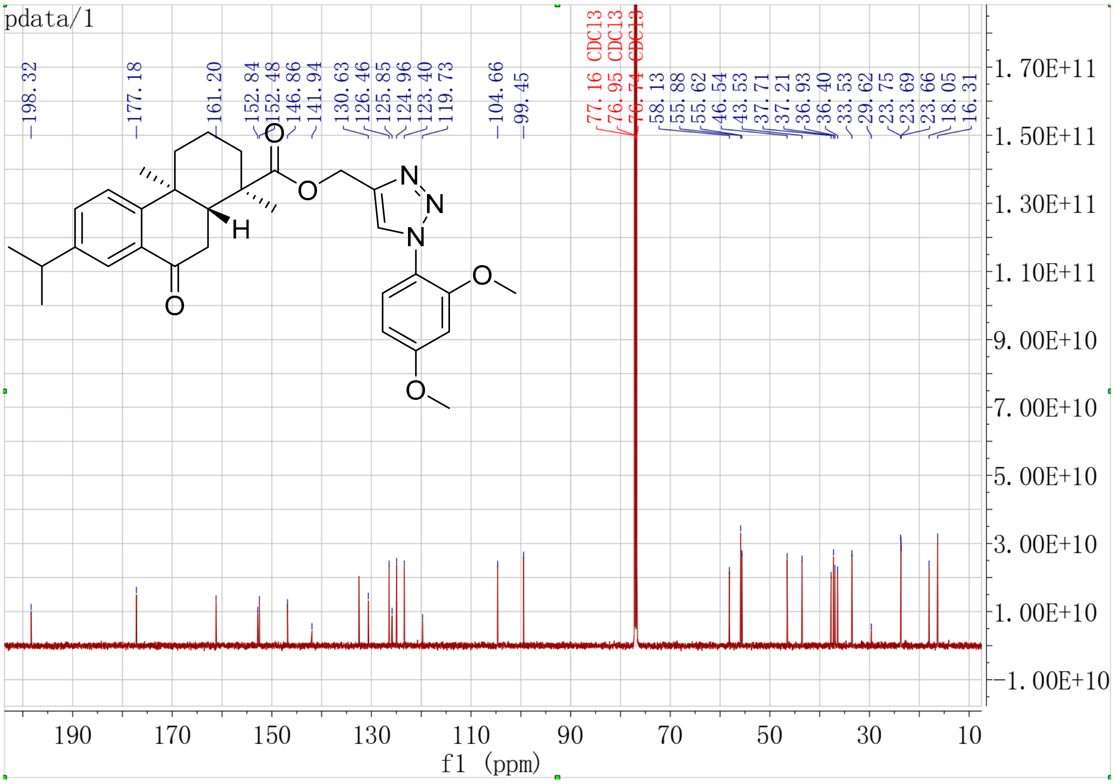

Figure S7-2. ^{13}C NMR spectrum of compound **12**

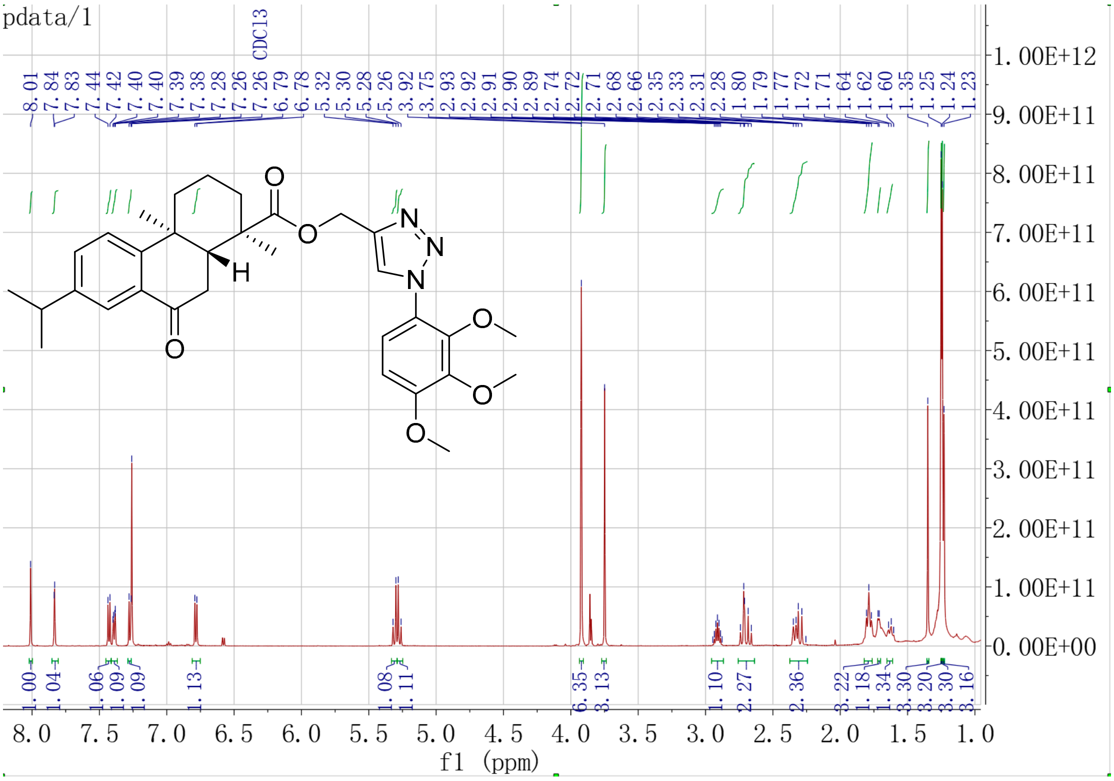

Figure S8-1. ^{1}H NMR spectrum of compound **13**

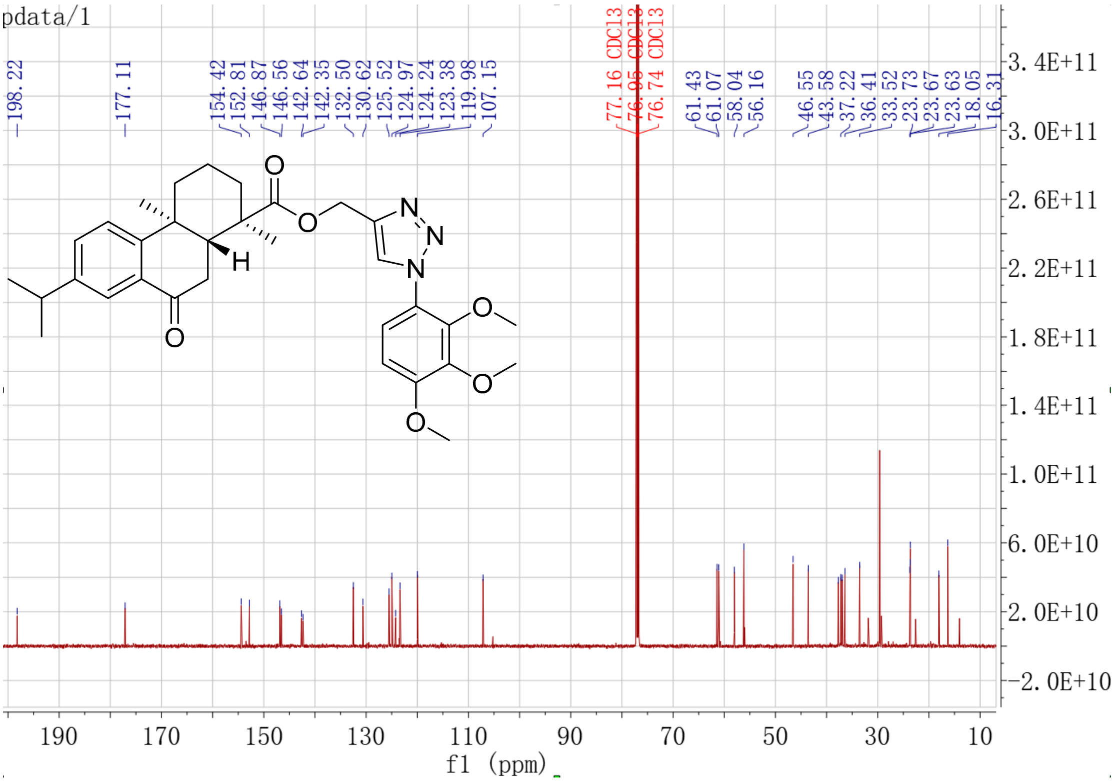

Figure S8-2. ^{13}C NMR spectrum of compound **13**

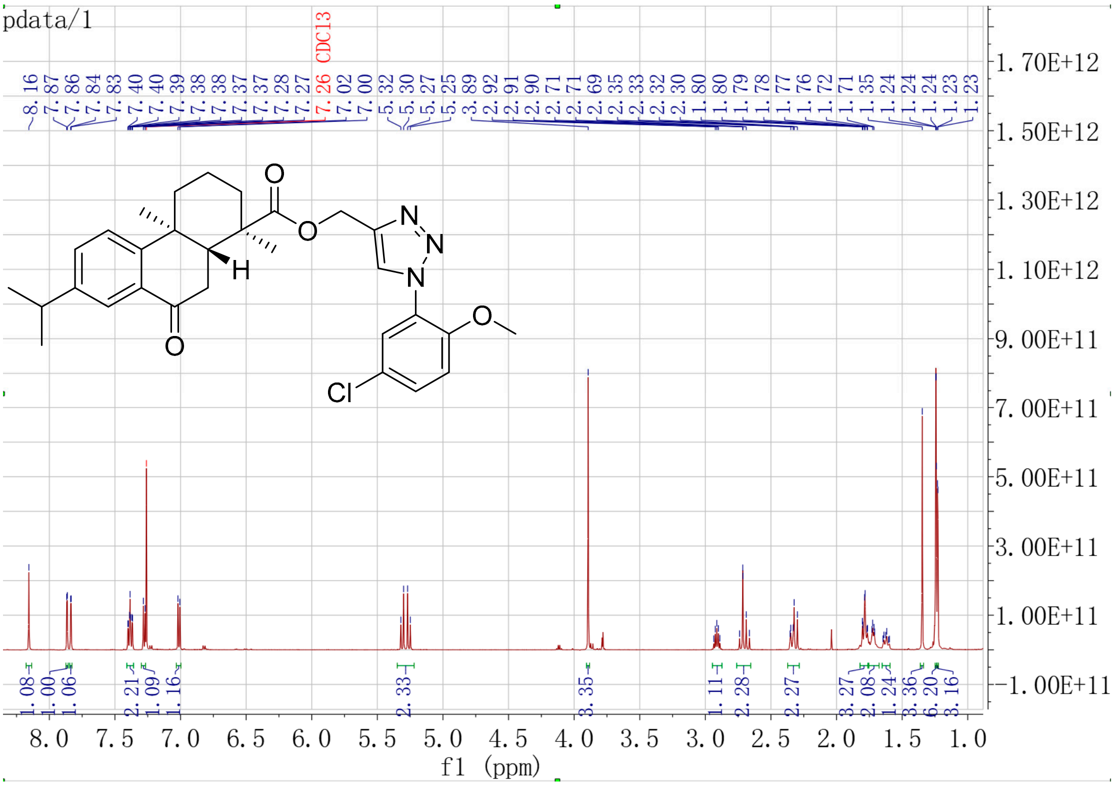

Figure S9-1. ^{1}H NMR spectrum of compound **14**

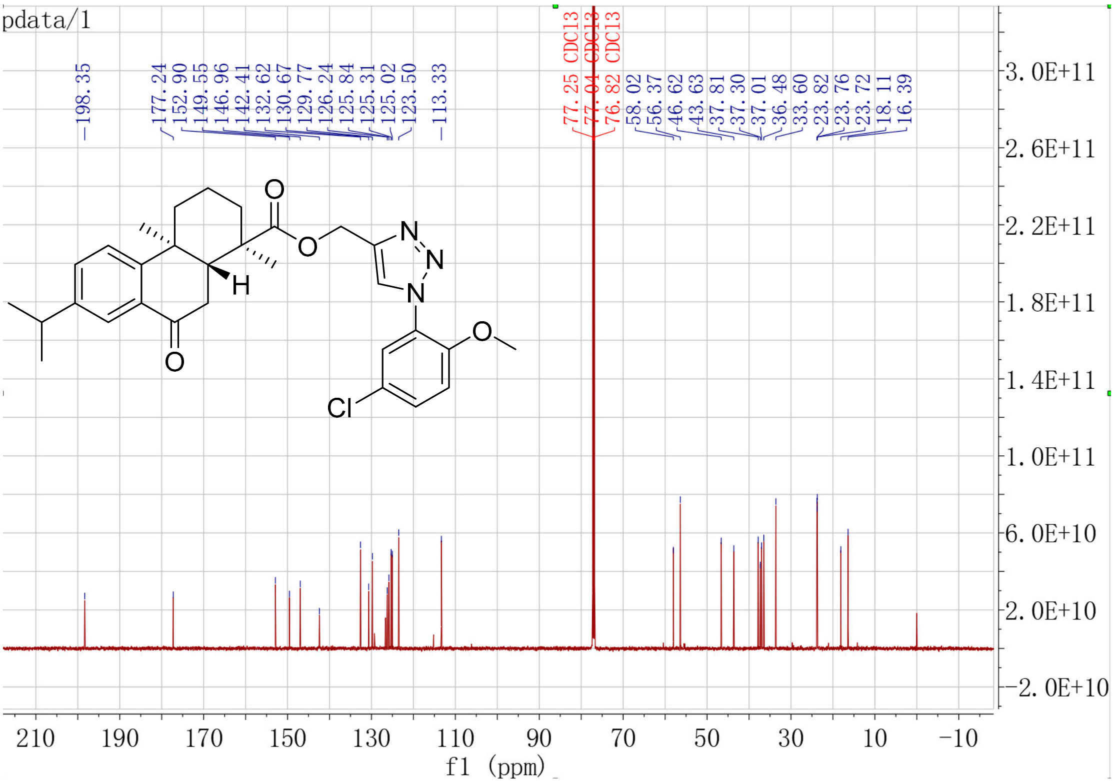

Figure S9-2. ^{13}C NMR spectrum of compound **14**

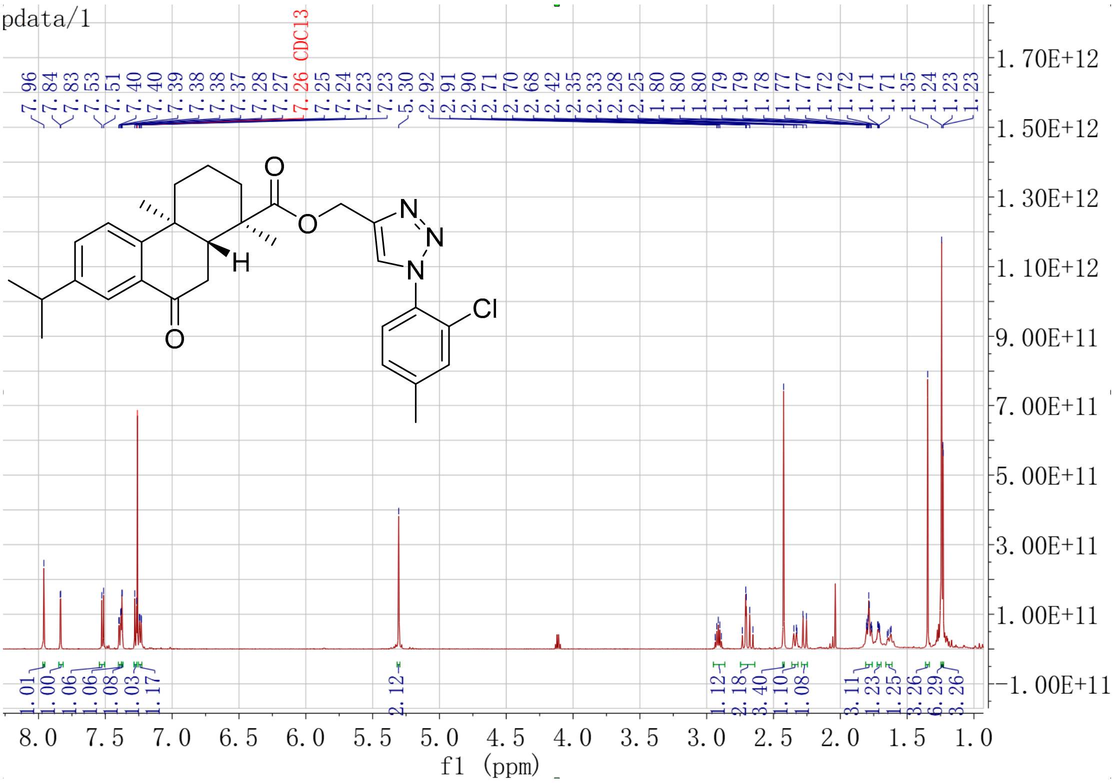

Figure S10-1. ^{1}H NMR spectrum of compound **15**

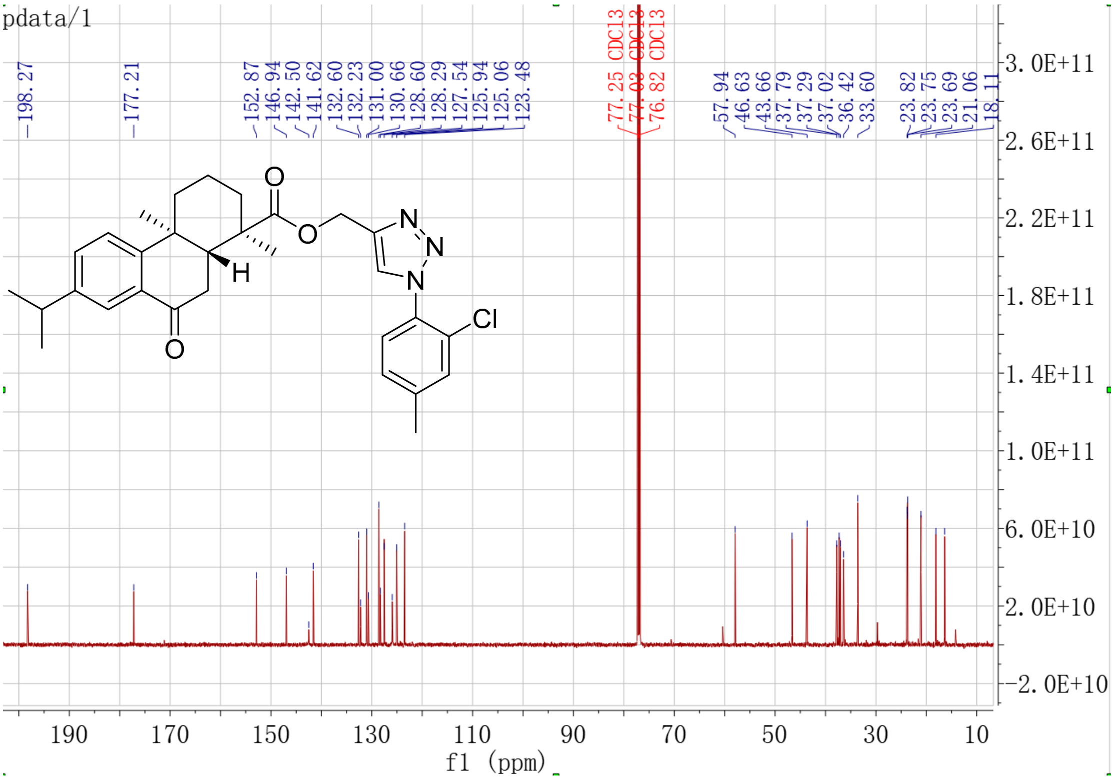

Figure S10-2. ^{13}C NMR spectrum of compound **15**

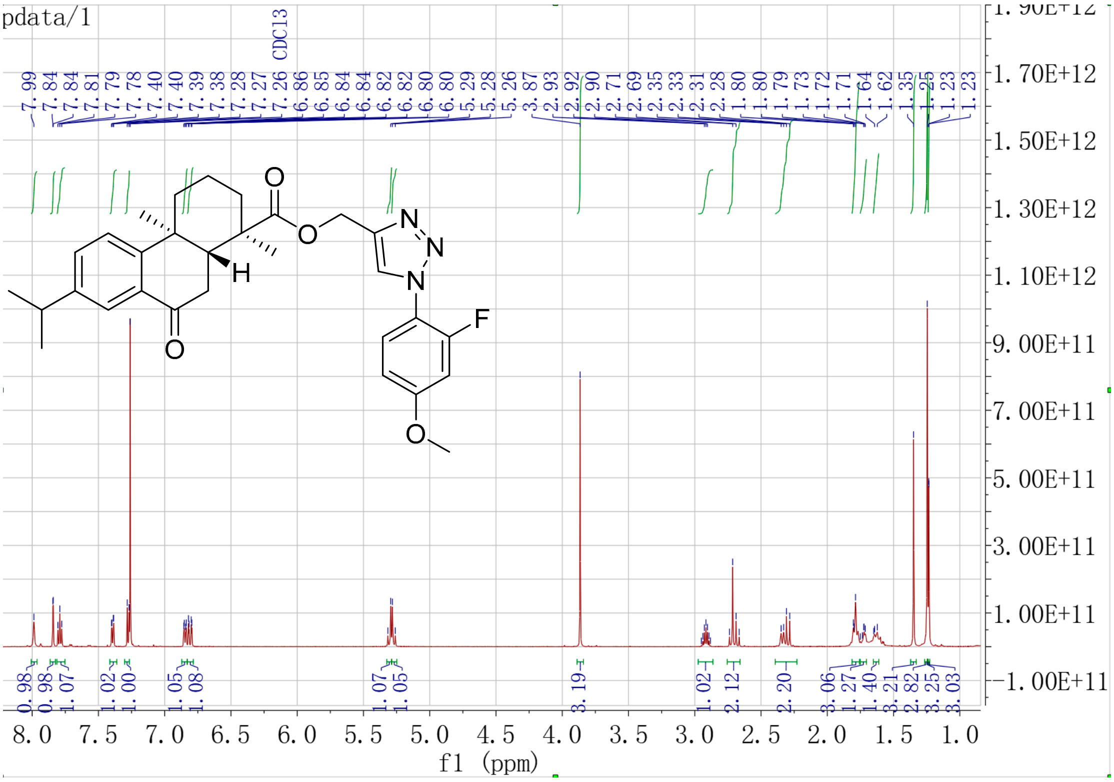

Figure S11-1. ^{1}H NMR spectrum of compound **16**

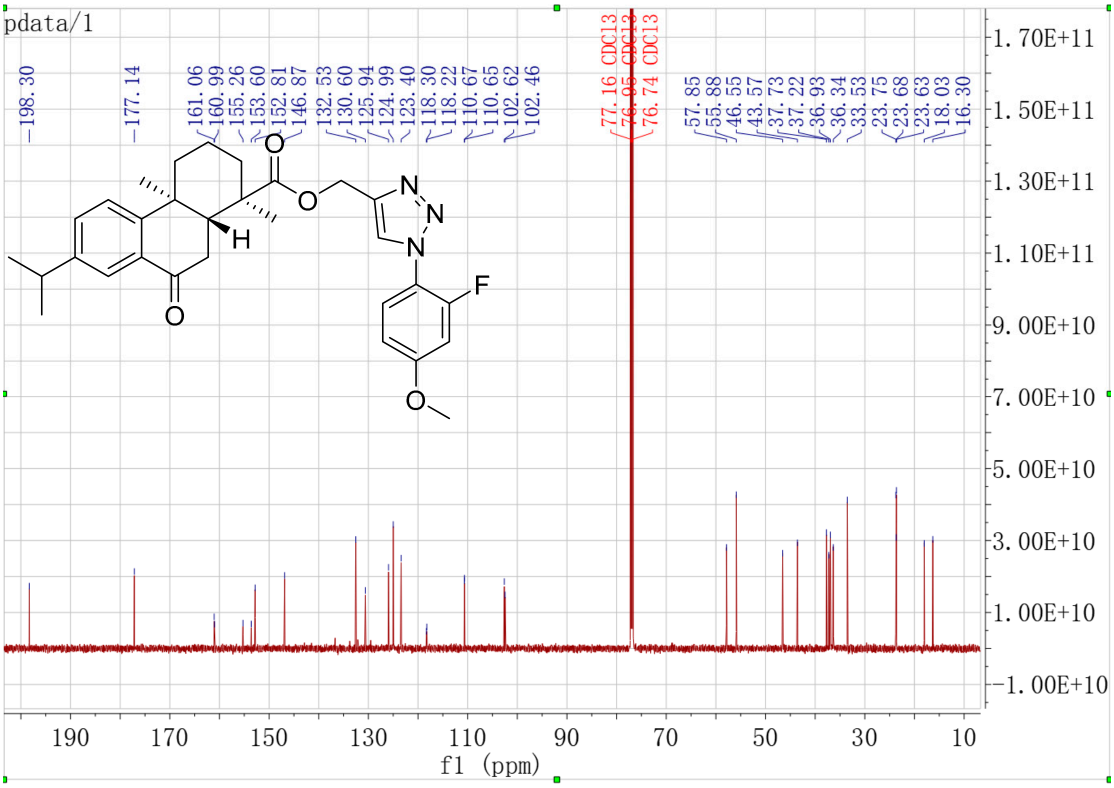

Figure S11-2. ^{13}C NMR spectrum of compound **16**

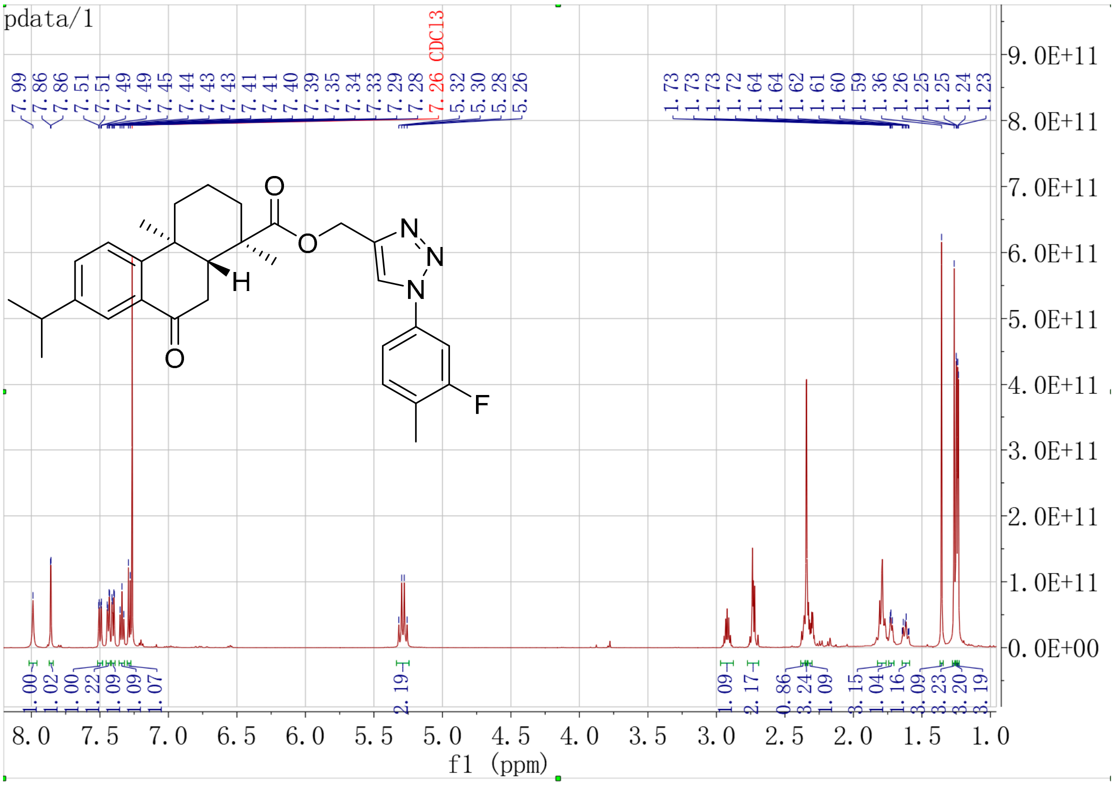

Figure S12-1. ^{1}H NMR spectrum of compound **17**

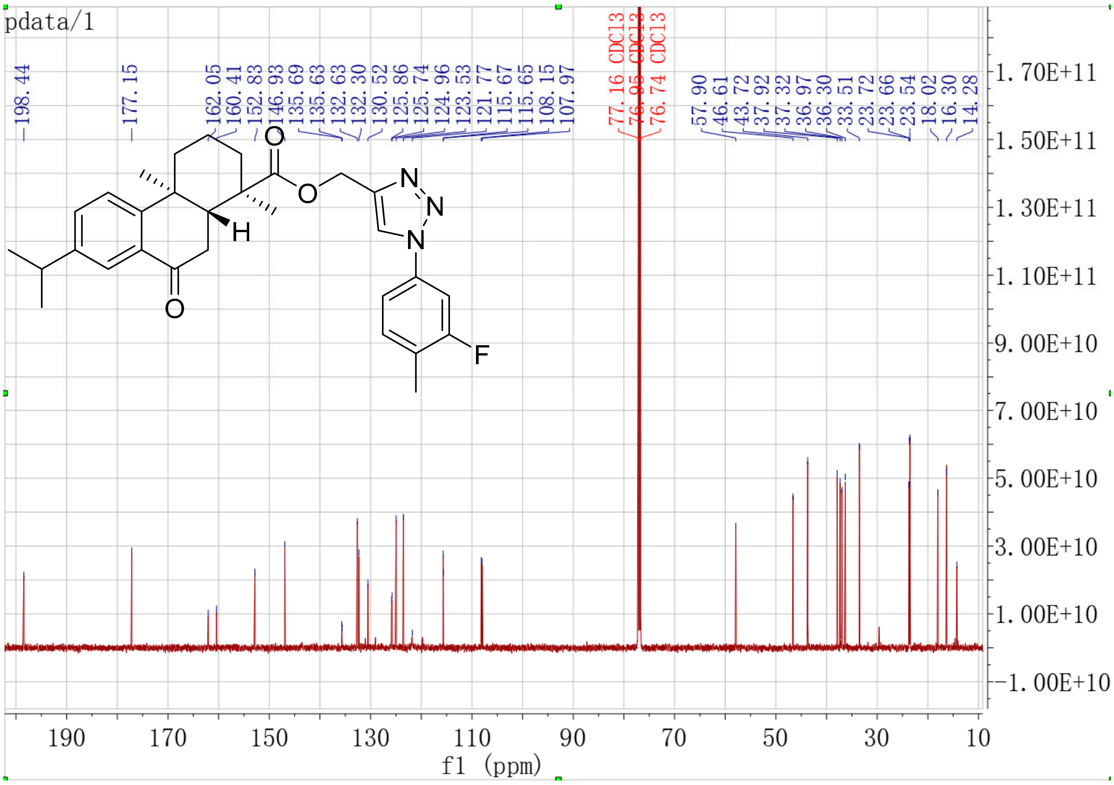

Figure S12-2. ^{13}C NMR spectrum of compound **17**

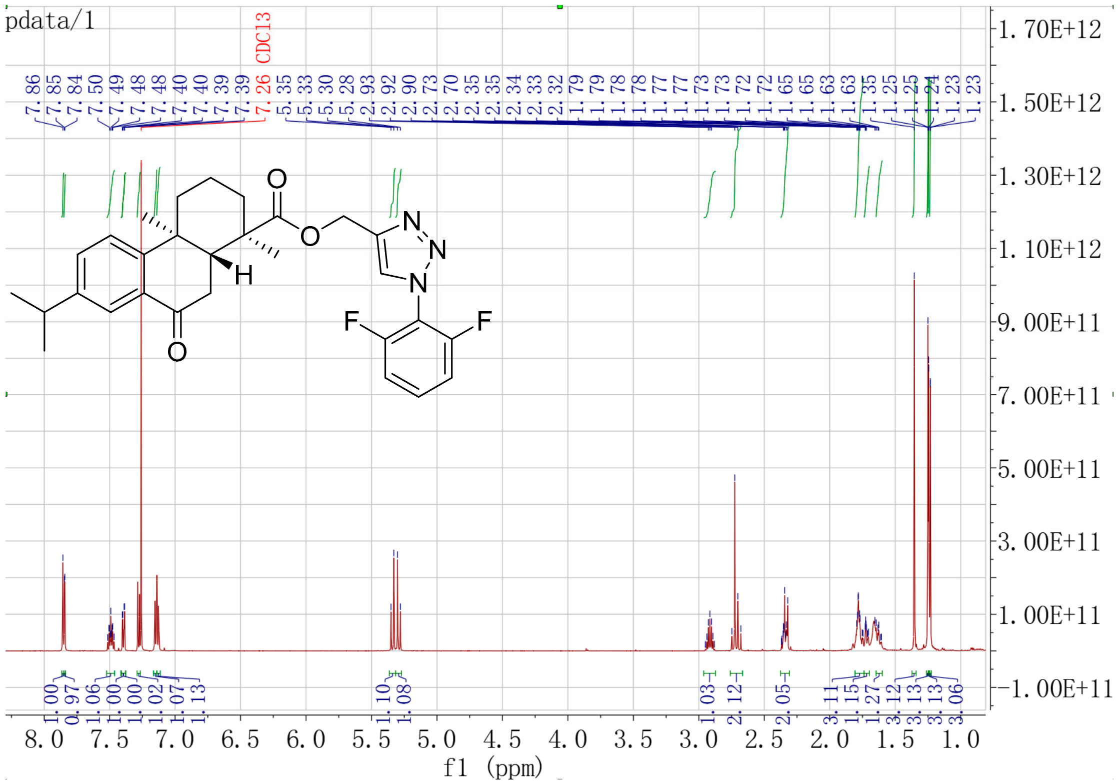

Figure S13-1. ^{1}H NMR spectrum of compound **18**

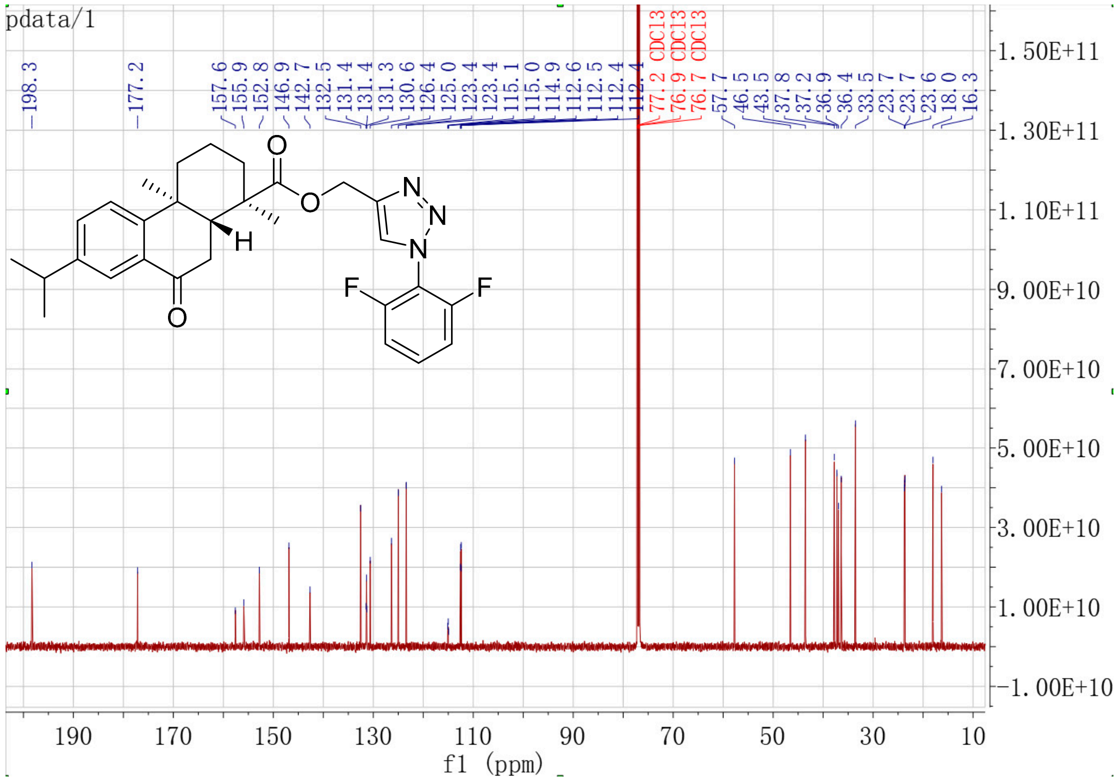

Figure S13-2. ^{13}C NMR spectrum of compound **18**

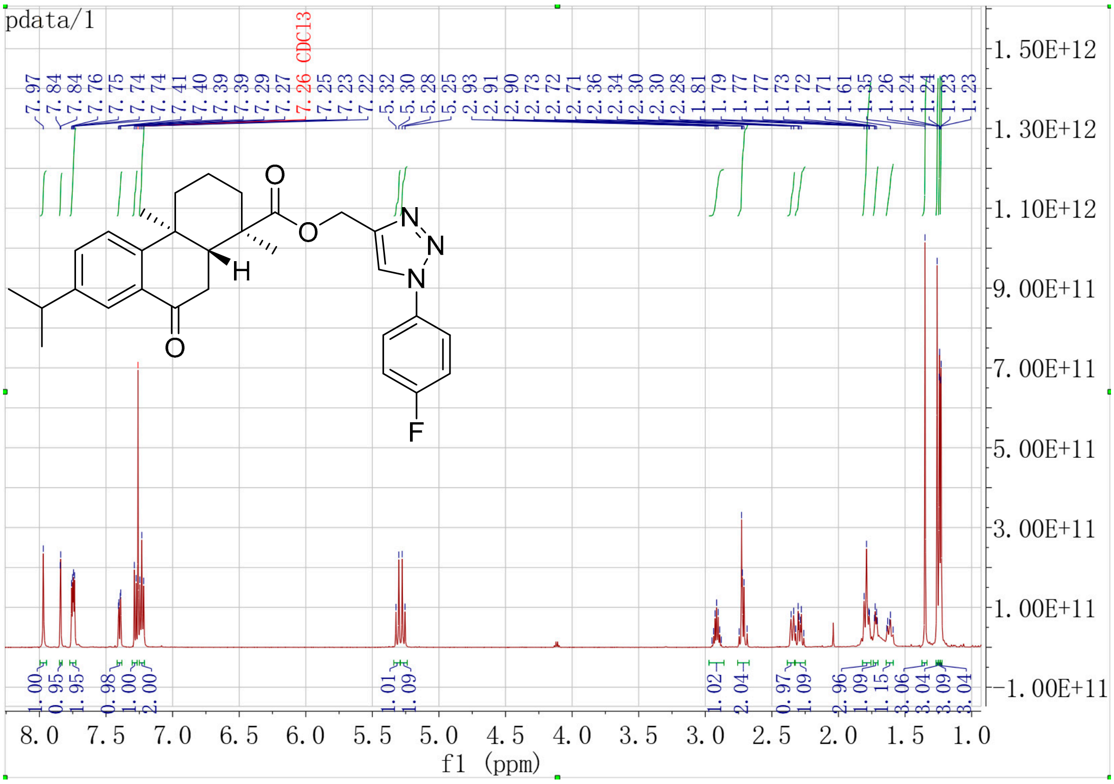

Figure S14-1. ^{1}H NMR spectrum of compound **19**

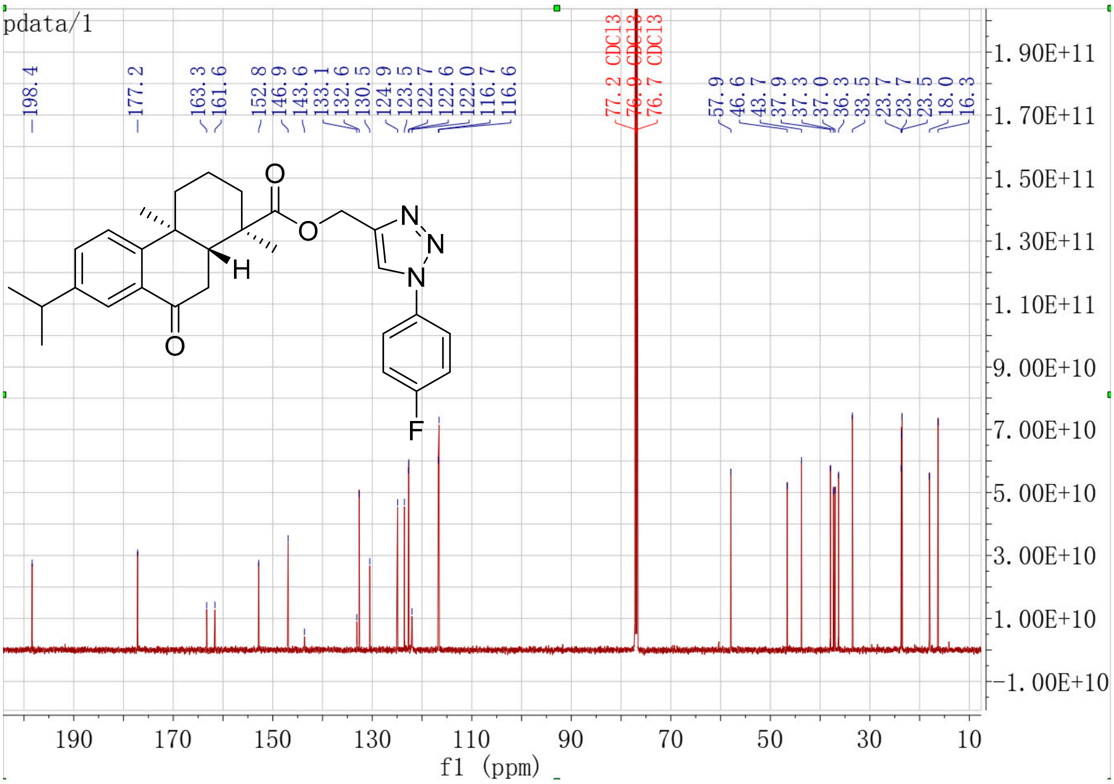

Figure S14-2. ^{13}C NMR spectrum of compound **19**

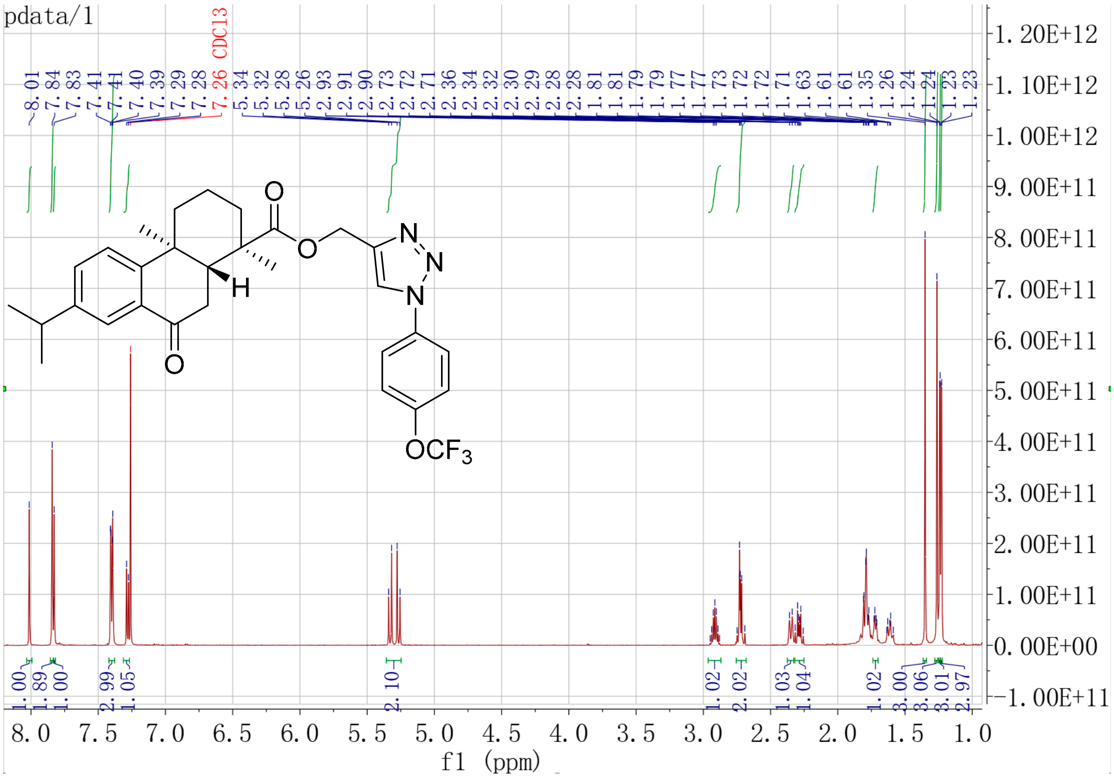

Figure S15-1. ^{1}H NMR spectrum of compound **20**

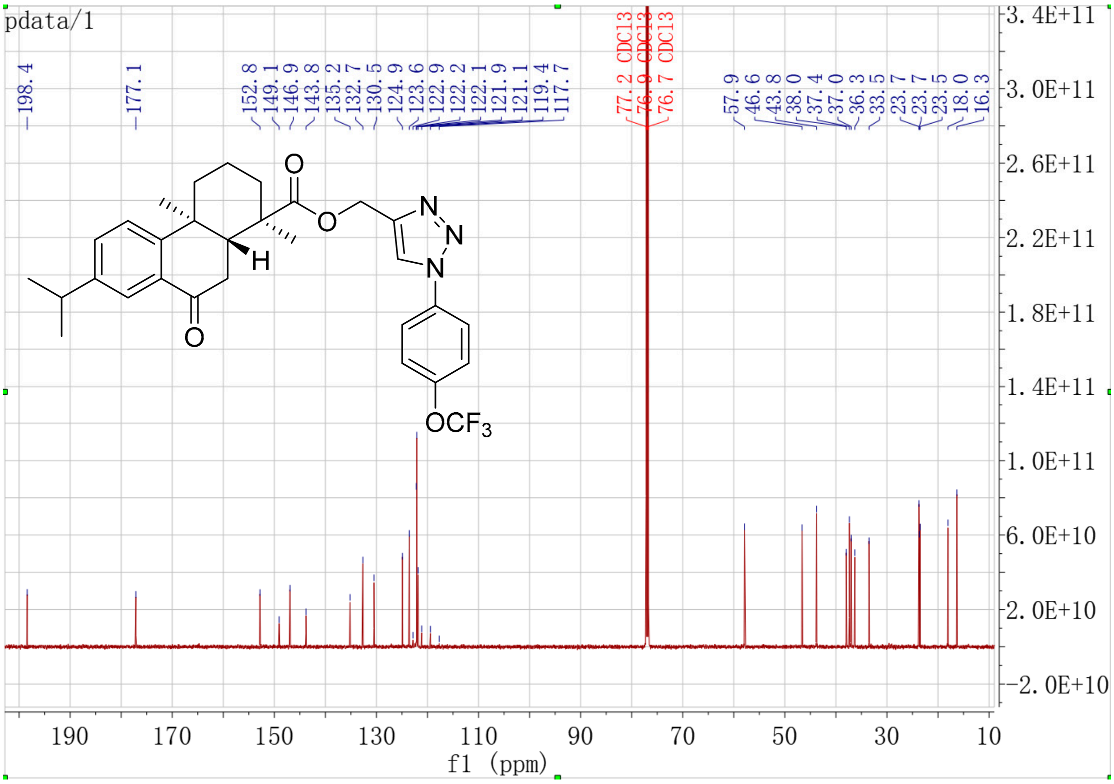

Figure S15-2. ^{13}C NMR spectrum of compound **20**

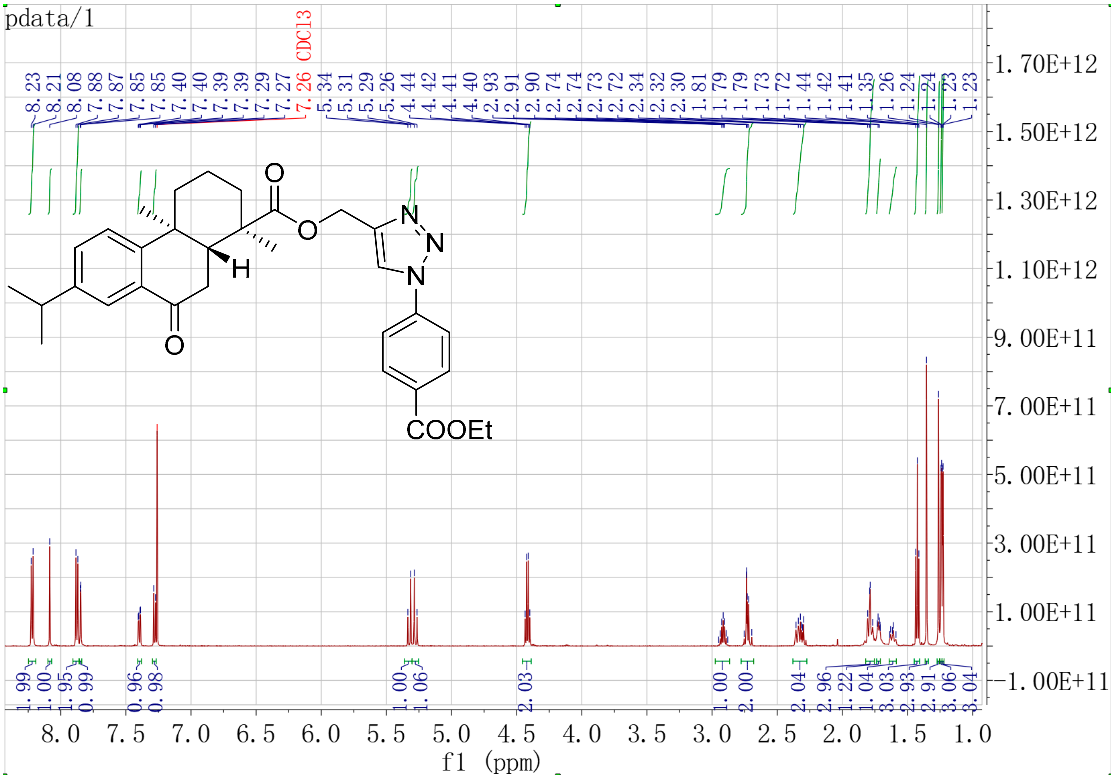

Figure S16-1. ^{1}H NMR spectrum of compound **21**

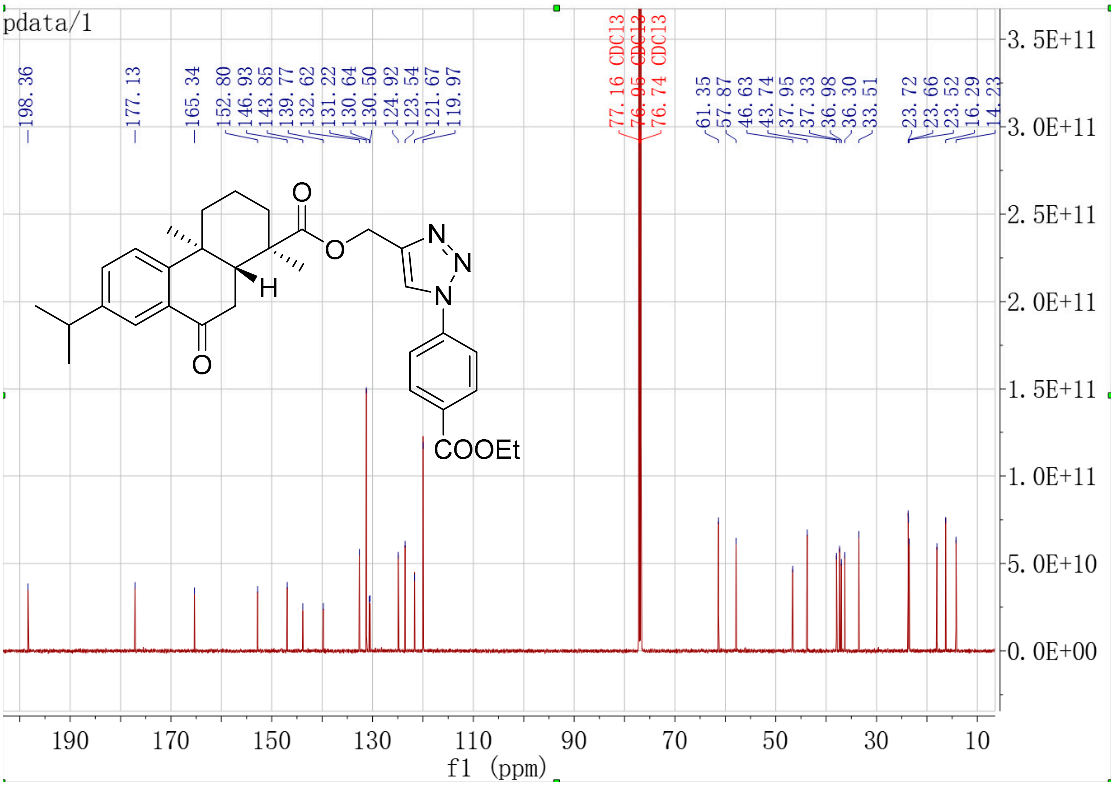

Figure S16-2. ^{13}C NMR spectrum of compound **21**

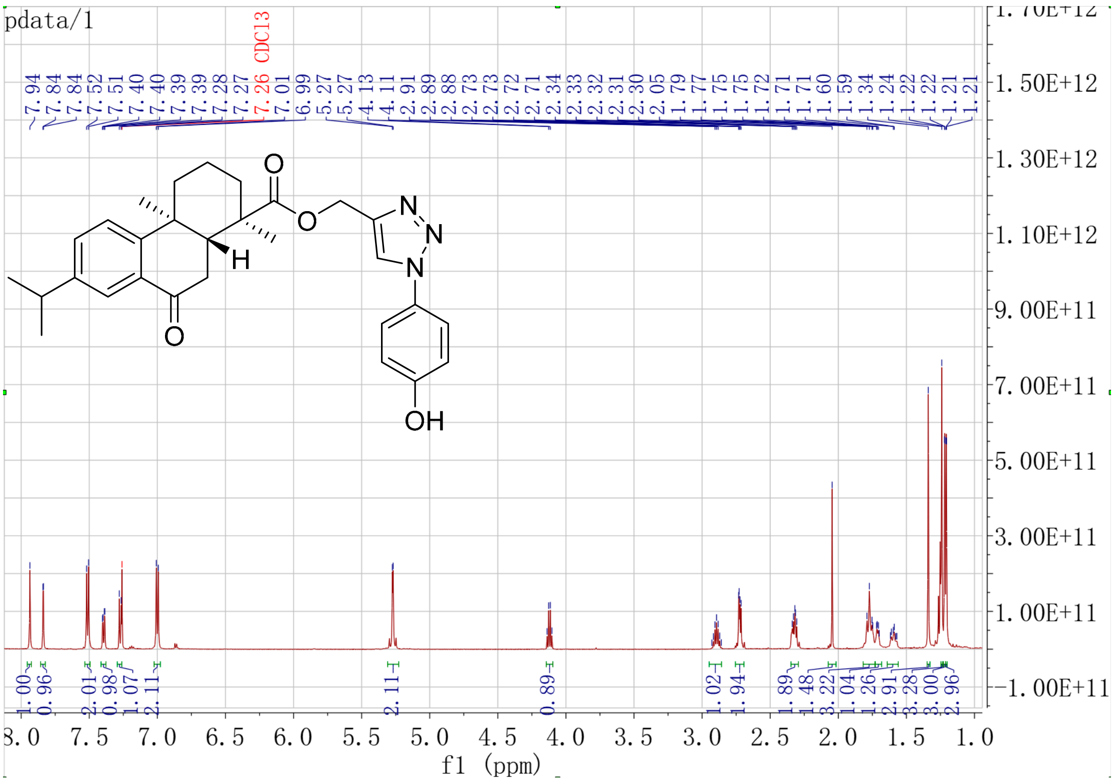

Figure S17-1. ^{1}H NMR spectrum of compound **22**

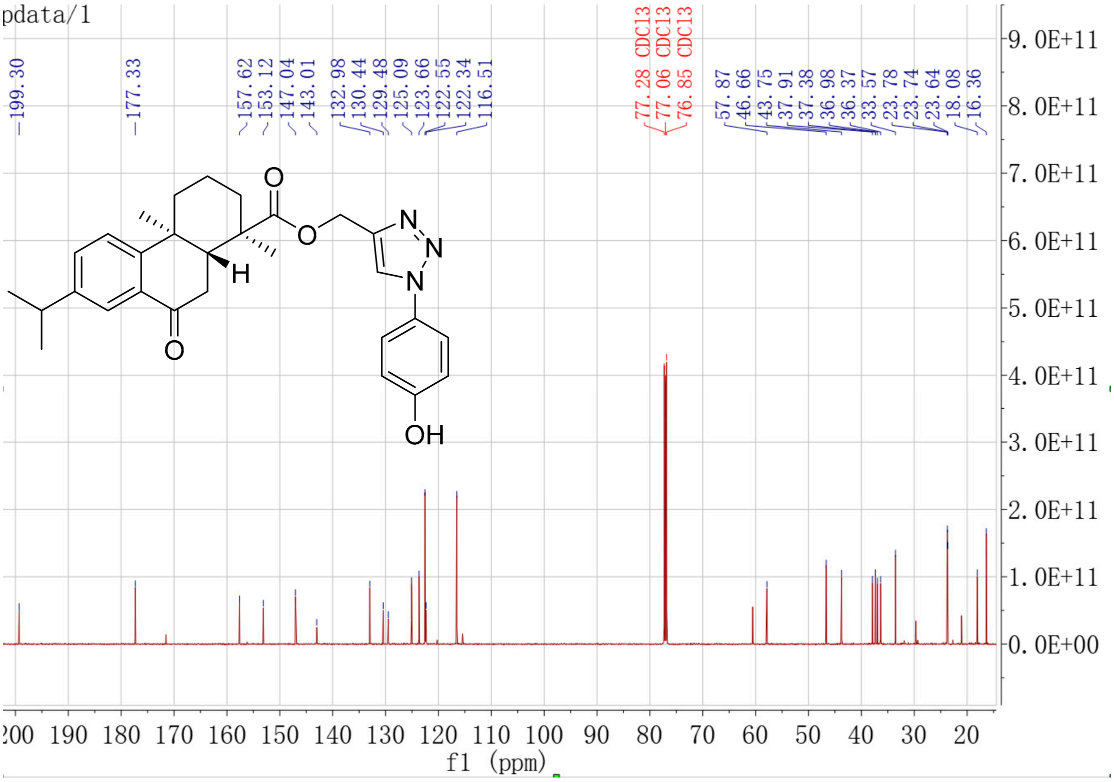

Figure S17-2. ^{13}C NMR spectrum of compound **22**

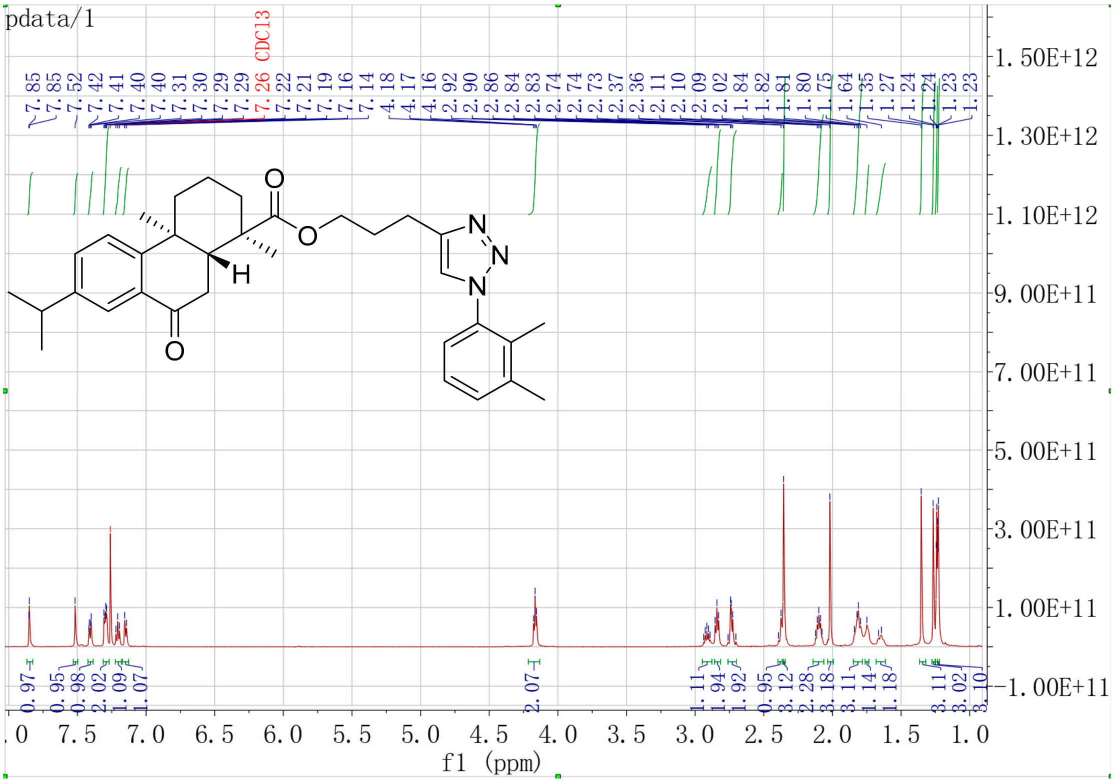

Figure S18-1. ^{1}H NMR spectrum of compound **23**

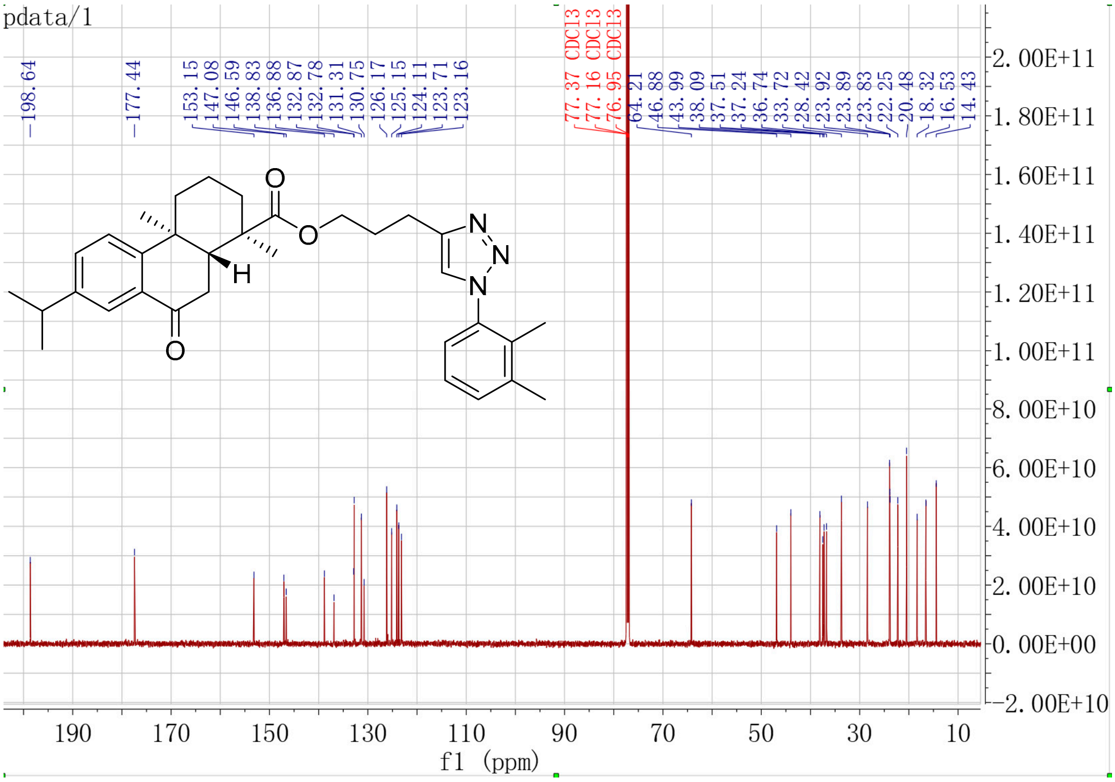

Figure S18-2. ^{13}C NMR spectrum of compound **23**

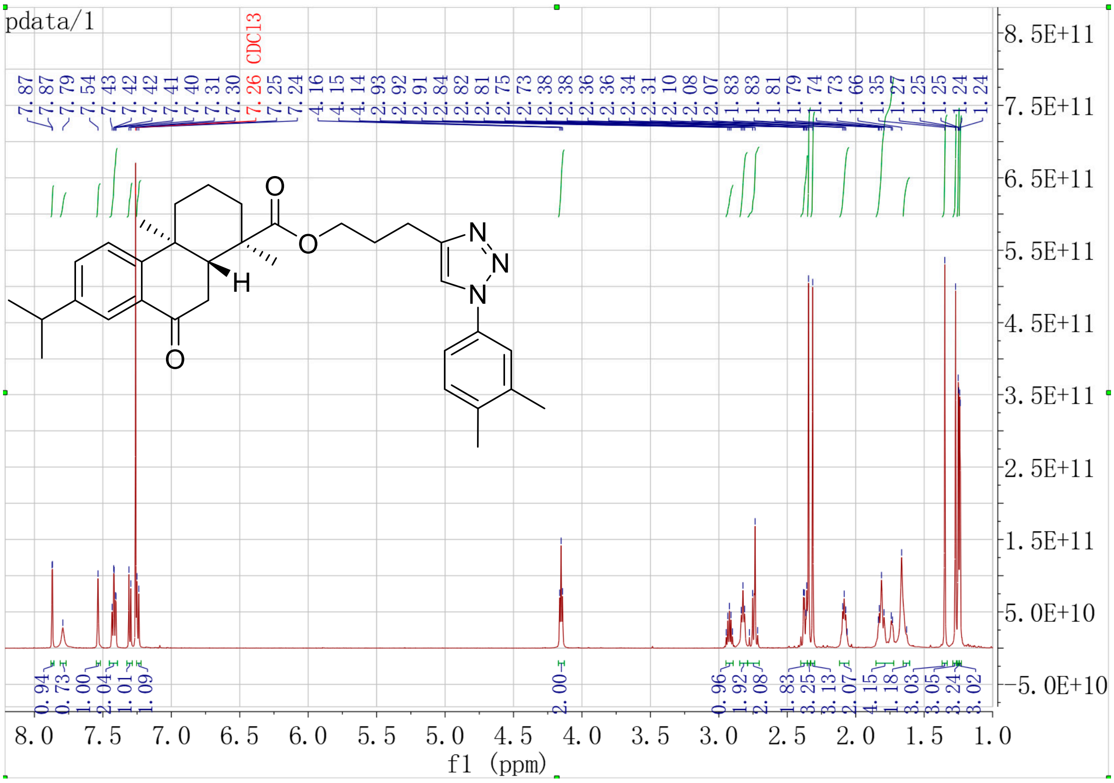

Figure S19-1. ^{1}H NMR spectrum of compound **24**

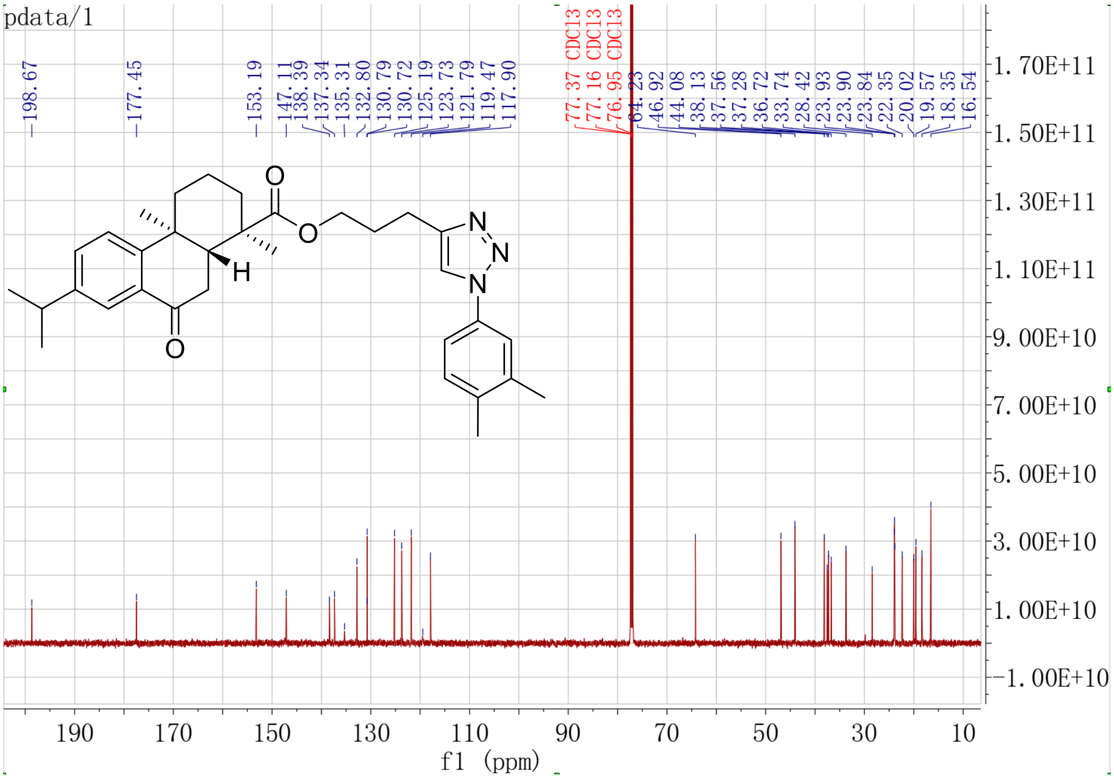

Figure S19-2. ^{13}C NMR spectrum of compound **24**

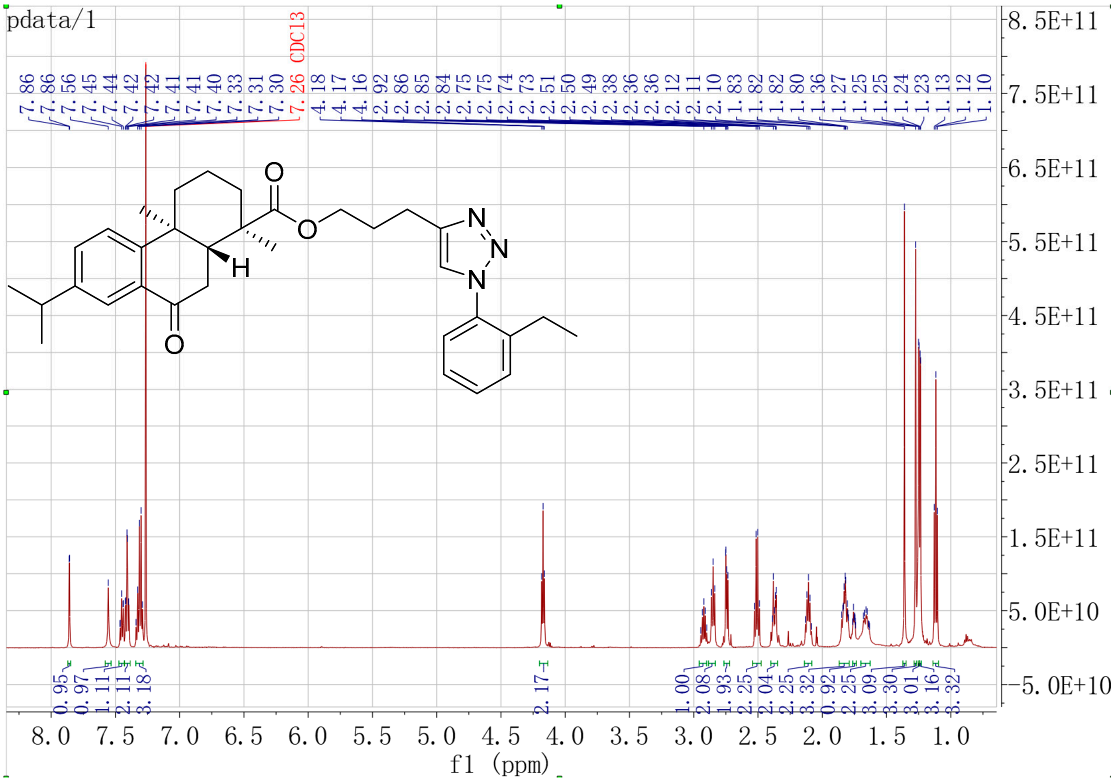

Figure S20-1. ^{1}H NMR spectrum of compound **25**

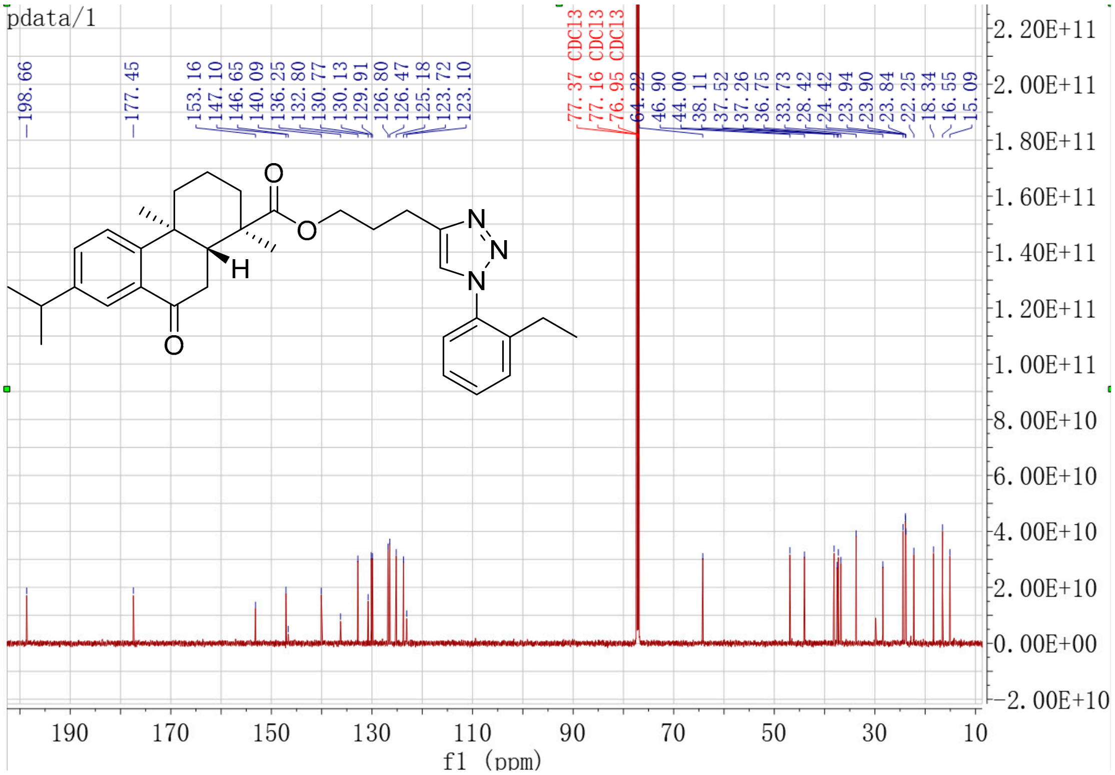

Figure S20-2. ^{13}C NMR spectrum of compound **25**

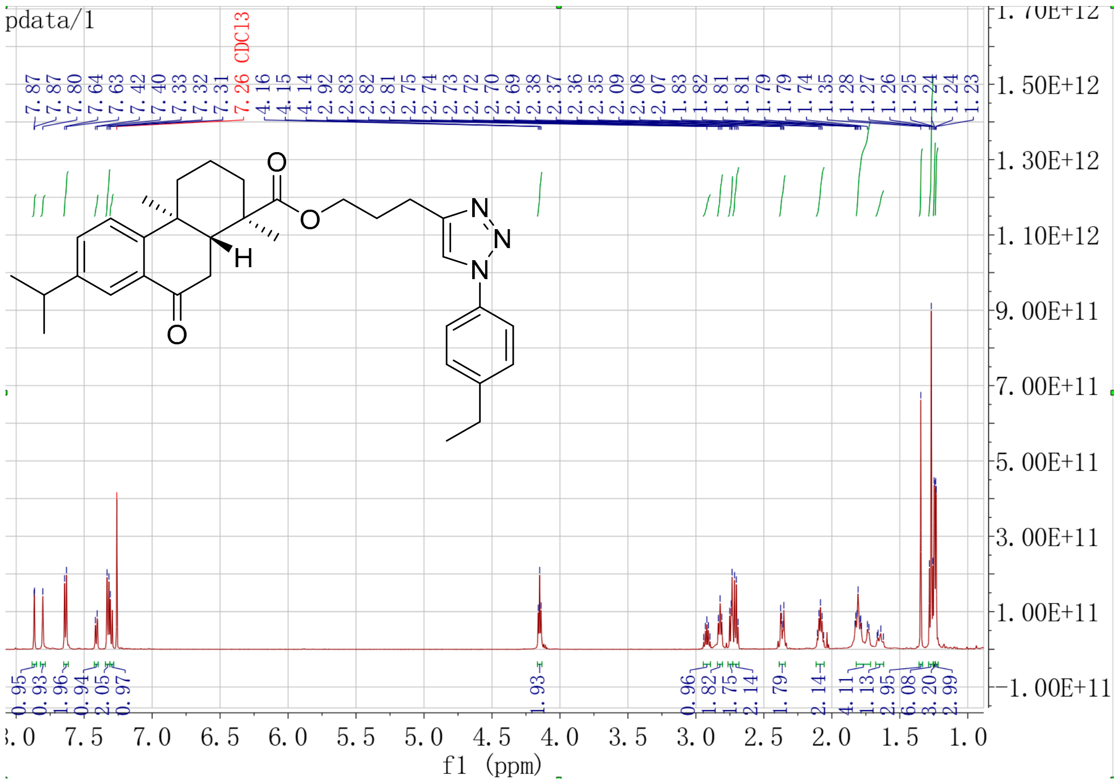

Figure S21-1. ^{1}H NMR spectrum of compound **26**

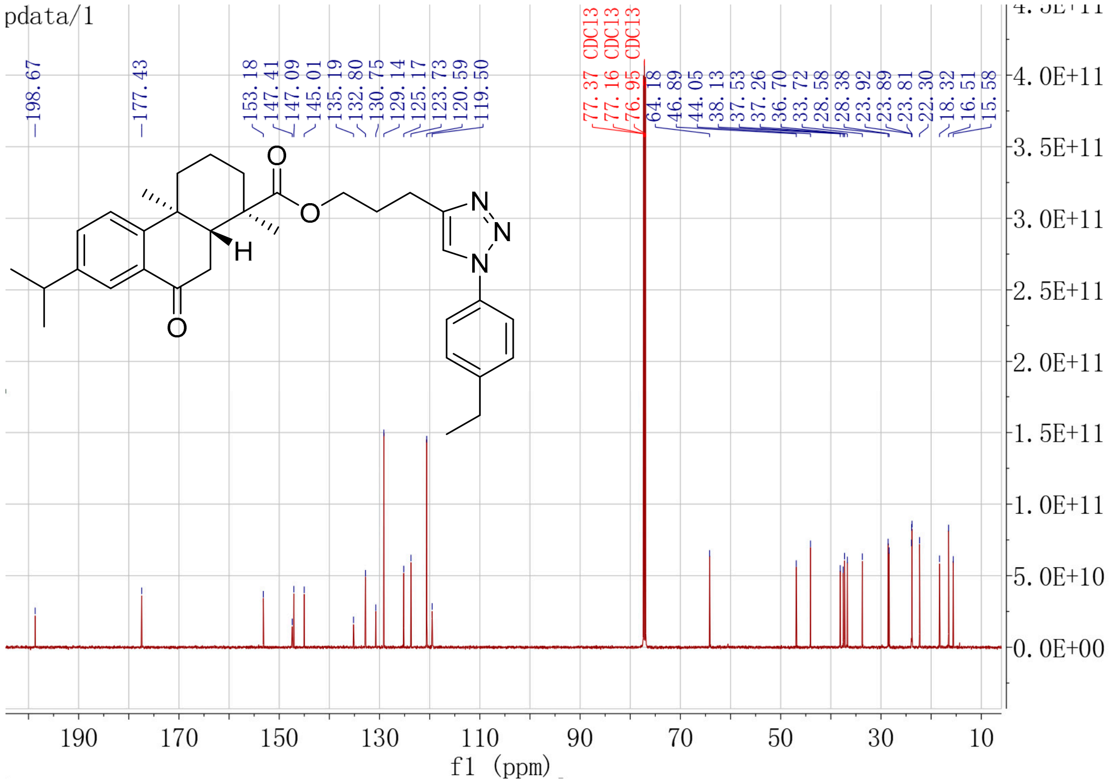

Figure S21-2. ^{13}C NMR spectrum of compound **26**

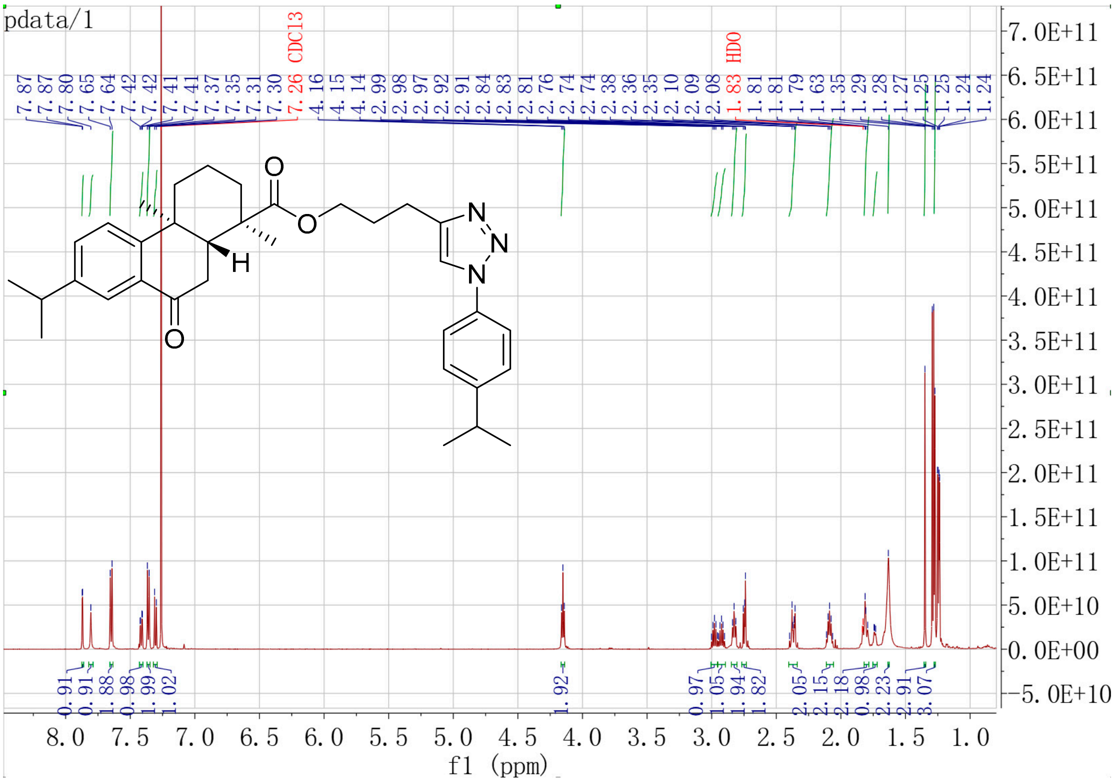

Figure S22-1. ^{1}H NMR spectrum of compound **27**

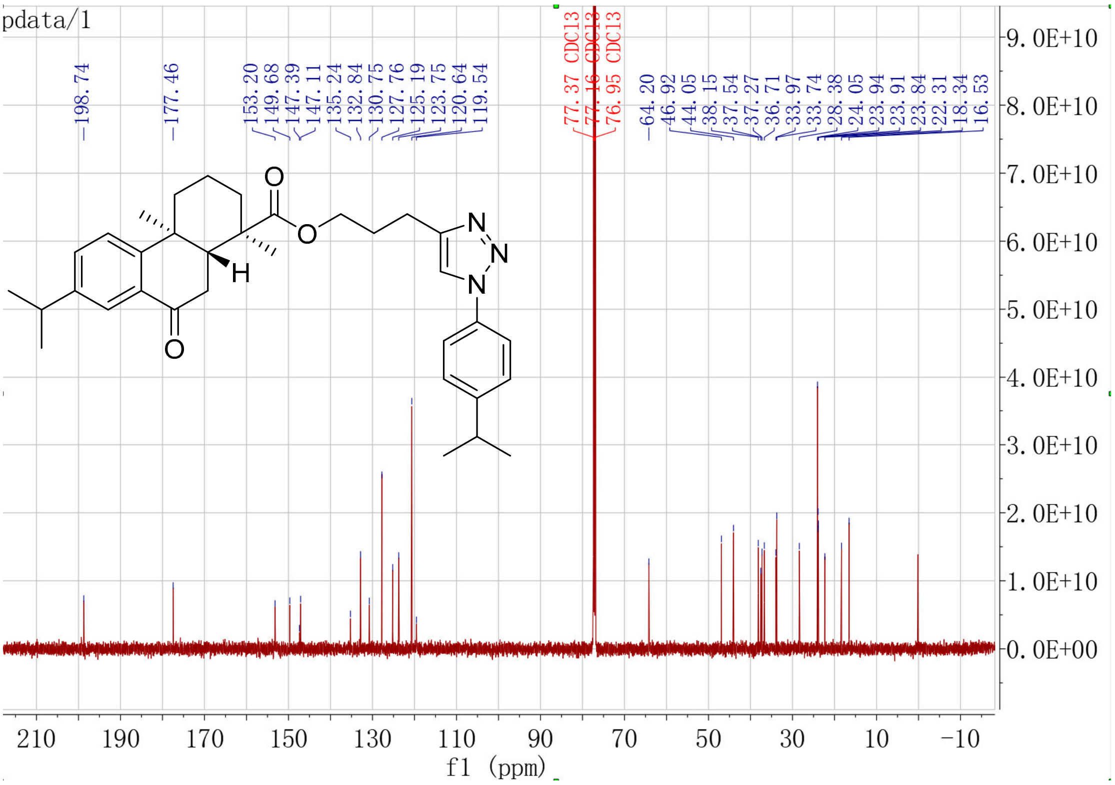

Figure S22-2. ^{13}C NMR spectrum of compound **27**

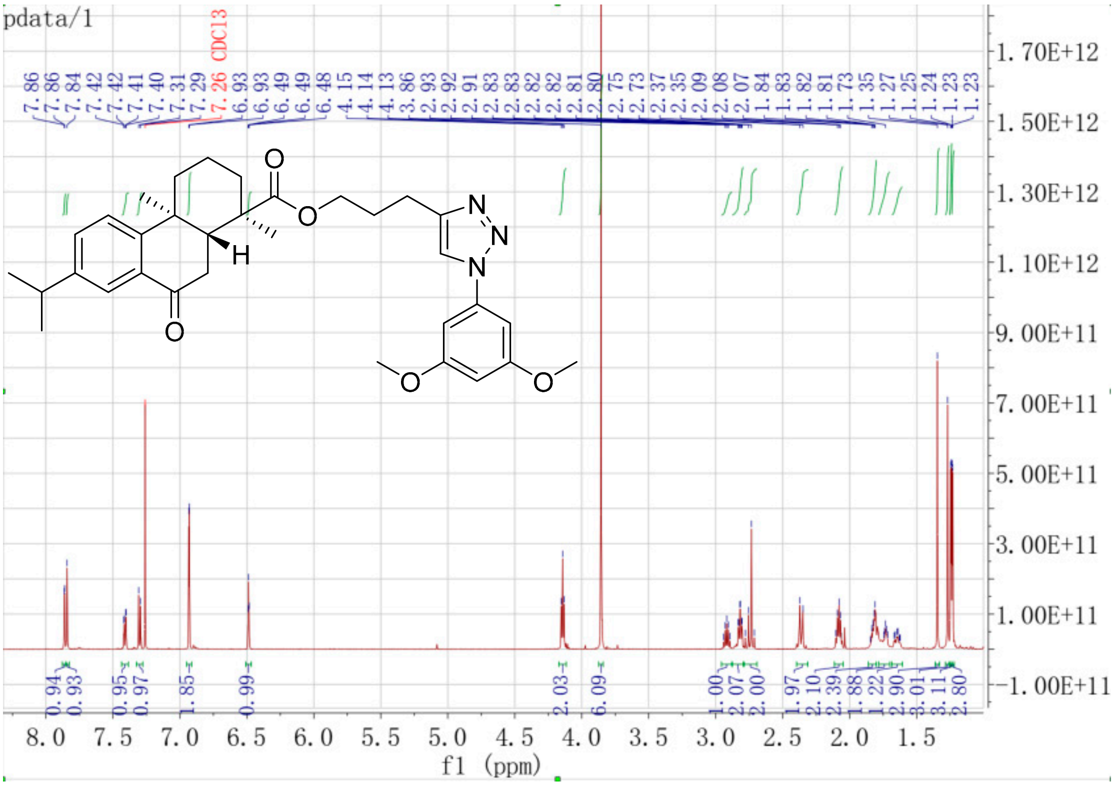

Figure S23-1. ^{1}H NMR spectrum of compound **28**

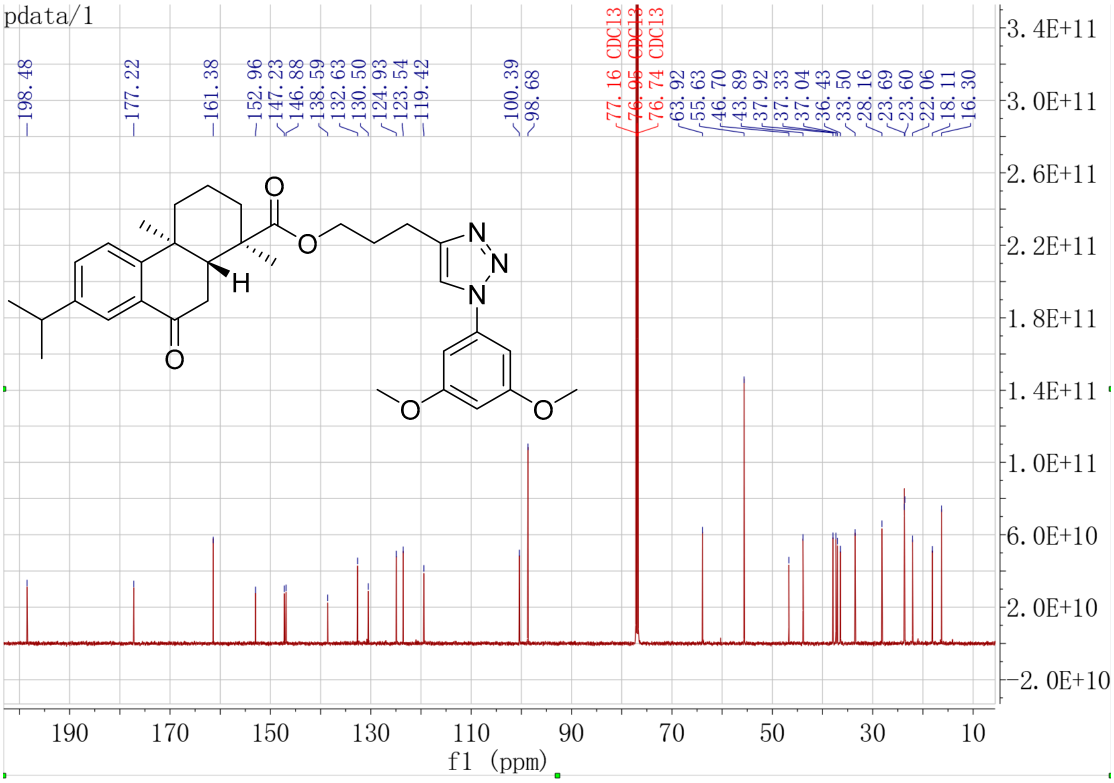

Figure S23-2. ^{13}C NMR spectrum of compound **28**

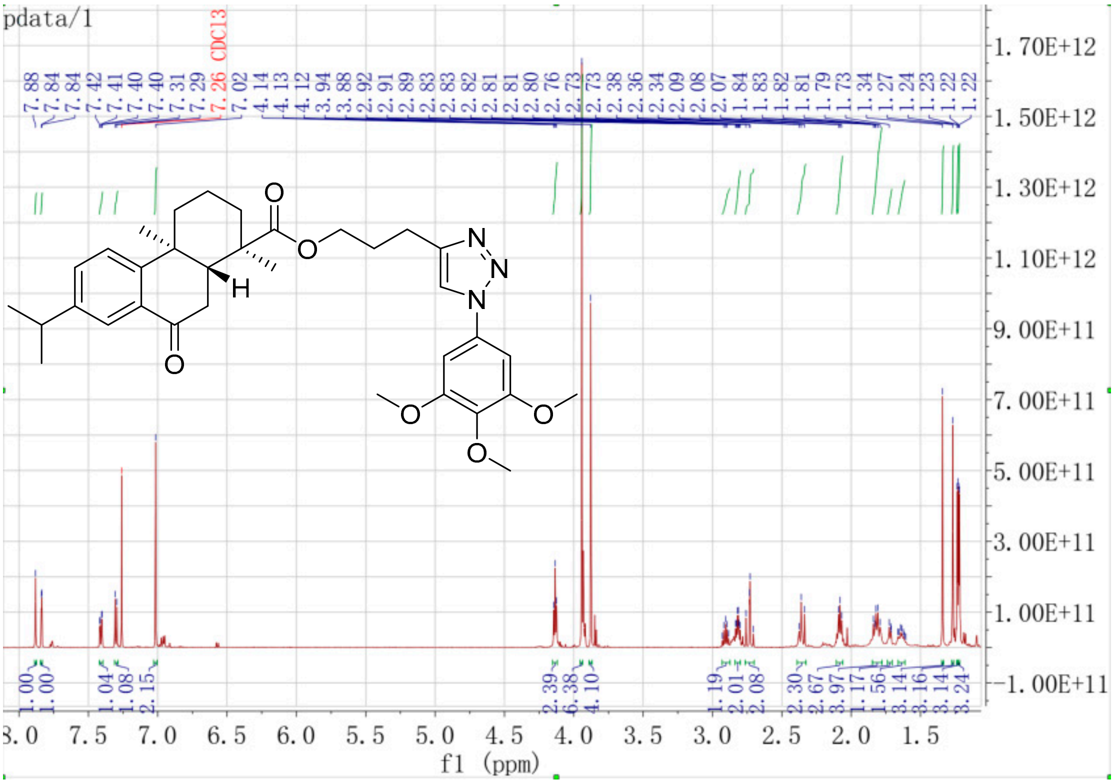

Figure S24-1. ^{1}H NMR spectrum of compound **29**

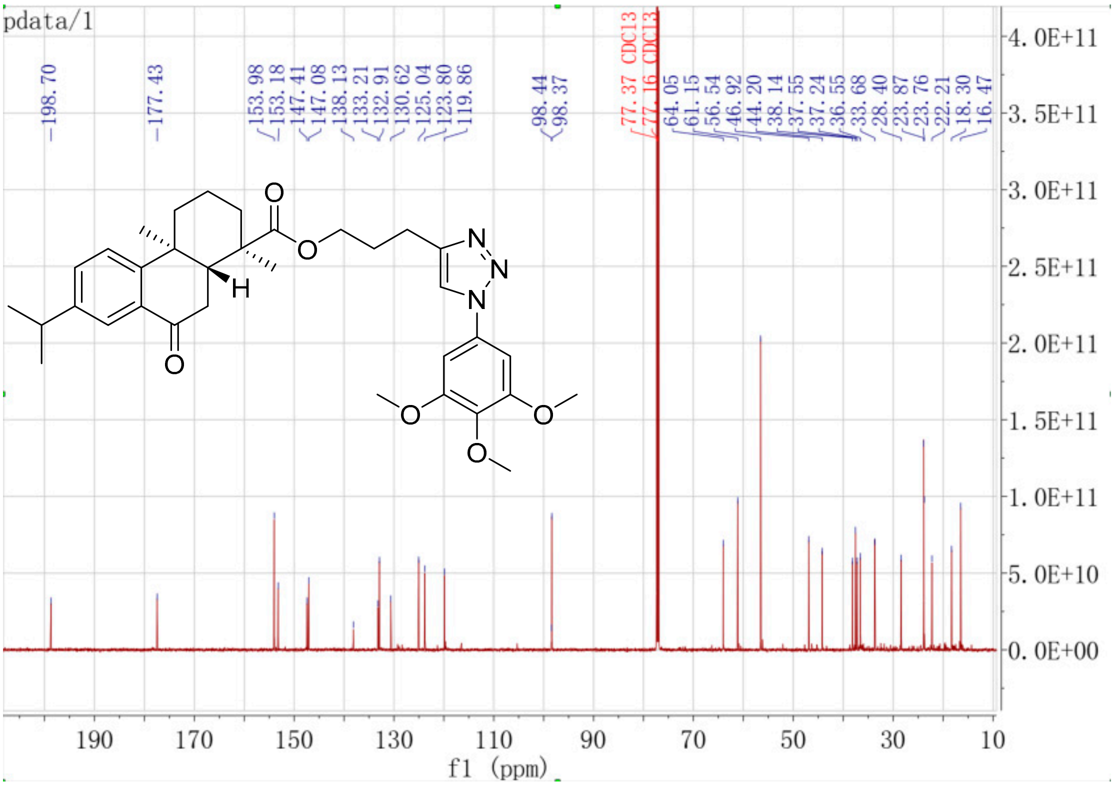

Figure S24-2. ^{13}C NMR spectrum of compound **29**

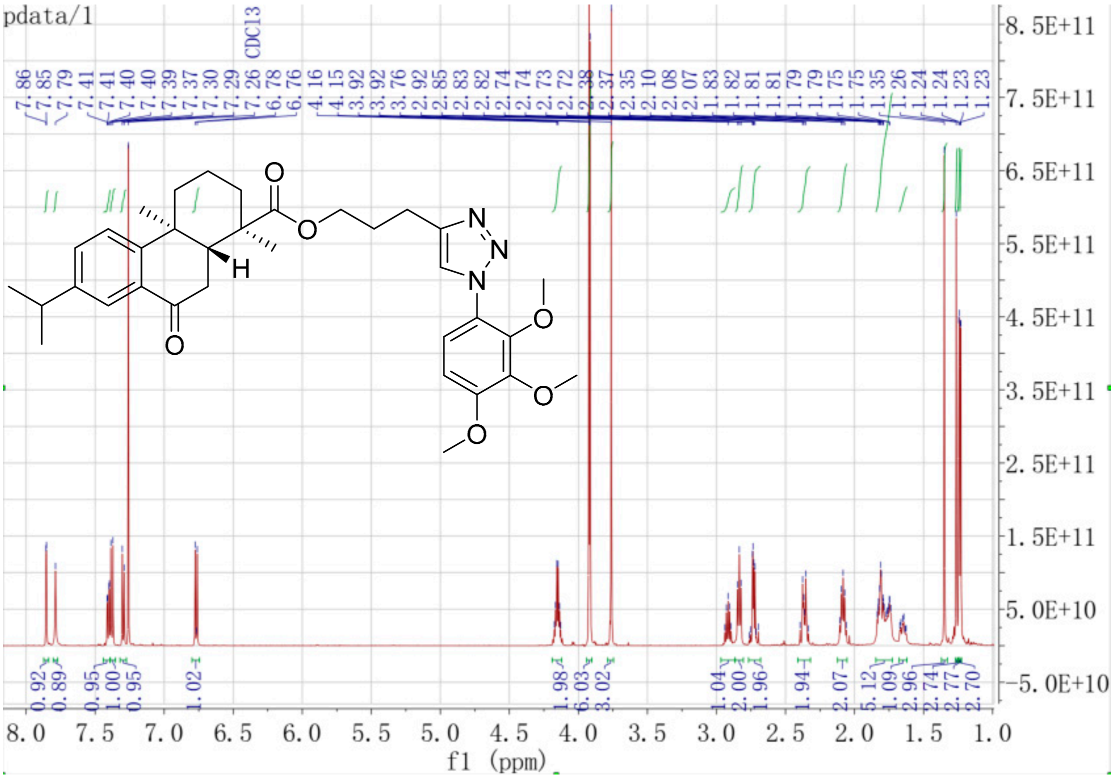

Figure S25-1. ^{1}H NMR spectrum of compound **30**

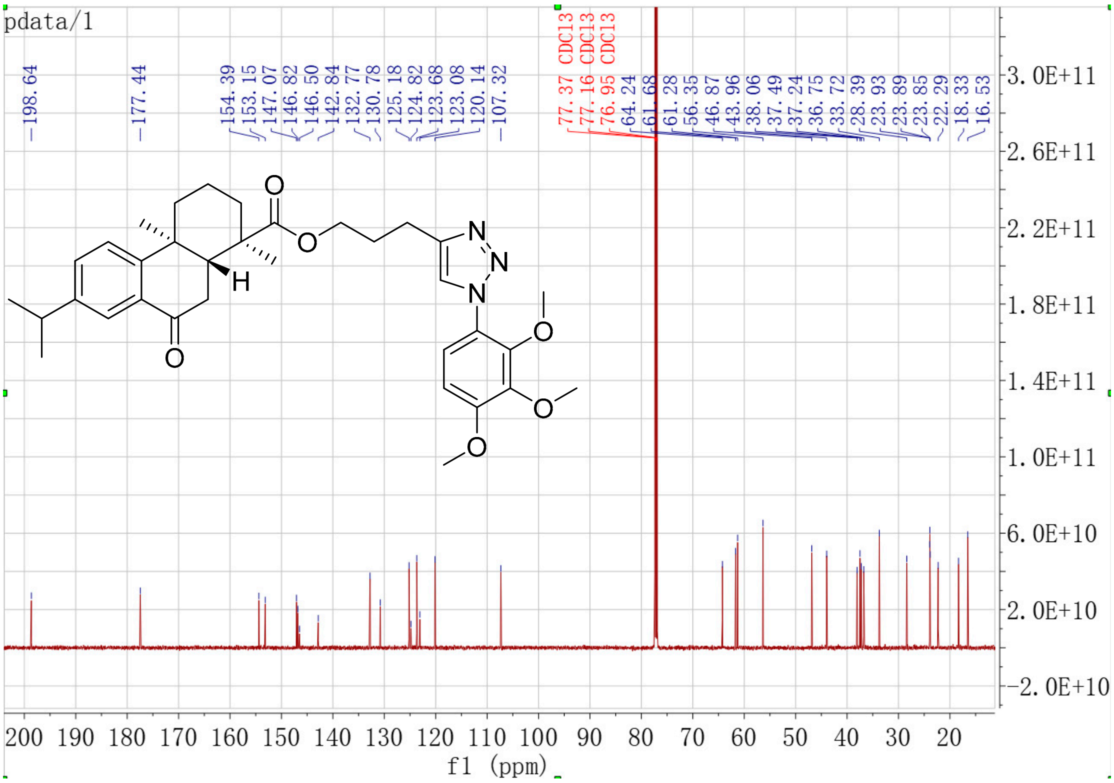

Figure S25-2. ^{13}C NMR spectrum of compound **30**

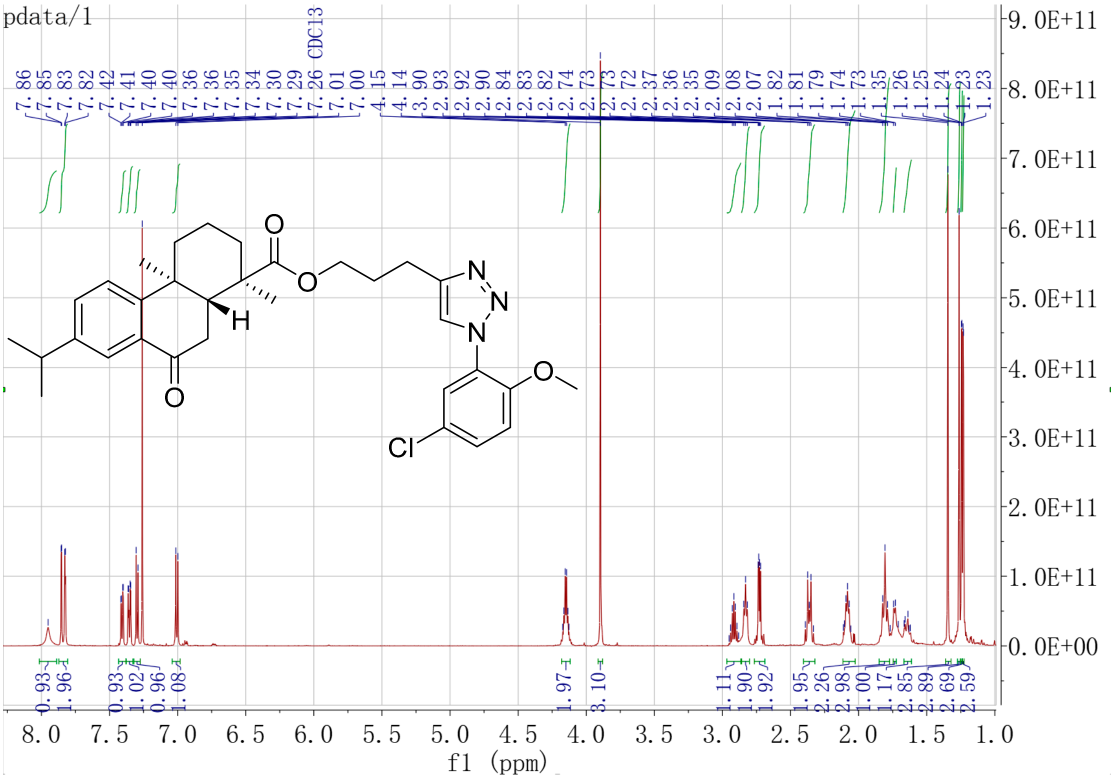

Figure S26-1. ^{1}H NMR spectrum of compound **31**

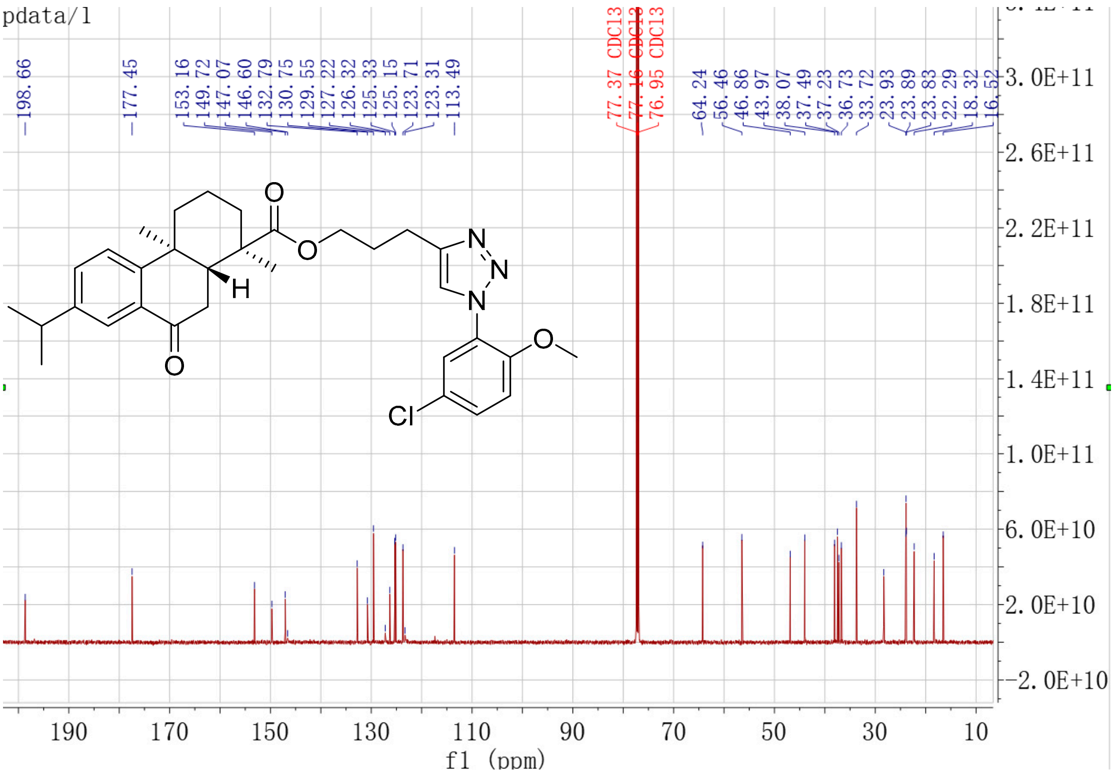

Figure S26-2. ^{13}C NMR spectrum of compound **31**

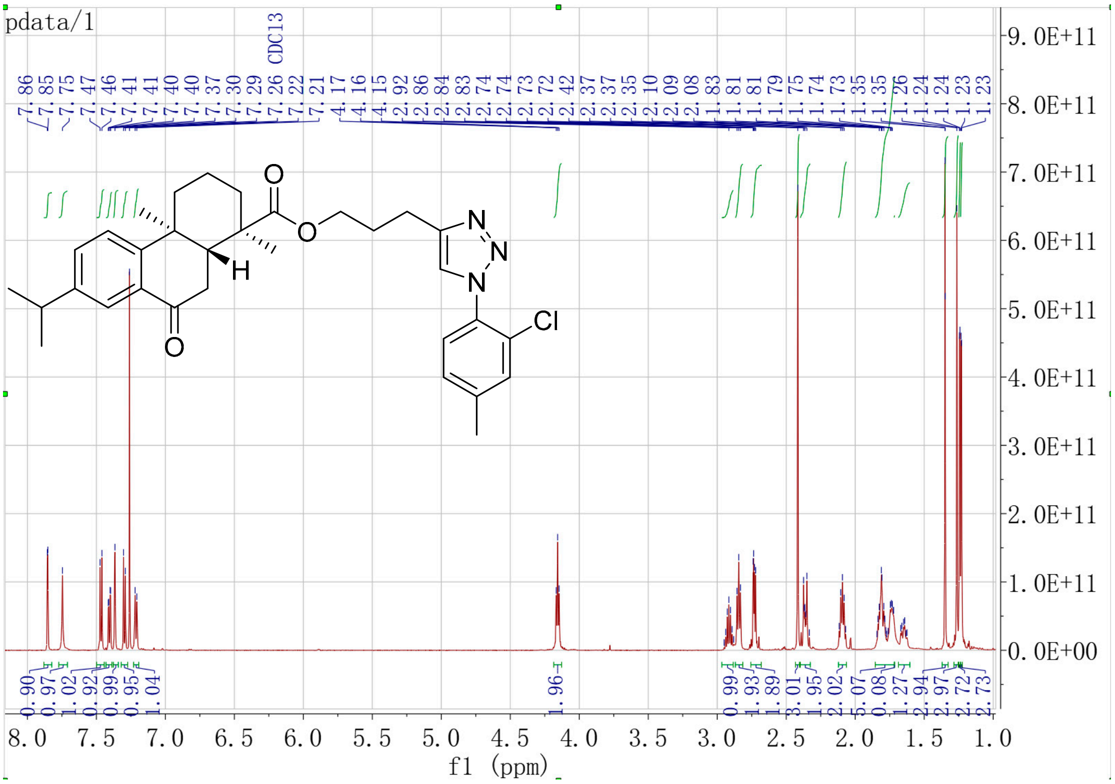

Figure S27-1. ^{1}H NMR spectrum of compound **32**

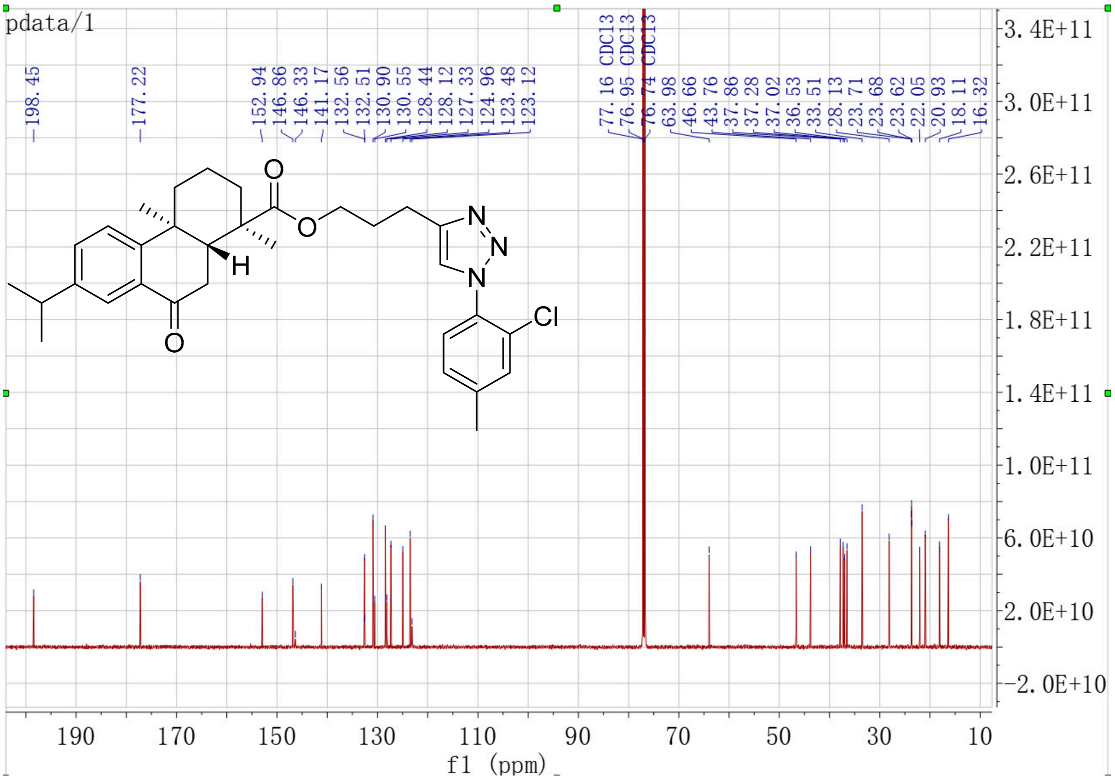

Figure S27-2. ^{13}C NMR spectrum of compound **32**

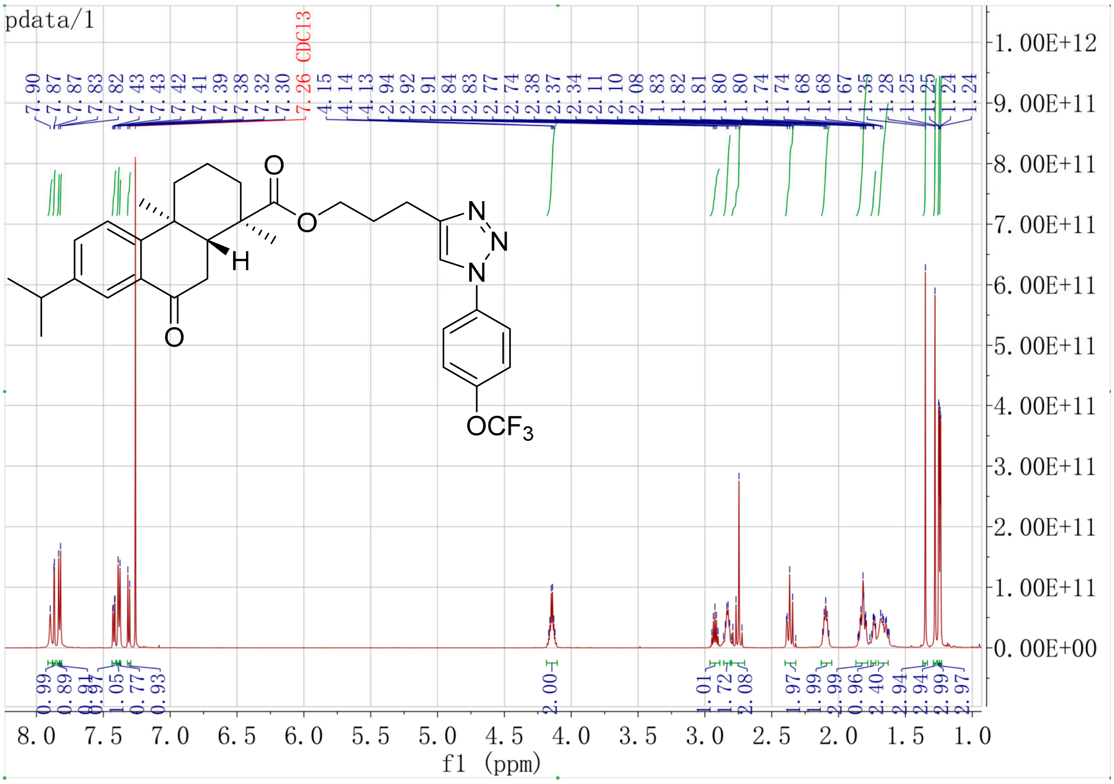

Figure S28-1. ^{1}H NMR spectrum of compound **33**

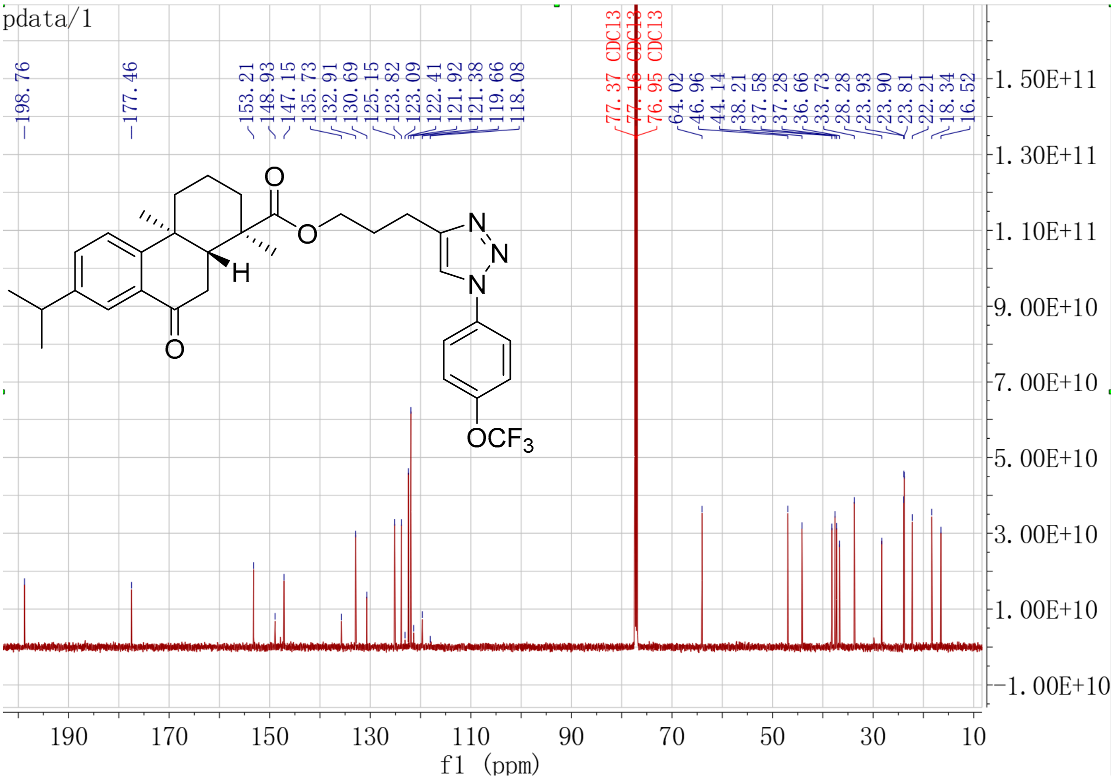

Figure S28-2. ^{13}C NMR spectrum of compound **33**

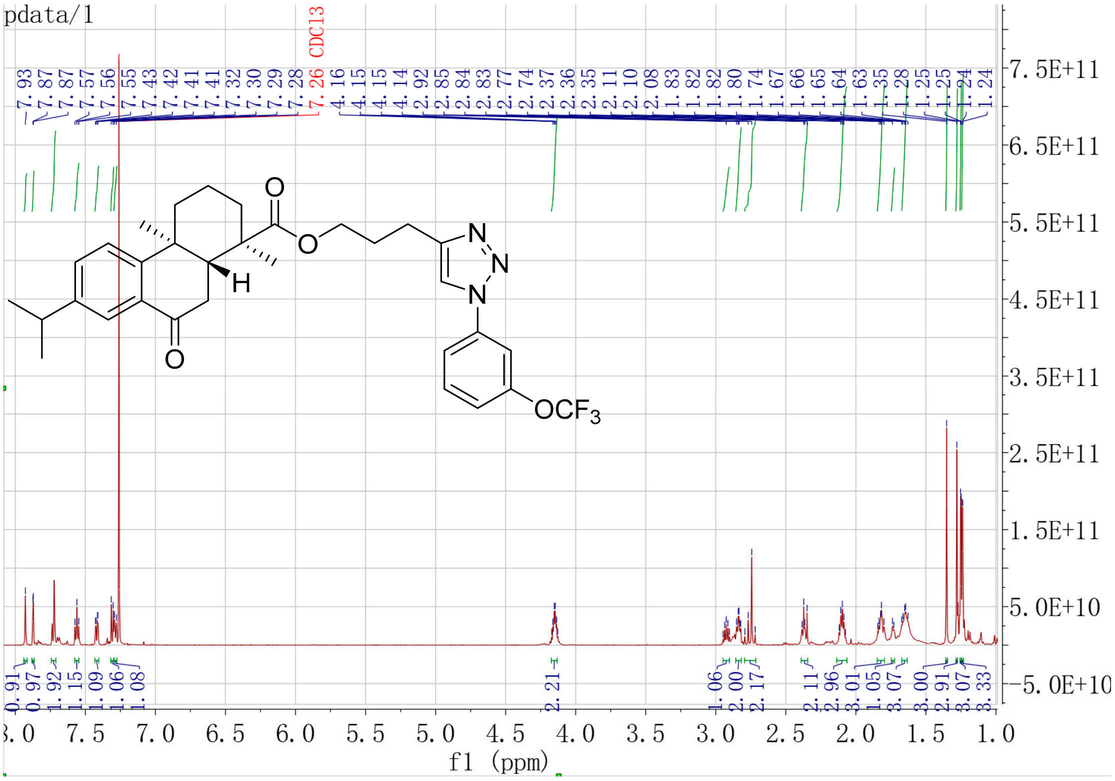

Figure S29-1. ^{1}H NMR spectrum of compound **34**

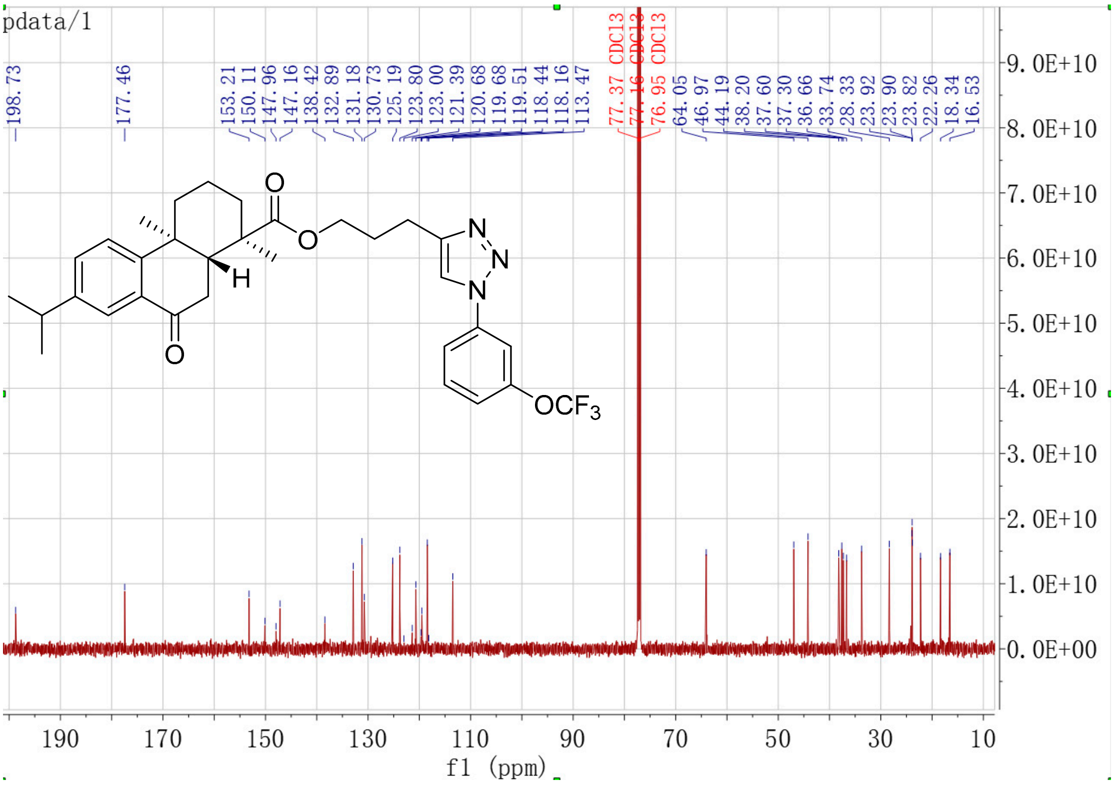

Figure S29-2. ^{13}C NMR spectrum of compound **34**

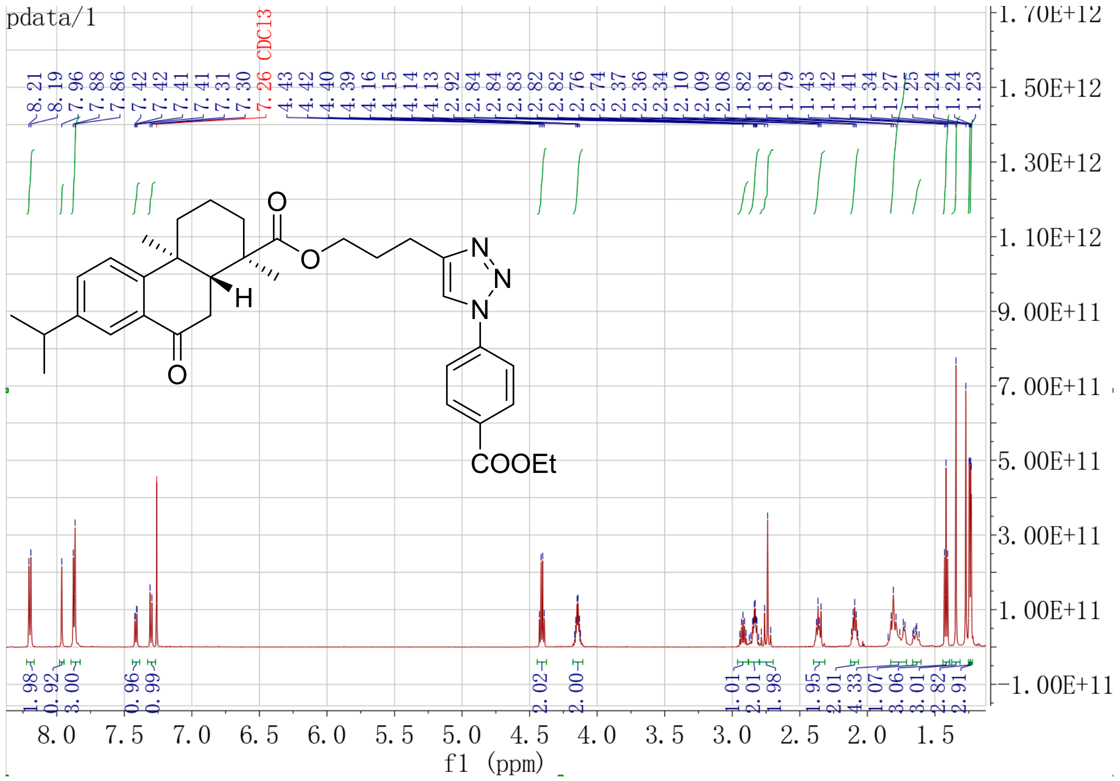

Figure S30-1. ^{1}H NMR spectrum of compound **35**

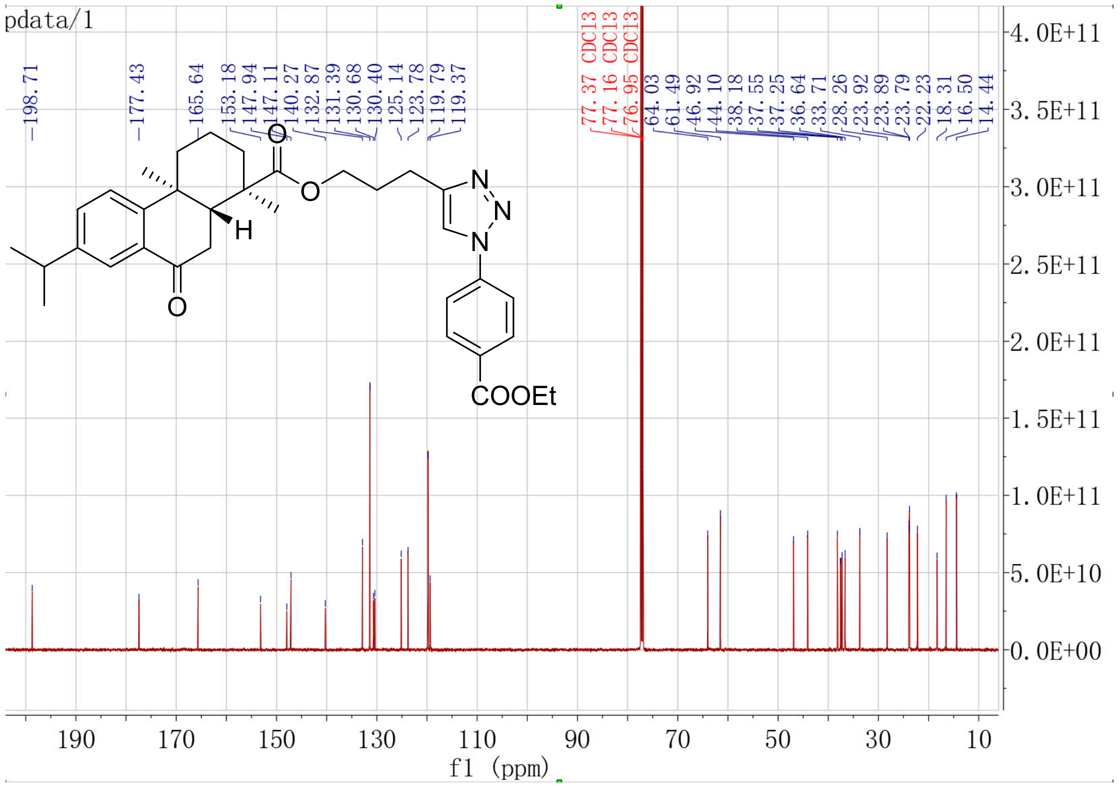

Figure S30-2. ^{13}C NMR spectrum of compound **35**

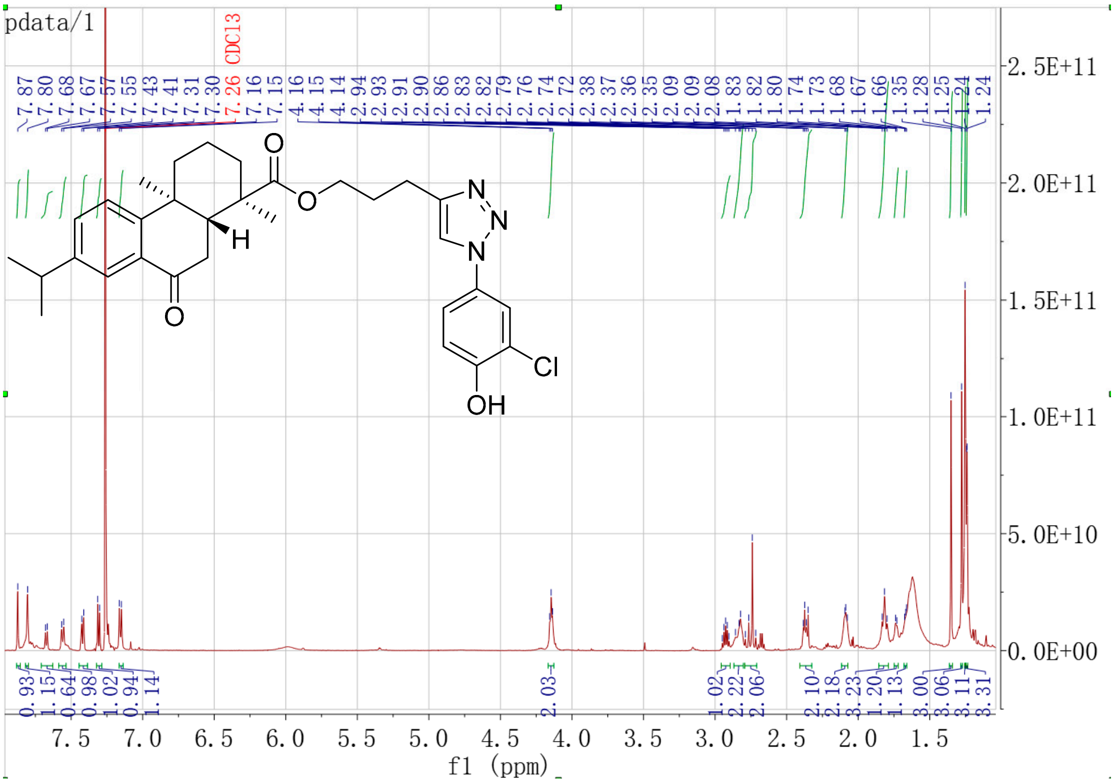

Figure S31-1. ^{1}H NMR spectrum of compound **36**

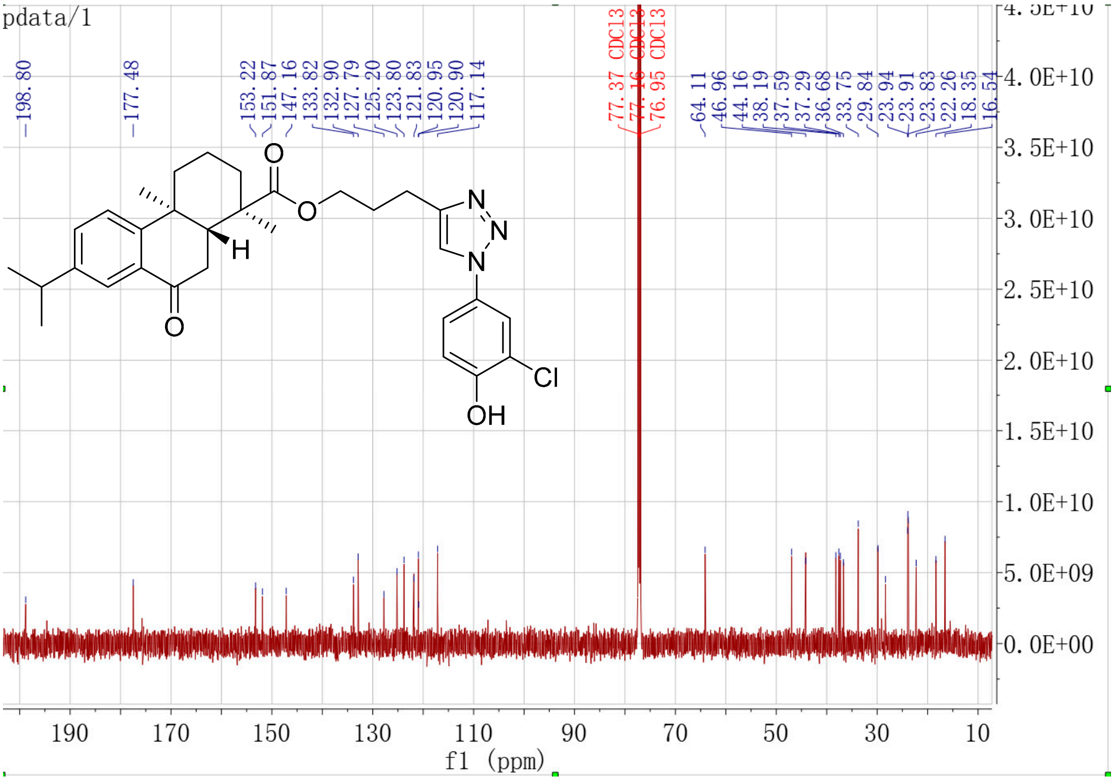

Figure S31-2. ^{13}C NMR spectrum of compound **36**

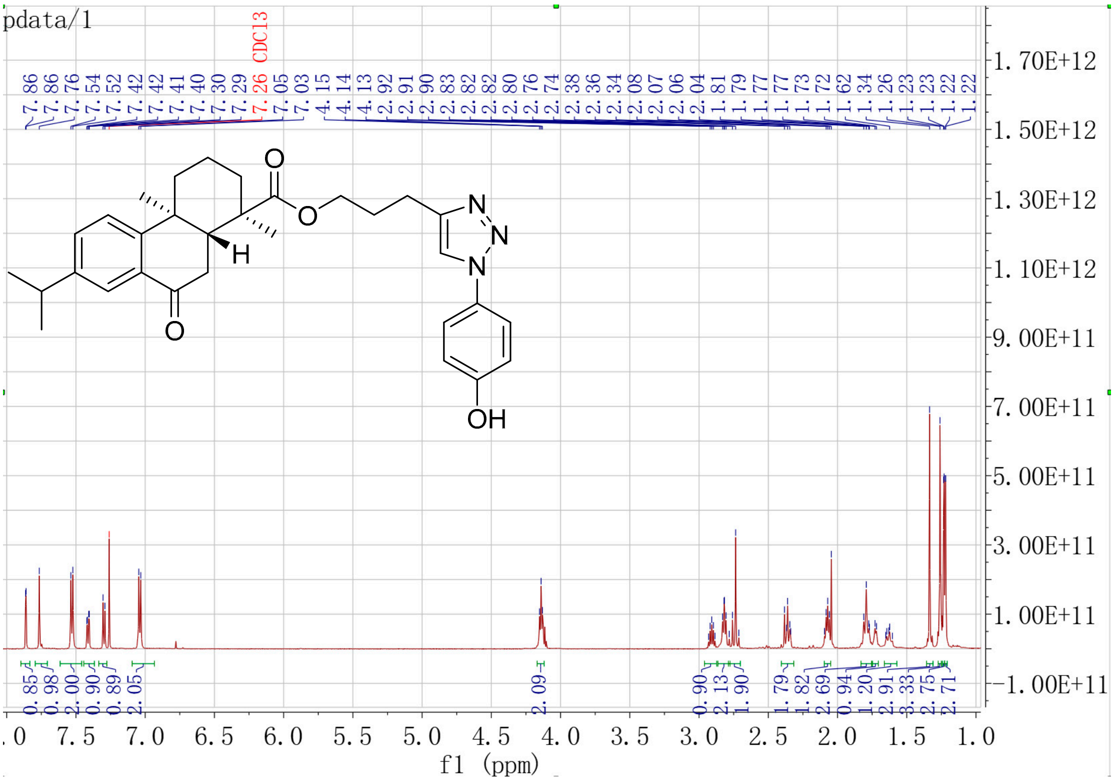

Figure S32-1. ^{1}H NMR spectrum of compound **37**

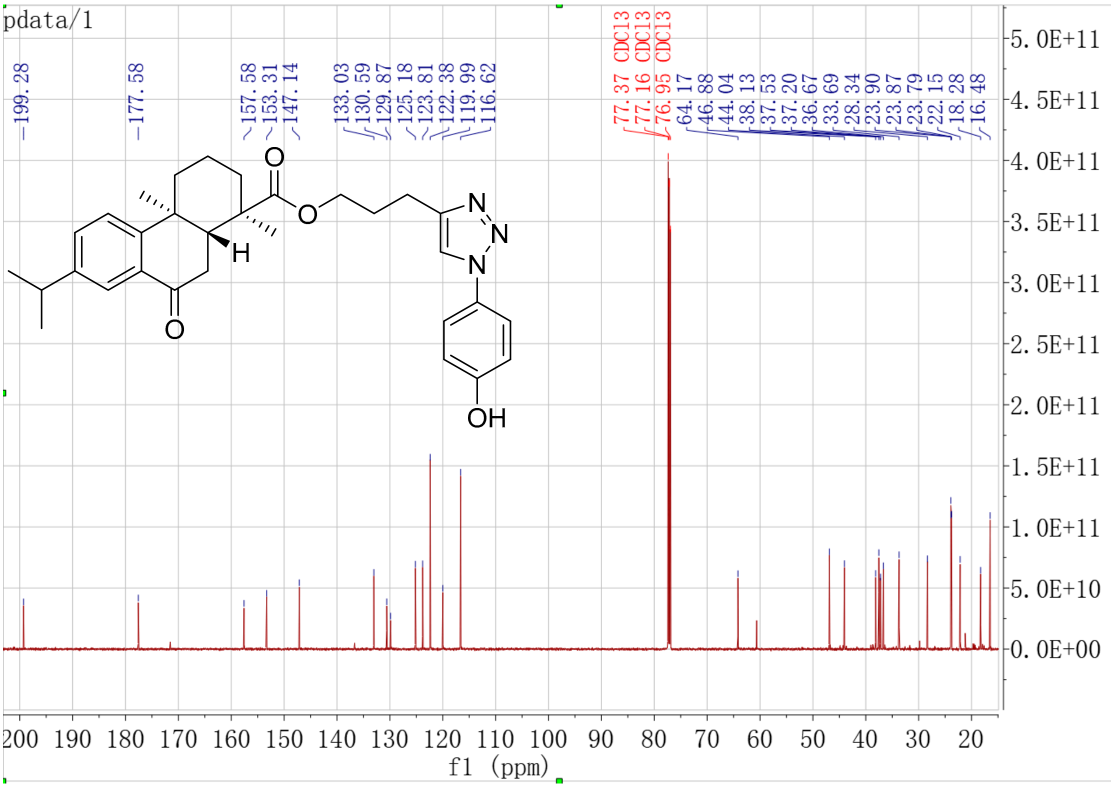

Figure S32-2. ^{13}C NMR spectrum of compound **37**

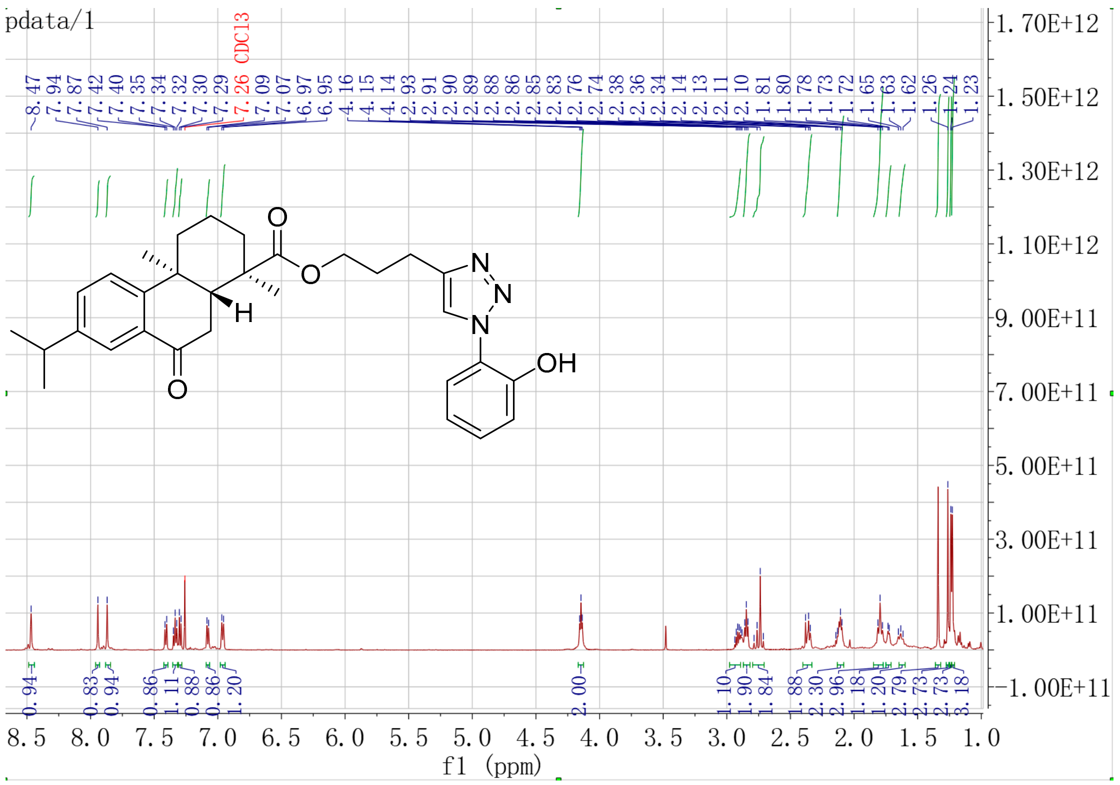

Figure S33-1. ^{1}H NMR spectrum of compound **38**

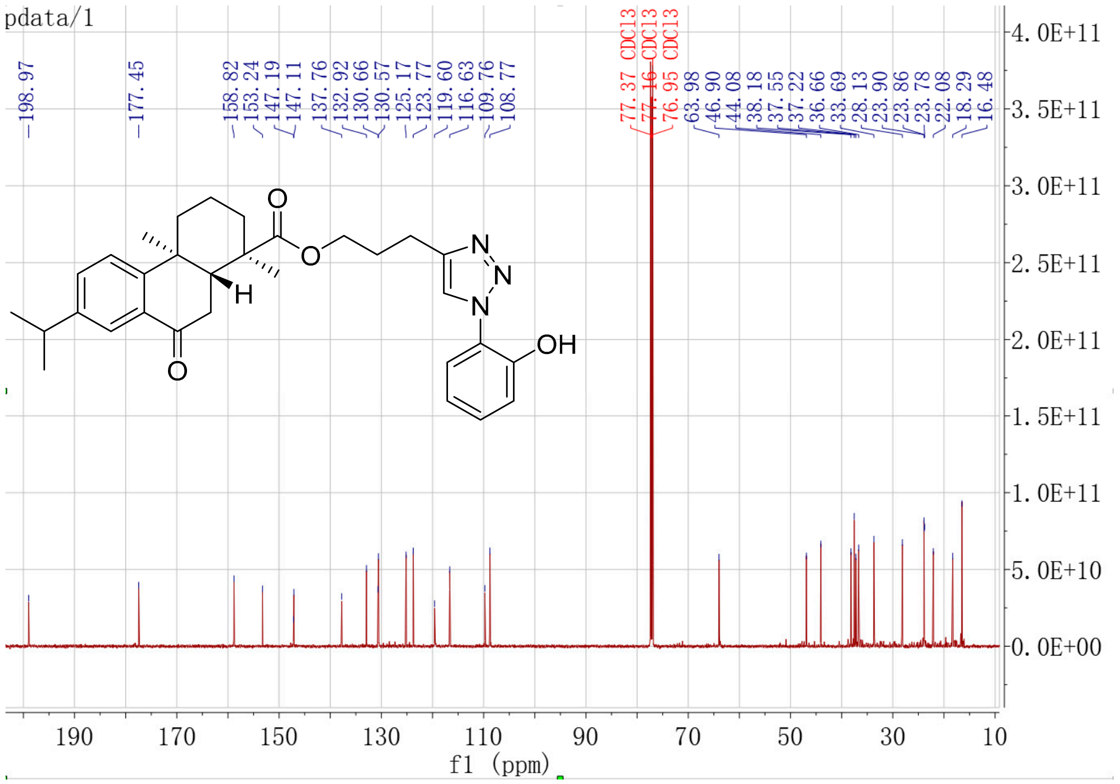

Figure S33-2. ^{13}C NMR spectrum of compound **38**

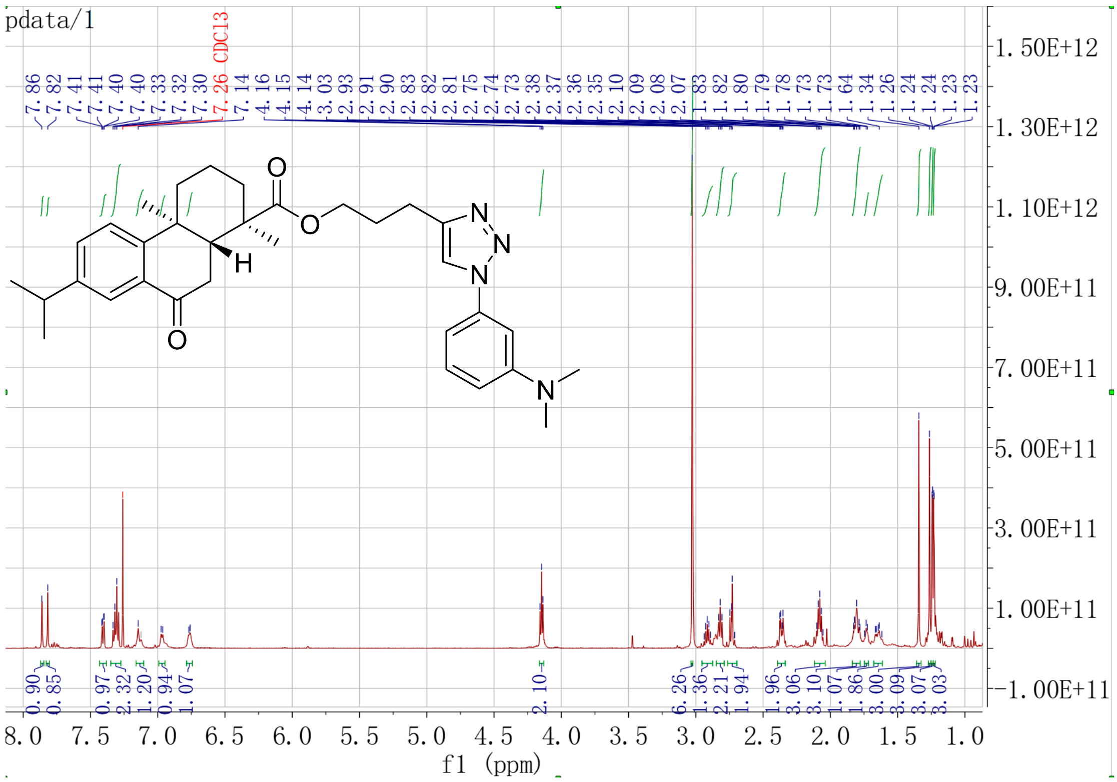

Figure S34-1. ^{1}H NMR spectrum of compound **39**

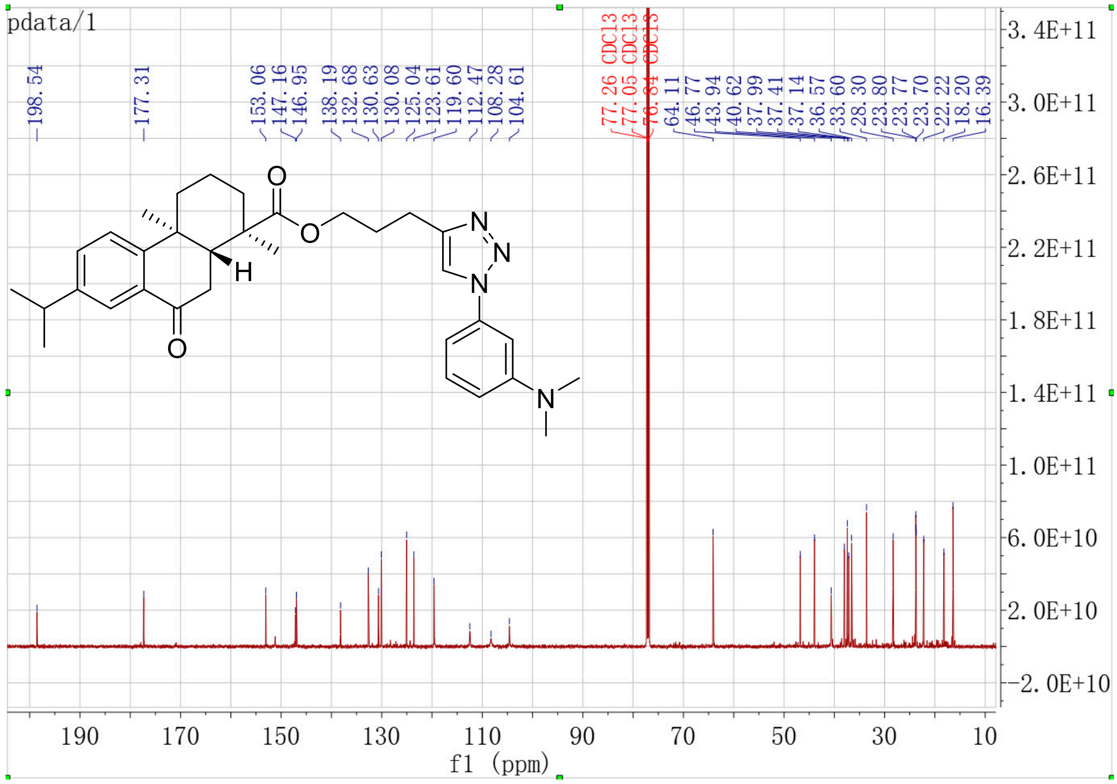

Figure S34-2. ^{13}C NMR spectrum of compound **39**
